# Transcriptional activation of *PHKG2* by *TP53* promotes ferroptosis through nuclear export of NRF2 in head and neck squamous cell carcinoma

**DOI:** 10.1038/s41419-025-07985-3

**Published:** 2025-08-30

**Authors:** Yalian Yu, Meng Luan, Jian Zang, Le Luo, Tianyi Wang, Tianci Wang, Yan Wang, Hongbo Wang

**Affiliations:** 1 Department of Otorhinolaryngology, The First Affiliated Hospital of China Medical University, Shenyang, PR China; 2 Department of Obstetrics and Gynecology, Shengjing Hospital of China Medical University, Shenyang, PR China; 3 Department of Interventional Radiology, Shengjing Hospital of China Medical University, Shenyang, PR China

**Keywords:** Head and neck cancer, Oncogenes

## Abstract

Head and neck squamous cell carcinoma (HNSCC) is a common malignancy with a poor prognosis despite multiple available treatments. Ferroptosis, an iron-dependent form of regulated cell death characterized by lipid peroxidation, has recently emerged as a promising strategy for cancer therapy, particularly in head and neck malignancies. However, its regulatory mechanisms remain largely unclear. In this study, we demonstrate that *TP53* transcriptionally activates *PHKG2*, which promotes ferroptosis. PHKG2 enhances the activity of protein phosphatase 1 (PP1) by phosphorylating PPP1R3B, disrupting its interaction with PP1C. Activated PP1 dephosphorylates NRF2, promoting its nuclear export and suppressing GPX4 transcription, thereby enhancing ferroptosis sensitivity. Both in vitro and in vivo, PHKG2 overexpression significantly suppressed tumor growth and increased lipid peroxidation levels. These findings define a previously unrecognized *TP53/PHKG2*–PP1–NRF2 signaling axis in the regulation of ferroptosis in HNSCC and suggest a novel therapeutic target.

## Introduction

Head and neck squamous cell carcinoma (HNSCC) arises from the squamous epithelium of the nasopharynx, oropharynx, oral cavity, hypopharynx, and larynx, accounting for over 90% of head and neck cancers [1]. It is the seventh most common malignancy worldwide, with approximately 600,000 new cases annually and a mortality rate of 40–50% [2]. Despite multimodal treatments such as surgery, radiotherapy, chemotherapy, targeted therapy, and immunotherapy [1, 3], the 5-year overall survival has remained unsatisfactory [3]. Due to the hidden anatomical sites involved, many patients are diagnosed at locally advanced or metastatic stages [4–6]. For unresectable locally advanced HNSCC, radiochemotherapy or immunotherapy is commonly used [3, 7], but the efficacy remains limited, necessitating new therapeutic targets and strategies to improve outcomes and preserve organ function.

Ferroptosis is a novel form of regulated cell death driven by iron-mediated lipid peroxidation [8, 9]. It is initiated by the Fenton reaction, where Fe^2+^ catalyzes hydrogen peroxide to form hydroxyl and lipid radicals that oxidize polyunsaturated fatty acids (PUFAs) in cellular membranes [10], ultimately leading to membrane disruption and cell death [10, 11]. Recent studies suggest that ferroptosis holds therapeutic potential in various cancers [12, 13]. Cancer cells often exhibit distinct metabolic profiles, increased oxidative stress, and elevated levels of metabolic intermediates compared to normal cells, rendering them more vulnerable to ferroptosis. In HNSCC, the high proliferative and metabolic rates of tumor cells require robust antioxidant defenses to avoid oxidative collapse [13]. The dependency on antioxidant systems, along with elevated intracellular iron and PUFA content, makes HNSCC cells particularly susceptible to ferroptosis [14]. Ferroptosis has been implicated in enhancing the sensitivity of HNSCC to radiotherapy and chemotherapy [15–17], drawing increasing attention to its regulatory network in head and neck cancers.

PHKG2 encodes the gamma-2 catalytic subunit of phosphorylase b kinase (PHK), a key enzyme in glycogen metabolism. It exhibits differential expression in tumors such as endometrial carcinoma [18], lung cancer [19], and renal clear cell carcinoma [20], and serves as a favorable prognostic marker. A study published in *PNAS* indicated that PHKG2 enhances lipid peroxidation via a TP53-dependent labile iron pool pathway, suggesting a role in ferroptotic regulation [21]. However, the expression pattern of PHKG2 in HNSCC and its mechanistic role in ferroptosis remain unclear.

In this study, we show that PHKG2 is a protective prognostic factor in HNSCC and that its expression negatively correlates with the ferroptosis-related antioxidant proteins NRF2 and GPX4. PHKG2 is transcriptionally activated by *TP53* and functions as a serine/threonine kinase that activates PP1. This promotes NRF2 nuclear export, suppresses NRF2/GPX4 signaling, reduces GSH levels, increases reactive oxygen species (ROS) accumulation, and sensitizes cells to ferroptosis. Our findings reveal a novel tumor-suppressive role of PHKG2 in HNSCC and provide mechanistic insight into a *TP53*–PHKG2 regulatory axis. This work expands our understanding of ferroptosis regulation and suggests potential therapeutic strategies targeting this pathway.

## Materials and methods

### Cell lines and culture

The human pharyngeal squamous cell carcinoma cell line FaDu and the highly metastatic nasopharyngeal carcinoma cell line 5–8F were obtained from the Shanghai Institute of Biochemistry and Cell Biology (Chinese Academy of Sciences, China). All cells were cultured at 37 °C in a humidified incubator with 5% CO₂. FaDu cells were maintained in Minimum Essential Medium (MEM; GIBCO, USA) and 5–8F cells in RPMI-1640 medium (GIBCO, USA), both supplemented with 10% fetal bovine serum (FBS; GIBCO, USA), 50 μg/mL penicillin, and 50 μg/mL streptomycin. Cells were routinely passaged using 0.25% trypsin-EDTA and tested negative for mycoplasma contamination.

### Lentiviral infection and stable cell line establishment

Lentiviral vectors carrying shRNAs targeting PHKG2 or *TP53*, as well as a non-targeting control shRNA (sh-NC), were synthesized by OBiO Technology (Shanghai, China). Cells were seeded in 6-well plates (4.0 × 10^4^ cells per well) and transduced with lentivirus at the recommended multiplicity of infection according to the manufacturer’s protocol. After 16 h of incubation at 37 °C, the culture medium was replaced with fresh complete medium. Seventy-two hours post-infection, cells were selected with 2 μg/mL puromycin for 7–10 days to establish stable cell lines. The shRNA sequences are listed in Supplementary File.

### Patient specimens

Tumor and adjacent normal tissue specimens were retrospectively collected from HNSCC patients who underwent surgical resection at the First Hospital of China Medical University between January 2019 and January 2020. All patients provided written informed consent prior to the collection of specimens, and the study was approved by the Scientific Research Ethics Committee of the First Hospital of China Medical University (Approval No. AF-SOP07-1.1-01).

Inclusion criteria were as follows: (1) HNSCC patients who were pathologically diagnosed at the First Hospital of China Medical University and underwent surgical treatment; (2) Patients with complete pathological slides of both cancerous and adjacent tissues post-surgery. Patients’ demographics, including age, gender, and clinical stage, were recorded. Exclusion criteria included: (1) Patients with concurrent or previous malignancies; (2) Patients who had received any preoperative anti-tumor interventions, including induction chemotherapy, radiotherapy, immunotherapy, or targeted therapy; (3) Patients with incomplete follow-up information after surgery. Since gender was identified as an influential factor in the bioinformatics analysis, all 57 patients included in this study were male to eliminate gender bias. No patients were lost to follow-up during the study period, and there were no cases of attrition.

### Data acquisition and bioinformatics analysis

HNSCC RNA-sequencing data and associated clinical information were downloaded from The Cancer Genome Atlas (TCGA) database using TCGA-Assembler. Gene expression data were normalized as fragments per kilobase of transcript per million mapped reads (FPKM). Sixty ferroptosis-related genes (listed in Supplementary File) were analyzed [22]. Differential expression analysis was conducted using the Limma package in R software (v3.6.1), and prognostic relevance was assessed using the Survival package. Statistical significance was set at *p* < 0.05.

### Animal models

All animal procedures were approved by the Animal Ethics Committee of China Medical University (Approval No. KT2023026). Female BALB/c nude mice (6 weeks old) were purchased from Beijing Vital River Laboratory Animal Technology Co., Ltd. and housed under a 12 h light/dark cycle at 22 ± 1 °C with 45–55% humidity and free access to food and water. 5–8F cells were subcutaneously injected into the right dorsal flank of nude mice (5 × 10^6^ cells in 0.2 mL PBS per mouse). Tumor dimensions were measured every 3 days, and tumor volumes were calculated as (length × width^2^)/2. After 4 weeks, mice were euthanized, and tumors were harvested for further analysis. To minimize bias, mice were randomly assigned to experimental groups using a computer-generated randomization protocol. Randomization was performed after acclimatization, ensuring equal distribution based on body weight to maintain homogeneity between groups.

### Establishment of sh-PHKG2 xenograft model

Nude mice were randomly divided into six groups: Control+DMSO, NC+DMSO, sh-PHKG2+DMSO, Control+Erastin, NC+Erastin, and sh-PHKG2+Erastin. Cells were injected subcutaneously as described above. One week after inoculation, mice were intraperitoneally injected with Erastin (15 mg/kg) or DMSO every other day for 20 days. Tumor growth was monitored throughout the treatment period.

### Establishment of the oe-PHKG2 and Pifithrin-α treatment model

Mice inoculated with parental, vector-transfected, or PHKG2-overexpressing 5–8 F cells were divided into six subgroups. After tumor establishment, mice received intraperitoneal injections of Pifithrin-α (10 mg/kg, Macklin, China, Cat#P863895) or DMSO every other day for 20 days. Tumor volume was recorded every 3 days, and tumors were collected at the endpoint.

### Immunohistochemistry (IHC) and scoring

Formalin-fixed, paraffin-embedded tumor tissues were sectioned at 4–6 μm thickness, deparaffinized in xylene, and rehydrated through graded ethanol. Antigen retrieval was performed in EDTA buffer (pH 8.0) at 95 °C °C for 20 min. Endogenous peroxidase activity was blocked with 3% hydrogen peroxide for 15 min. Sections were blocked with 1% bovine serum albumin and incubated overnight at 4 °C with primary antibodies diluted in blocking solution. After washing, slides were incubated with HRP-conjugated secondary antibodies for 1 h at room temperature. Color was developed with DAB, and sections were counterstained with hematoxylin. Staining intensity and percentage of positive cells were scored semi-quantitatively, and the final score was calculated as the product of intensity and proportion scores. Expression levels were categorized as low (scores below the median value) or high (scores above the median value) [23]. Two independent pathologists blinded to clinical data evaluated all specimens.

### Alamar blue cell viability assay

5 × 10^3^ cells per well were seeded into 96-well plates in triplicate and transduced with PHKG2-shRNA lentivirus. After 24 h, cells were treated with various concentrations of Erastin (Glpbio, USA), RSL3, and FIN56 for an additional 24 h. Alamar Blue reagent was added according to the manufacturer’s instructions. Absorbance was measured at 450 nm using a microplate reader (BioTek Instruments, USA).

### Lipid peroxidation and redox status assays

#### Malondialdehyde (MDA) detection

Cells were harvested, washed twice with PBS, and lysed in extraction buffer (1 mL per 5 × 10⁶ cells). Cell lysates were sonicated (20% power, 3 s on/10 s off, 30 cycles) and centrifuged at 8000 × *g* for 10 min at 4 °C. The supernatant was used to measure absorbance at 450 nm, 532 nm, and 600 nm. MDA content (nmol/mg protein) was calculated using the standard curve according to the manufacturer’s protocol (Wanleibio, China).

#### Reduced Glutathione (GSH) Detection

Cells (≥1 × 10⁶) were lysed after two PBS washes by resuspending the pellet in triple the volume of lysis buffer. After 2–3 freeze–thaw cycles, samples were centrifuged at 8000 g for 10 min, and supernatants were collected. GSH levels were determined by measuring absorbance at 412 nm and calculated based on a standard curve.

#### FerroOrange probe for intracellular Fe^2+^ detection

Cells were stained with 1 μM FerroOrange fluorescent probe (MaokangBio, China) diluted in serum-free medium, incubated at 37 °C for 30 min in the dark, and observed under a fluorescence microscope (IX53, Olympus, Japan).

#### Intracellular ROS detection

Cells were incubated with 10 μM dihydroethidium (DHE; Macklin, China) at 37 °C for 30 min in the dark. After washing with fresh medium, images were captured using a fluorescence microscope (IX53, Olympus, Japan).

### Transmission electron microscopy (TEM)

Cells were fixed overnight in 2.5% glutaraldehyde at 4 °C, post-fixed in 1% osmium tetroxide for 1 h at room temperature, dehydrated through a graded ethanol series, embedded in epoxy resin, and sectioned into ultrathin slices (70 nm). Sections were stained with uranyl acetate and lead citrate and examined using a transmission electron microscope (Hitachi 7800, Japan).

### Quantitative real-time PCR (qRT-PCR)

Total RNA was extracted using the RNAsimple Total RNA Kit (Takara, Japan). cDNA synthesis was performed using the PrimeScript™ RT Reagent Kit with 500 ng RNA. qPCR was conducted using the One Step SYBR PrimeScript™ PLUS RT-PCR Kit (Takara, Japan) on a CFX96 Real-Time PCR Detection System (Bio-Rad, USA). GAPDH served as an internal control. Relative gene expression was calculated using the 2^−ΔΔCt method. The primer sequences used for qRT-PCR are listed in Supplementary File.

### Western blot analysis

Protein was extracted using lysis buffer containing protease inhibitors and quantified with a BCA Protein Assay Kit (Wanleibio, China). Equal amounts of protein were separated by 12% SDS-PAGE and transferred to PVDF membranes (Millipore, USA). Membranes were blocked with 5% non-fat milk and incubated overnight at 4 °C with the following primary antibodies: anti-TP53 (Cell Signaling Technology, USA), anti-PHKG2 (ABclonal, USA), anti-NRF2 (Wanleibio, China), anti-Histone H3 (Wanleibio, China), anti-β-actin (Wanleibio, China), anti-GPX4 (Cell Signaling Technology, USA), anti-PPP1R3B (Proteintech, China), anti-PP1C (Santa Cruz, USA) and anti-p-Ser (Santa Cruz, USA), all diluted 1:1000. After washing, membranes were incubated with HRP-conjugated goat anti-rabbit secondary antibody (1:5000, Wanleibio, China) for 2 h at room temperature. Signals were detected by enhanced chemiluminescence (ECL) and visualized. Original, uncropped Western blot images are provided in Supplementary Western Blot.

### Dual-luciferase reporter assay

The 2 kb promoter region of PHKG2 was cloned into the pGL3-Basic vector. 5–8 F cells were co-transfected with the promoter construct or empty vector along with a TP53 overexpression plasmid and the pRL-TK Renilla luciferase control vector. After 48 h, luciferase activities were measured using the Dual-Luciferase Reporter Assay System (Promega, USA). Firefly luciferase activity was normalized to Renilla luciferase activity.

### Chromatin immunoprecipitation (ChIP) assay

5–8F cells were cross-linked with 1% formaldehyde and quenched with 125 mM glycine. Chromatin was sonicated to 500–1000 bp fragments, precleared with Protein A+G Agarose beads, and immunoprecipitated with anti-TP53 antibody or IgG control. After sequential washing, DNA-protein complexes were eluted, cross-links reversed, and DNA purified. ChIP-enriched DNA was analyzed by PCR (gel electrophoresis) and real-time qPCR using SYBR Green chemistry. Data were normalized to input DNA and analyzed using the 2^−ΔΔCt^ method.

### Co-immunoprecipitation (Co-IP) assay

Cells were lysed with precooled buffer containing 1% PMSF. Equal amounts of lysates were incubated with AminoLink® resin coupled to antibodies against PPP1R3B (Proteintech, 14190-1-AP), PP1C (Santa Cruz, sc-515943), PHKG2 (Proteintech, 15109-1-AP), or phospho-Ser (Santa Cruz, sc-81514). Immunoprecipitates were washed, eluted, separated by SDS-PAGE, and analyzed by Western blotting. Original, uncropped Western blot images are provided in Supplementary Western Blot.

### Protein phosphatase 1 (PP1) activity assay

PP1 activity was determined using a PP1 Activity Assay Kit (GENMED, China) following the manufacturer’s instructions. Phosphate release was measured colorimetrically, and activity was normalized to protein concentration and reaction time.

### Immunofluorescence staining and confocal microscopy

Cells grown on coverslips were fixed with 4% paraformaldehyde, permeabilized with 0.1% Triton X-100, and blocked. Cells were incubated with primary antibodies against NRF2 (1:50) and PHKG2 (1:50) overnight at 4 °C, followed by Alexa Fluor 488- or 555-conjugated secondary antibodies. Nuclei were counterstained with DAPI. Images were captured using a confocal microscope (BX53, Olympus, Japan).

### Statistical analysis

All experiments were repeated independently at least three times. Data are presented as mean ± standard deviation. Statistical analyses were performed using SPSS 27.0 and GraphPad Prism 9.0. Differences between the two groups were analyzed using an unpaired Student’s *t*-test, and multiple group comparisons were assessed by one-way or two-way ANOVA, followed by post hoc tests where appropriate. *P* values < 0.05 were considered statistically significant.

## Results

### PHKG2 expression is associated with ferroptosis and a favorable prognosis in HNSCC

**Fig. 1:**
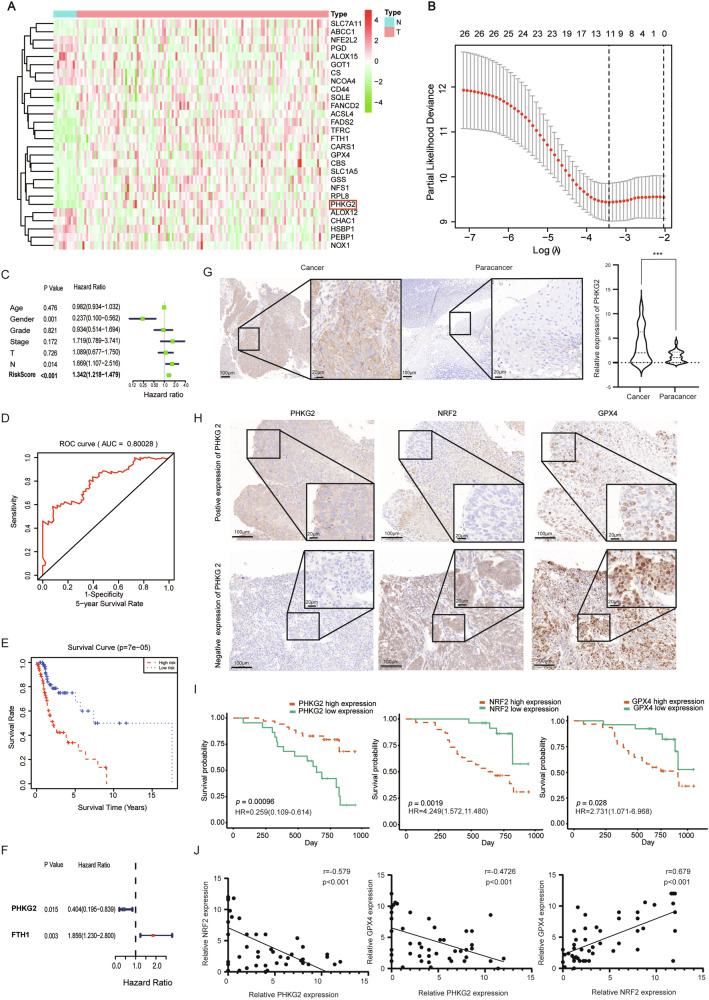
PHKG2 expression correlates with prognosis in HNSCC patients. The expression profiles of ferroptosis-related genes, including PHKG2, were identified in HNSCC tissues from the TCGA dataset (**A**). Eleven key genes were selected via LASSO regression analysis to establish a prognostic risk model (**B**). Univariate Cox regression analysis showing correlations between risk factors (including age, gender, clinical stage, and TNM staging) and patient prognosis (**C**). ROC curves evaluating the predictive ability of the risk model for 5-year patient survival (**D**). Kaplan–Meier survival analysis demonstrating significantly poorer overall survival (OS) in the high-risk group compared to the low-risk group (**p < 0.01) (**E**). Kaplan–Meier curves highlighting that high PHKG2 expression (HR = 0.404, *p < 0.05) is associated with better prognosis, whereas high FTH1 expression (HR = 1.856, *p < 0.05) indicates poorer outcomes (**F**). Representative immunohistochemical (IHC) staining images illustrate moderate-to-high PHKG2 expression in HNSCC tissues compared to adjacent non-tumor tissues (***p < 0.001) (**G**). Representative IHC staining demonstrating an inverse correlation between PHKG2 expression and the expression levels of NRF2 (nuclear) and GPX4 proteins in HNSCC tissues (**H**). Kaplan–Meier survival curves depicting the association of PHKG2, NRF2, and GPX4 expression levels with patient prognosis (**I**). Correlation analyses demonstrating inverse relationships between PHKG2 expression and the ferroptosis-related proteins NRF2 and GPX4 (**J**).

Analysis of the TCGA database revealed differential expression of 28 ferroptosis-related genes between HNSCC tissues and adjacent non-tumorous tissues, including *PHKG2, FTH1*, and *GPX4* (Fig. 1A). LASSO regression identified 11 genes to construct a risk prediction model (Fig. 1B). Cox regression showed that male gender was a protective factor, whereas lymph node metastasis and high-risk score were associated with worse outcomes (Fig. 1C). The 5-year ROC curve suggested the model had good predictive accuracy (Fig. 1D).

Survival analysis demonstrated significantly poorer outcomes in high-risk patients (Fig. 1E). Single-gene survival analysis indicated *PHKG2* was associated with better prognosis, while *FTH1* predicted worse outcomes (Fig. 1F). Of note, *PHKG2* had the most negative weight in the LASSO model (coefficient = −0.1342) (Supplementary File), suggesting its strong protective role. Previous studies have reported that *PHKG2* enhances cellular sensitivity to Erastin-induced ferroptosis, although the underlying mechanisms remain unclear [21]. Therefore, we focused on *PHKG2* as the primary gene of interest in the present study.

### PHKG2 expression correlates with tumor stage and negatively regulates NRF2/GPX4

To minimize sex-related confounders, 57 male HNSCC patients who underwent surgical resection were included in the cohort. The cohort consisted of 50 patients with hypopharyngeal cancer and 7 with nasopharyngeal cancer. The patients’ ages ranged from 32 to 80 years, with a median age of 60. Tumor diameters varied from 14.0 mm to 75.0 mm (median diameter: 37.0 mm).

PHKG2 expression was significantly associated with tumor size (T stage), lymph node status (N stage), and overall clinical stage in HNSCC patients (*p* < 0.05), as shown in Table 1. Immunohistochemistry (IHC) analysis confirmed higher PHKG2 expression in tumor tissues compared to adjacent normal tissues (*p* < 0.001) (Fig. 1G). Univariate Cox analysis showed PHKG2 (HR = 0.259, 95% CI: 0.109–0.614, *p* < 0.05) expression was a favorable prognostic marker, while high levels of NRF2 (HR = 4.249, 95% CI: 1.572–11.480, *p* < 0.05) and GPX4 (HR = 2.731, 95% CI: 1.071–6.968, *p* < 0.05) indicated poor outcomes (Fig. 1H, I). Spearman’s correlation analysis showed a negative association between PHKG2 expression and both NRF2 (*r* = −0.579, *p* < 0.001) and GPX4 (*r* = −0.4726, *p* < 0.001). In contrast, a positive correlation was observed between nuclear NRF2 and GPX4 expression (*r* = 0.679, *p* < 0.001) (Fig. 1J).

**Table 1:** Clinical features of HNSCC patients.

Clinical features	PHKG2		
	Over expression	Low expression	*P* value
Year			0.705
≥60 yr	21	9	
<60 yr	14	13	
T Stage			0.012^*^
T1–2	12	5	
T3–4	23	17	
N Stage			0.048^*^
N0-1	15	5	
N2–3	20	17	
Clinical stage			0.001^***^
I–II	12	2	
III–IV	23	20	

Data are presented as number of patients. Bold values indicate statistically significant results (*p* < 0.05). *p < 0.05, ***p < 0.001.

### *PHKG2* enhances ferroptosis sensitivity by modulating oxidative stress in HNSCC cells

**Fig. 2:**
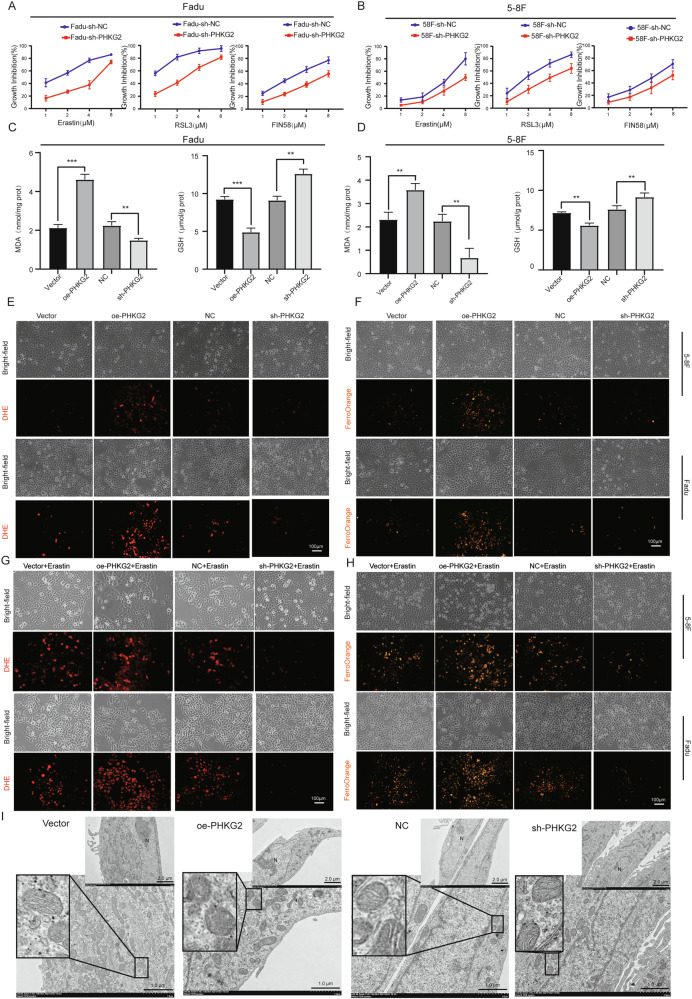
*PHKG2* promotes ferroptosis in HNSCC cells. *PHKG2* knockdown significantly reduced the sensitivity of FaDu and 5–8 F cells to the ferroptosis inducer Erastin (**A**, **B**). Overexpression of *PHKG2* increased intracellular MDA levels and reduced GSH levels in FaDu cells and 5–8F cells (**p < 0.01, ***p < 0.001) (**C**, **D**). DHE and FerroOrange fluorescence assays indicated that *PHKG2* overexpression elevated intracellular ROS and Fe^2+^ levels in both FaDu and 5–8F cells, whereas *PHKG2* knockdown produced opposite effects (**E**, **F**). Under Erastin-induced ferroptotic conditions, *PHKG2* overexpression further enhanced ROS and Fe^2+^ accumulation, while *PHKG2* knockdown significantly reversed these effects (**G**, **H**). TEM images showing mitochondrial damage in HNSCC cells. *PHKG2* overexpression leads to cristae fragmentation and membrane disruption, while knockdown preserves normal mitochondrial structure and abundance. Nuclear morphology remains unchanged (**I**).

Baseline expression of *PHKG2* was confirmed in 5–8F and FaDu cells via qRT-PCR. Among three tested siRNA constructs, the most efficient *PHKG2*-targeting sequence was selected (Fig. S1A). Alamar Blue assays showed that *PHKG2* knockdown significantly reduced sensitivity to ferroptosis inducers (Erastin, RSL3, FIN56), while IC50 concentrations were used for follow-up experiments (Fig. 2A, B).

Lipid peroxidation assays revealed that *PHKG2* silencing reduced intracellular malondialdehyde (MDA) and increased glutathione (GSH) levels, suggesting a shift in redox balance (Fig. 2C, D). Conversely, *PHKG2* overexpression elevated ROS and Fe^2+^ levels, while knockdown reduced them (Fig. 2E, F). Upon Erastin, RSL3, and FIN56 treatment, *PHKG2* overexpression further amplified ROS and Fe^2+^ accumulation; silencing had the opposite effect (Figs. 2G, H and S1B–I). Transmission electron microscopy (TEM) showed typical ferroptotic features in mitochondria upon *PHKG2* overexpression (e.g., disrupted cristae and membrane rupture) (Fig. 2I). These results suggest that *PHKG2* promotes ferroptosis by enhancing oxidative stress in HNSCC cells.

### *TP53* transcriptionally activates *PHKG2* and suppresses nuclear NRF2 protein levels

**Fig. 3:**
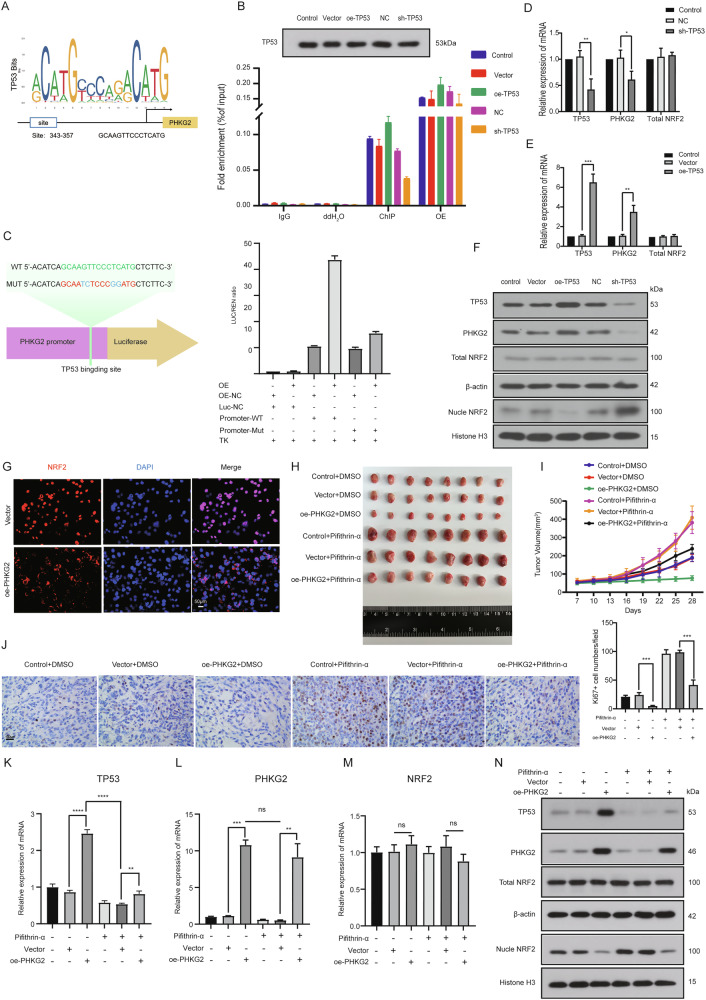
*TP53* transcriptionally activates *PHKG2* to suppress nuclear NRF2 expression in vitro and in vivo. JASPAR analysis predicts potential *TP53* binding sites within the *PHKG2* promoter region (**A**). ChIP-PCR experiments verified direct binding of *TP53* to the *PHKG2* promoter, with significantly increased enrichment observed upon *TP53* overexpression (**B**). Dual-luciferase reporter assays confirmed *TP53*-mediated transcriptional activation of *PHKG2* (**C**). qRT-PCR analysis indicated that *TP53* positively regulated *PHKG2 mRNA* levels, without affecting *NRF2 mRNA* expression (*p < 0.05, **p < 0.01, ***p < 0.001) (**D**, **E**). Western blot analysis revealed that *TP53* upregulated *PHKG2* protein expression and decreased nuclear NRF2 levels, while total NRF2 protein remained unchanged (**F**). Confocal microscopy confirmed decreased nuclear and increased cytoplasmic NRF2 following *PHKG2* overexpression (**G**). Xenograft mouse models showed *PHKG2* overexpression significantly inhibited tumor growth, whereas administration of the TP53 inhibitor Pifithrin-α reversed this effect, leading to increased tumor size and enhanced Ki67 expression (**p < 0.01, ***p < 0.001) (**H**–**J**). *qRT****-****PCR* analysis showing that *TP53* and *PHKG2 mRNA* levels are elevated in tumors from the *oe-PHKG2* group. Treatment with the TP53 inhibitor Pifithrin-α partially reduces these increases, but does not completely abrogate the upregulation (**p < 0.01, ***p < 0.001) (**K**, **L**). *NRF2 mRNA* levels remained unaffected (**M**). Western blot further confirmed reduced nuclear NRF2 protein levels upon TP53-mediated PHKG2 activation (**N**).

JASPAR analysis predicted a *TP53* binding motif within the *PHKG2* promoter (Fig. 3A). This was validated by ChIP-PCR and gel electrophoresis (Figs. 3B and S2A, B). Dual-luciferase reporter assays confirmed that *TP53* directly activated *PHKG2* promoter activity (Fig. 3C). *TP53* overexpression significantly increased *PHKG2* mRNA and protein expression (Fig. 3D–F), and reduced nuclear NRF2 protein levels without altering its total or mRNA levels (Fig. 3G).

In vivo, *PHKG2*-overexpressing xenografts exhibited significantly smaller tumor volumes (Fig. 3H, I) and reduced Ki67 expression, even under *TP53* inhibition (Fig. 3J). qRT-PCR analysis of xenograft tumor tissues revealed that overexpression of *PHKG2* led to a marked increase in both *TP53* and *PHKG2 mRNA* levels compared to controls (Fig. 3K, L). Administration of the *TP53* inhibitor Pifithrin-α partially attenuated this upregulation but did not fully abolish it, suggesting that *PHKG2* may enhance *TP53* expression through both *TP53-*dependent and -independent mechanisms. In contrast, *NRF2 mRNA* levels remained largely unchanged across all groups (Fig. 3M). Western blotting showed that *TP53* inhibition increased nuclear NRF2 protein levels, which could be reversed by *PHKG2* overexpression (Fig. 3N). Collectively, these findings suggest *TP53* transcriptionally activates *PHKG2*, which in turn suppresses NRF2 nuclear localization.

### PHKG2 promotes NRF2 nuclear export through PP1 activation

**Fig. 4:**
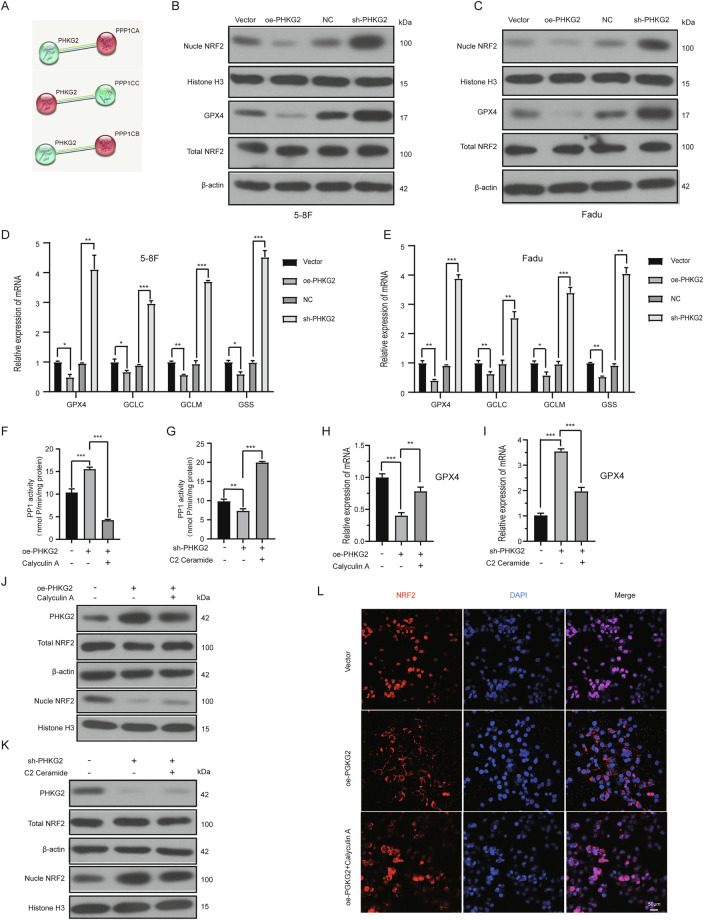
PHKG2 activates PP1, leading to reduced nuclear NRF2 expression. STRING analysis predicted potential interactions between PHKG2 and the three catalytic subunits (γ, α, β) of PP1 (**A**). Western blot results showed that PHKG2 overexpression decreased nuclear NRF2 and GPX4 protein levels in 5–8F and FaDu cells, while PHKG2 knockdown exerted the opposite effects; total NRF2 remained unchanged (**B**, **C**). *qRT-PCR* analysis indicated that *PHKG2* regulates *GPX4* at the transcriptional level. *PHKG2* overexpression decreased mRNA levels of *GPX4, GCLC, GCLM*, and *GSS*, while *PHKG2* knockdown increased their expression (*p < 0.05, **p < 0.01, ***p < 0.001) (**D**, **E**).PP1 activity assays confirmed increased PP1 activation by PHKG2 overexpression, reversed by the PP1 inhibitor Calyculin A; PP1 activation was decreased following PHKG2 knockdown and restored by PP1 activator C2 Ceramide (**p < 0.01, ***p < 0.001) (**F**, **G**). *qRT-PCR* results showed that *PHKG2* overexpression decreased *GPX4* mRNA expression, which was reversed by Calyculin A. Conversely, *PHKG2* knockdown increased *GPX4* mRNA, and this effect was suppressed by C2 Ceramide (**p < 0.01, ***p < 0.001) (**H**, **I**). Western blotting further verified that changes in PP1 activity inversely correlated with nuclear NRF2 protein expression (**J**, **K**). Confocal microscopy demonstrated that PP1 inhibitor treatment reversed the nuclear export of NRF2 induced by PHKG2 overexpression (**L**).

To investigate how PHKG2 regulates NRF2 nuclear localization, STRING database analysis was performed and predicted potential interactions between PHKG2 and the three catalytic subunits of protein phosphatase 1 (PP1): α, β, and γ (Fig. 4A). Further phosphorylation site prediction using iGSP1.0 software revealed potential PHKG2-mediated phosphorylation sites on PP1, suggesting a possible regulatory link (Fig. S2C). Given that previous studies have shown PP1 promotes nuclear export of NRF2 by dephosphorylation [24], we hypothesized that PHKG2 may influence NRF2 subcellular distribution via modulation of PP1 activity.

Functional experiments further substantiated this hypothesis. Western blot analysis demonstrated that PHKG2 overexpression significantly reduced nuclear NRF2 and its downstream effector GPX4, whereas knockdown of PHKG2 produced the opposite effect. Notably, total NRF2 protein levels remained largely unchanged (Fig. 4B, C), indicating that PHKG2 primarily modulates NRF2 subcellular distribution rather than its overall expression.

*RT-PCR* results suggested that *PHKG2* also suppresses the expression of *GPX4* at the transcriptional level. In addition, the mRNA expression of GSH-synthesizing enzyme *GSS*, as well as the catalytic and modifier subunits of glutamate cysteine ligase, *GCLC* and *GCLM*, was inhibited (Fig. 4D, E). These results imply that *PHKG2* may influence the expression of NRF2 downstream target genes (*GPX4*, *GCLC*, and *GCLM*) through regulating NRF2 nuclear localization.

We next evaluated whether these effects were mediated through changes in PP1 activity. In cells overexpressing *PHKG2*, PP1 enzymatic activity was markedly increased, accompanied by decreased nuclear NRF2 and reduced *GPX4* expression. This activation was effectively blocked by the PP1-specific inhibitor Calyculin A, which concurrently restored nuclear NRF2 levels and *GPX4* expression (Fig. 4F, H, J). Conversely, silencing *PHKG2* suppressed PP1 activity, resulting in increased nuclear NRF2 protein and upregulated *GPX4* mRNA levels. This effect could be reversed by treatment with the PP1 activator C2 Ceramide, which again reduced nuclear NRF2 expression and *GPX4 mRNA* level (Fig. 4G, I, K). These reciprocal responses confirmed a regulatory link between PHKG2-mediated PP1 activation and NRF2 nuclear dynamics.

Immunofluorescence imaging further reinforced these findings. In cells with PHKG2 overexpression, NRF2 localization was predominantly cytoplasmic, suggesting enhanced nuclear export. Importantly, Calyculin A treatment reversed this effect, leading to nuclear re-accumulation of NRF2 (Fig. 4L). These changes were visually consistent with the biochemical data. Collectively, our results indicate that PHKG2 promotes PP1 activation, which facilitates the nuclear export of NRF2, thereby suppressing its nuclear retention and downstream antioxidant signaling.

### PHKG2 promotes ferroptosis by suppressing the NRF2/GPX4 axis in vitro and in vivo

To further delineate the role of PHKG2 in ferroptosis regulation, we explored whether its effects were mediated through NRF2 signaling. Cells were treated with erastin to induce ferroptosis, either alone or in combination with Carnosol, a known NRF2 activator. As expected, Carnosol treatment elevated nuclear NRF2, *GPX4 mRNA* and GPX4 protein levels, increased intracellular GSH levels, and upregulated the mRNA expression of *GCLC, GCLM*, and *GSS*. Concurrently, it reduced MDA, ROS, and Fe^2+^ accumulation, indicative of ferroptosis inhibition.

**Fig. 5:**
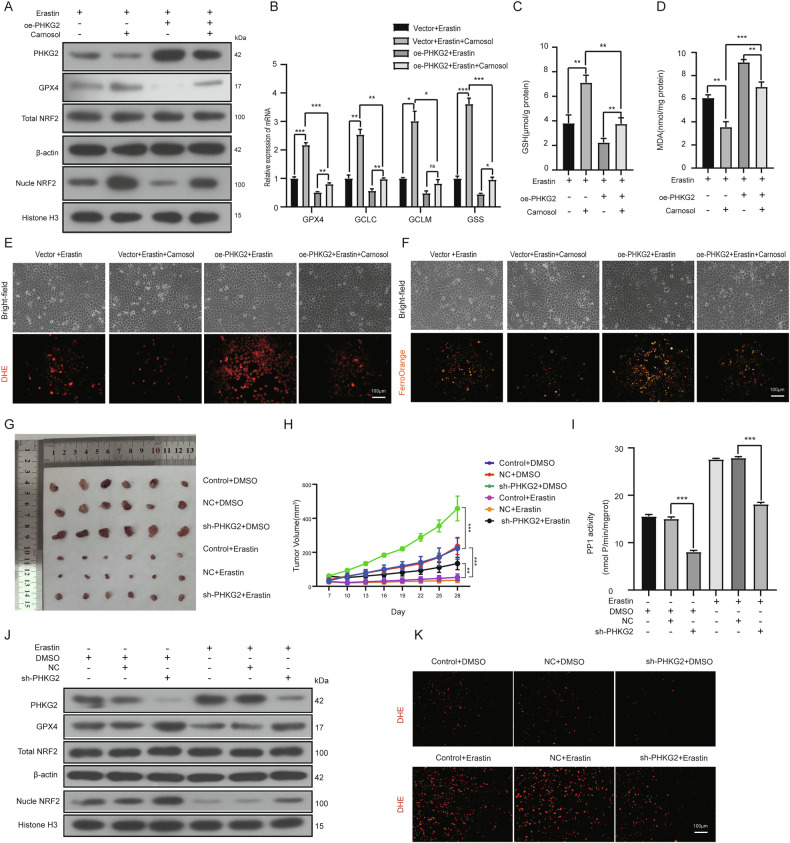
PHKG2 enhances ferroptosis sensitivity via the NRF2/GPX4 pathway in vitro and in vivo. Western blot analysis revealed that the NRF2 activator Carnosol increased nuclear NRF2 and GPX4 protein levels following Erastin treatment, whereas PHKG2 overexpression significantly reversed these increases (**A**). *qRT-PCR* analysis demonstrated that Carnosol treatment under ferroptotic stress elevated *GPX4, GSS, GCLC*, and *GCLM* mRNA levels, which were subsequently suppressed by *PHKG2* overexpression (*p < 0.05, **p < 0.01, ***p < 0.001) (**B**). Biochemical assays showed that PHKG2 overexpression antagonized Carnosol-induced elevation in GSH and reduction in MDA (**p < 0.01, ***p < 0.001) (**C**, **D**). Fluorescence assays for ROS and Fe^2+^ confirmed that PHKG2 overexpression restored ferroptotic sensitivity reduced by Carnosol treatment (**E**, **F**). In xenograft mouse models, PHKG2 knockdown impaired Erastin-induced tumor regression and decreased PP1 activation in tumors (**G**–**I**). Western blotting and ROS analysis revealed increased NRF2 and GPX4 expression and reduced ROS levels following PHKG2 knockdown (**J**, **K**).

Remarkably, *PHKG2* overexpression counteracted these protective effects. Under this conditions, it reduced nuclear NRF2, *GPX4 mRNA*, and GPX4 protein levels, lowered GSH content, suppressed *GCLC*, *GCLM*, and *GSS* mRNA expression, and restored MDA, ROS, and Fe^2+^ levels. (Fig. 5A–F), confirming that *PHKG2* can override NRF2-mediated ferroptosis resistance.

In vivo experiments yielded consistent results. Administration of Erastin significantly reduced tumor volume in xenografted mice, but this anti-tumor effect was attenuated when *PHKG2* was silenced (Fig. 5G, H). PP1 activity in tumor tissues was elevated in the Erastin-treated group, while *PHKG2* knockdown diminished this activation (Fig. 5I). At the molecular level, Western blotting revealed that interference with PHKG2 led to increased levels of GPX4 and nuclear NRF2. In contrast, Erastin alone suppressed their nuclear expression, and the combined effect of Erastin plus *PHKG2* knockdown partially restored these protein levels (Fig. 5J). Fluorescence imaging for ROS further supported these findings: ROS levels were elevated in Erastin-treated tumors, but significantly reduced upon *PHKG2* knockdown (Fig. 5K).

Together, these results demonstrate that *PHKG2* promotes ferroptosis by repressing the NRF2/GPX4 antioxidant pathway and is capable of restoring ferroptotic sensitivity even under NRF2-activating conditions.

### PHKG2 facilitates NRF2 nuclear export by phosphorylating PPP1R3B and disrupting the PPP1R3B–PP1C complex

**Fig. 6:**
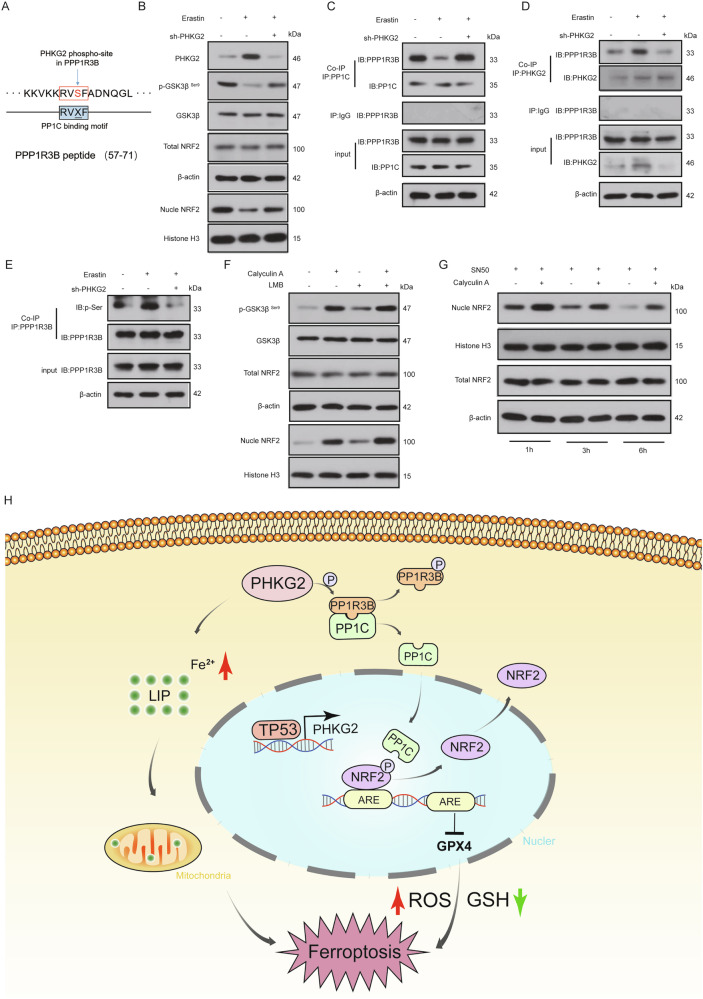
PHKG2 promotes NRF2 nuclear export by disrupting PPP1R3B–PP1C interactions. Phosphorylation prediction analysis indicated PHKG2 phosphorylation of PPP1R3B at Ser64 within the RVXF motif, essential for interaction with PP1C (**A**). Western blotting showed that PHKG2 knockdown elevated p-GSK3β (Ser9) and nuclear NRF2 levels, without altering total GSK3β or NRF2 protein levels (**B**). Co-IP assays confirmed direct interactions of PPP1R3B with PP1C and PHKG2, influenced by PHKG2 expression levels (**C**, **D**). Immunoprecipitation revealed reduced PPP1R3B phosphorylation upon PHKG2 knockdown (**E**). Western blot experiments showed that the PP1 inhibitor Calyculin A and the nuclear export inhibitor Leptomycin B (LMB) increased nuclear NRF2 accumulation, whereas co-treatment showed no further synergism, suggesting PP1 primarily promotes NRF2 nuclear export (**F**). Additionally, nuclear import inhibition by SN50 failed to fully prevent NRF2 nuclear accumulation induced by Calyculin A, further supporting PP1’s role in nuclear export (**G**). The schematic illustrates the proposed mechanism: TP53-mediated PHKG2 activation triggers PPP1R3B phosphorylation, disrupting PPP1R3B–PP1C interactions, thereby enhancing PP1 activity, reducing nuclear NRF2 and GPX4 levels, decreasing intracellular GSH, and elevating ROS and Fe^2+^, ultimately promoting ferroptosis (**p < 0.01, ***p < 0.001) (**H**).

To elucidate how PHKG2 modulates PP1 activity at the molecular level, we utilized phosphorylation site prediction analysis. The results identified Ser64 within the RVXF motif of PPP1R3B—a known regulatory subunit of PP1C—as a potential phosphorylation target of PHKG2 (Figs. 6A and S2D). Phosphorylation at this site is known to weaken the interaction between PPP1R3B and PP1C, thereby enhancing the phosphatase activity of PP1 [25, 26].

Experimental validation supported this mechanism. PHKG2 knockdown increased the level of phosphorylated GSK3β at Ser9 and led to marked nuclear accumulation of NRF2, while total GSK3β and NRF2 expression remained unchanged (Fig. 6B). Co-immunoprecipitation (Co-IP) assays confirmed physical interactions between PPP1R3B and both PP1C and PHKG2 (Fig. 6C, D). Furthermore, immunoprecipitation analysis revealed that suppression of PHKG2 reduced PPP1R3B phosphorylation (Fig. 6E), consistent with the proposed model of PHKG2 acting upstream to modulate PP1 activity through PPP1R3B phosphorylation.

To dissect the functional consequence of PP1 regulation on NRF2 localization, we applied pharmacological inhibitors. Both the PP1 inhibitor Calyculin A and the nuclear export blocker Leptomycin B (LMB) led to increased p-GSK3β (Ser9) and enhanced nuclear retention of NRF2 (Fig. 6F). Notably, co-treatment with both agents did not show additive effects, suggesting that PP1 and nuclear export operate within the same mechanistic pathway.

Further mechanistic confirmation came from experiments using SN50, a nuclear import inhibitor. Even with SN50-mediated inhibition of nuclear import, Calyculin A treatment still resulted in NRF2 nuclear accumulation (Fig. 6G), reinforcing the idea that PP1 primarily regulates NRF2 localization through nuclear export rather than import.

Taken together, these findings establish a detailed mechanistic model: PHKG2 phosphorylates PPP1R3B, thereby disrupting its interaction with PP1C and enhancing PP1 phosphatase activity. The activated PP1 promotes NRF2 dephosphorylation and nuclear export, leading to downregulation of downstream antioxidant genes such as GPX4. The resultant decrease in GSH and increase in ROS and Fe^2+^ collectively sensitize HNSCC cells to ferroptosis (Fig. 6H).

## Discussion

Our study identifies PHKG2 as a ferroptosis-related gene with prognostic relevance in HNSCC. Clinically, PHKG2 expression correlates inversely with tumor stage and serves as an independent protective factor. Mechanistically, we propose a novel regulatory model: transcriptional activation of PHKG2 by *TP53* leads to enhanced phosphorylation of PPP1R3B, which weakens its interaction with the catalytic subunit PP1C. This disruption facilitates PP1C activation, promoting NRF2 dephosphorylation and nuclear export. The resulting downregulation of the NRF2/GPX4 axis compromises the antioxidant defense system and triggers ferroptotic cell death (Fig. 6H). Our identification of the *TP53*–PHKG2–PP1–NRF2 signaling cascade reveals a previously uncharacterized ferroptosis regulatory mechanism in HNSCC, highlighting redox imbalance as a critical vulnerability for therapeutic exploitation and paving the way for ferroptosis-based therapeutic interventions in redox-adapted tumors.

Previous work has shown that PHKG2 enhances sensitivity to Erastin-induced ferroptosis, and its function appears linked to *TP53* and intracellular iron pools [21]. However, the precise mechanism of this interaction had not been delineated. *TP53*, often referred to as the “guardian of the genome,” is known to regulate ferroptosis independently of apoptosis, senescence, and cell cycle arrest, particularly under oxidative stress [27]. Our findings provide compelling evidence that *TP53* directly binds to and activates the PHKG2 promoter, forming a unidirectional regulatory cascade. Interestingly, we also observed that PHKG2 overexpression leads to increased *TP53* expression in vivo, suggesting a potential positive feedback loop, although this reverse regulation may involve indirect or stress-mediated mechanisms. Notably, *TP53* suppression only partially abrogated PHKG2-induced *TP53* expression, implying both *TP53*-dependent and -independent modes of feedback activation.

Such feedback loops are uncommon but not unprecedented within the *TP53* regulatory network. *TP53* plays dual roles in redox homeostasis [28]: under moderate oxidative stress, it mitigates ROS accumulation and promotes survival, whereas under excessive ROS conditions, it enhances lipid peroxidation and ferroptosis to eliminate damaged cells [27, 29]. Moreover, this *TP53*-driven ferroptotic mechanism operates independently of canonical tumor-suppressive programs such as cell cycle arrest, apoptosis, or senescence [27]. The *TP53*–PHKG2 axis identified in our study may thus act as a reinforcing circuit that amplifies ferroptosis signaling specifically under conditions of malignant oxidative stress.

At the biochemical level, PHKG2 encodes the catalytic subunit of phosphorylase kinase (PHK), which activates glycogen phosphorylase (GP) to mobilize glucose-1-phosphate from glycogen reserves [30]. Although Yang et al. hypothesized that the PHK–GP–glycogen axis mediates ferroptotic sensitivity, their findings refuted this metabolic explanation, implicating an alternate, noncanonical role of PHKG2 in tumor ferroptosis [21]. Our study identifies PPP1R3B as a direct phosphorylation substrate of PHKG2, which, upon modification, disrupts its interaction with PP1C and unleashes phosphatase activity. This cascade ultimately drives NRF2 dephosphorylation and nuclear export, impairing its transcriptional control over key antioxidant genes, notably GPX4. Given GPX4’s role in detoxifying lipid hydroperoxides, its repression via PHKG2 activity shifts the redox balance toward ferroptosis. Collectively, these findings highlight PHKG2 as a regulatory “switch” that tilts tumor cells toward ferroptotic death by inactivating the NRF2/GPX4 axis. Importantly, this work underscores the functional plasticity of metabolic enzymes in cancer biology—PHKG2, a classical glycogen-related kinase, is reprogrammed here to regulate stress-responsive transcription networks.

A large number of ferroptosis-regulating genes are known to be direct transcriptional targets of NRF2, underscoring its central role in ferroptosis suppression [31–33]. Under normal physiological conditions, NRF2 is sequestered in the cytoplasm by KEAP1, rendering it inactive. Upon oxidative stress, conformational changes in KEAP1 lead to its dissociation from NRF2, which undergoes phosphorylation and translocates into the nucleus. There, it binds to antioxidant response elements (AREs) [34] and activates transcription of downstream antioxidant genes, including GPX4, SLC7A11, and members of the GST family [32, 35–37].

Elevated NRF2 expression has been widely associated with poor prognosis in a variety of cancers [38–41], and mounting evidence points to its crucial role in conferring resistance to ferroptosis-based therapies in HNSCC, particularly in the context of chemotherapy and radiotherapy [42]. Functionally, NRF2 acts as a key metabolic switch that enables tumor cells to adapt to oxidative stress by upregulating redox-balancing pathways, thereby supporting their proliferative demands and survival under stress [43–45]. Clinically, NRF2-overexpressing tumors often display resistance to conventional chemoradiotherapies [46, 47]. In this context, our findings that *TP53*/PHKG2 signaling attenuates nuclear NRF2 protein levels and impairs its downstream transcriptional activity may provide a mechanistic explanation for increased ferroptotic vulnerability in PHKG2-high tumors.

The NRF2/GPX4 axis represents a pivotal regulatory hub for ferroptosis resistance and oxidative detoxification. Activation of this pathway allows tumor cells to neutralize lipid peroxides generated during rapid proliferation, thereby preventing ferroptotic collapse [43]. However, this also renders them more aggressive and refractory to treatment, correlating with unfavorable clinical outcomes [48]. Indeed, numerous studies have shown that disrupting NRF2 or GPX4 signaling can restore ferroptotic sensitivity in resistant tumor cells. In hepatocellular carcinoma models, suppression of the p62–KEAP1–NRF2 pathway markedly enhances the efficacy of ferroptosis inducers such as Erastin and sorafenib [49]. Among NRF2 targets, GPX4 is uniquely positioned as the only enzyme capable of directly reducing complex phospholipid hydroperoxides [50], making it a critical gatekeeper of ferroptosis [51]. Wang et al. further demonstrated that upregulation of NRF2 and ferroptosis-related genes such as GPX4, SLC7A11, and FTL can mitigate sorafenib-induced ferroptosis in liver cancer cells [50]. In HNSCC, activation of the NRF2–ARE pathway has been shown to protect cells from GPX4 inhibition, while blockade of this pathway restores susceptibility to ferroptosis [52].

Our data position PHKG2 as a promising therapeutic target in HNSCC, particularly due to its antagonistic relationship with NRF2. Supporting this, a recent study in lung cancer revealed that radiosensitive tumors exhibited high PHKG2 and low NRF2 expression, whereas radioresistant tumors displayed the inverse profile. Mechanistically, the authors identified an NRF2–RPA1 complex that transcriptionally suppresses PHKG2, while PHKG2, in turn, enhances radiosensitivity by impairing mitochondrial function [53]. In line with these findings, we observed a negative correlation between PHKG2 and NRF2 expression in HNSCC samples, with higher PHKG2 levels associated with earlier tumor stage and better clinical outcomes. Mechanistically, PHKG2 suppresses NRF2 function by phosphorylating PPP1R3B, thereby releasing PP1C and promoting NRF2 nuclear export. This PHKG2–NRF2 interaction forms a potential positive feedback loop that amplifies ferroptosis sensitivity.

Beyond its redox-regulatory role, PHKG2 has emerged as a broader ferroptosis-associated gene and favorable prognostic marker in multiple cancer types [19, 21, 54]. Interestingly, PHKG2 expression has been shown to negatively correlate with immune checkpoint molecule expression and immune cell infiltration [54]. These findings suggest that tumors with high PHKG2 expression may not only be more ferroptosis-sensitive, but also more immunogenic. Given that ferroptosis can enhance tumor antigenicity and immune cell recruitment, therapeutic combinations involving ferroptosis inducers and immune checkpoint inhibitors (e.g., anti-PD-1/PD-L1 antibodies) may represent a rational next step for clinical development.

Despite these insights, our study has several limitations. First, mechanistic conclusions are largely derived from cell line and xenograft models; large-scale clinical validation is needed to establish PHKG2 as a prognostic biomarker. Second, the differential behavior of the *TP53*–PHKG2–NRF2 axis in HPV-positive versus HPV-negative HNSCC subtypes remains to be clarified. Third, the exact PP1C dephosphorylation sites on NRF2 remain undefined and warrant further investigation using phosphoproteomics. Moreover, since *TP53* mutations are common in HNSCC, future studies should explore whether PHKG2 can be activated through alternative, *TP53*-independent pathways or rescued via gene therapy.

In summary, our findings uncover a novel ferroptosis regulatory mechanism in HNSCC driven by the *TP53*–PHKG2–PP1–NRF2 axis. PHKG2 emerges as both a modulator of redox homeostasis and a potential therapeutic target, offering new opportunities for ferroptosis-based treatment strategies, either alone or in combination with immunotherapy.

## Supplementary information

Supplement Figure Legend

Supplement File

Supplementary Western Blot

Supplemental Figure 1

Supplemental Figure 2

## Data Availability

The datasets analyzed in this study were obtained from The Cancer Genome Atlas (TCGA, https://portal.gdc.cancer.gov/) on September 26, 2020. The data are publicly available without restrictions.
