# Supplementary material for: Transcriptional activation of PHKG2 by TP53 promotes ferroptosis through nuclear export of NRF2 in head and neck squamous cell carcinoma
Source: Cell Death Dis. 2025 Aug 30;16(1):662. doi: 10.1038/s41419-025-07985-3 (PMC12398534; doi:10.1038/s41419-025-07985-3)
Supplement: Supplementary file 1 — Supplement Figure Legend [file 41419_2025_7985_MOESM1_ESM.docx]

**Fig. 1S** PHKG2 enhances ferroptosis via ROS and Fe²⁺ accumulation in HNSCC cells. qRT-PCR screening identified sh-PHKG2-419 as the most effective interference sequence **(A)**. DHE staining of 5-8F **(B–C)** and Fadu **(D–E)** cells under ferroptosis activation (RSL3 or FIN56) showed that PHKG2 overexpression increased lipid ROS levels, whereas PHKG2 knockdown reduced ROS accumulation compared with controls. FerroOrange staining of 5-8F **(F–G)** and Fadu **(H–I)** cells under ferroptosis activation (RSL3 or FIN56) revealed that intracellular Fe²⁺ levels were elevated in PHKG2-overexpressing cells and reduced in PHKG2-silenced cells relative to controls.

**Fig. 2S** TP53 directly targets PHKG2, and PHKG2 modulates PP1 via PPP1R3B phosphorylation. ChIP assays confirmed the direct binding of TP53 to the PHKG2 promoter **(A-B)**. iGSP1.0 analysis predicted potential PHKG2-mediated phosphorylation sites on PP1 **(C)**. PHKG2 was predicted to phosphorylate Ser64 within the RVXF motif of the PP1 regulatory subunit PPP1R3B **(D)**.
