# Supplementary material for: Transcriptional activation of PHKG2 by TP53 promotes ferroptosis through nuclear export of NRF2 in head and neck squamous cell carcinoma
Source: Cell Death Dis. 2025 Aug 30;16(1):662. doi: 10.1038/s41419-025-07985-3 (PMC12398534; doi:10.1038/s41419-025-07985-3)
Supplement: Supplementary file 2 — Supplement File [file 41419_2025_7985_MOESM2_ESM.docx]

1. siRNA sequences and qPCR primer sequences

Primer sequences (5′-3′) are as follows:

PHKG2 Forward: 5’- CCACACTCAACATTTCGGGG -3’,

PHKG2 Reverse: 5’- CTGGCGAAGGATGTGTGTCTC -3’,

NRF2 Forward: 5’-CCACACTCAACATTTCGGGG-3’,

NRF2 Reverse: 5’- GCAGATTGCACTGATGCTGG-3’,

TP53 Forward: 5’-TACCACCATCCACTACAAC-3’,

TP53 Reverse: 5’-ACACGCACCTCAAAGC-3’,

GPX4 Forward: 5’- GCCTTTGCCGCCTACTGA-3’,

GPX4 Reverse: 5’- TGTGCCCGTCGATGTCCT-3’

GCLC Forward: 5’- TCATTTCCCAGATTAGGC-3’

GCLC Reverse: 5’- AGGGTGCTTGTTTATTGC-3’

GCLM Forward: 5’- GCACAGCGAGGAGCTTCAT-3’

GCLM Reverse: 5’- ATCCAGCTGTGCAACTCCAA-3’

GSS Forward: 5’- CTGAGGGAGTATTGCTGA-3’

GSS Reverse: 5’- TGGTGCTGGAAAGAGTT-3’

GAPDH Forward: 5’- CCCTTCTGCCTGACACC -3’,

GAPDH Reverse: 5’- AAGCAGTTGGTGGTGCAG -3’

β-actin Forward: 5’- GGCACCCAGCACAATGAA -3’,

β-actin Reverse: 5’- TAGAAGCATTTGCGGTGG -3’

Si-PHKG2 419 Forward: 5’- AGGGAGAGCUGUUUGACUATT -3’,

Reverse: 5’- UAGUCAAACAGCUCUCCCUTT -3’,

Si-PHKG2 236 Forward: 5’- CGGUGAAGAUUAUGGAAGUTT -3’,

Reverse: 5’- ACUUCCAUAAUCUUCACCETT -3’,

Si-PHKG2 134 Forward: 5’- CCAAAGAGUUUUACCAGAATT -3’,

Reverse: 5’- UUCUGGUAAAACUCUUUGGTT -3’,

Si-NC Forward: 5’- UUCUCCTGAACGUGUCACGUTT -3’,

Reverse: 5’- ACGUGACACGUUCGGAGAATT -3’.

2. Ferroptosis-related genes used in bioinformatics analysis

ACSL4, AKR1C1, AKR1C2, AKR1C3, ALOX15, ALOX5, ALOX12, ATP5MC3, CARS1, CBS, CD44, CHAC1, CISD1, CS, DPP4, FANCD2, GCLC, GCLM, GLS2, GPX4, GSS, HMGCR, HSPB1, CRYAB, LPCAT3, MT1G, NCOA4, PTGS2, RPL8, SAT1, SLC7A11, FDFT1, TFRC, TP53, EMC2, AIFM2, PHKG2, HSBP1, ACO1, FTH1, STEAP3, NFS1, ACSL3, ACACA, PEBP1, ZEB1, SQLE, FADS2, NFE2L2, KEAP1, NQO1, NOX1, ABCC1, SLC1A5, GOT1, G6PD, PGD, IREB2, HMOX1, ACSF2

1. Gene coefficients in Lasso model

CARS1 0.0124473792864562

CD44 0.00656892634519636

CHAC1 0.0844227080273681

CS -0.00449829566378141

TFRC 0.00589623445467654

PHKG2 -0.13421657500621

HSBP1 0.0530314661477438

FTH1 0.00238657317126794

FADS2 -0.0147272219366787

ABCC1 -0.00400621074731041

GOT1 -0.00778826043687221
