# Supplementary material for: Transcriptional activation of PHKG2 by TP53 promotes ferroptosis through nuclear export of NRF2 in head and neck squamous cell carcinoma
Source: Cell Death Dis. 2025 Aug 30;16(1):662. doi: 10.1038/s41419-025-07985-3 (PMC12398534; doi:10.1038/s41419-025-07985-3)

Fig.3 B TP53

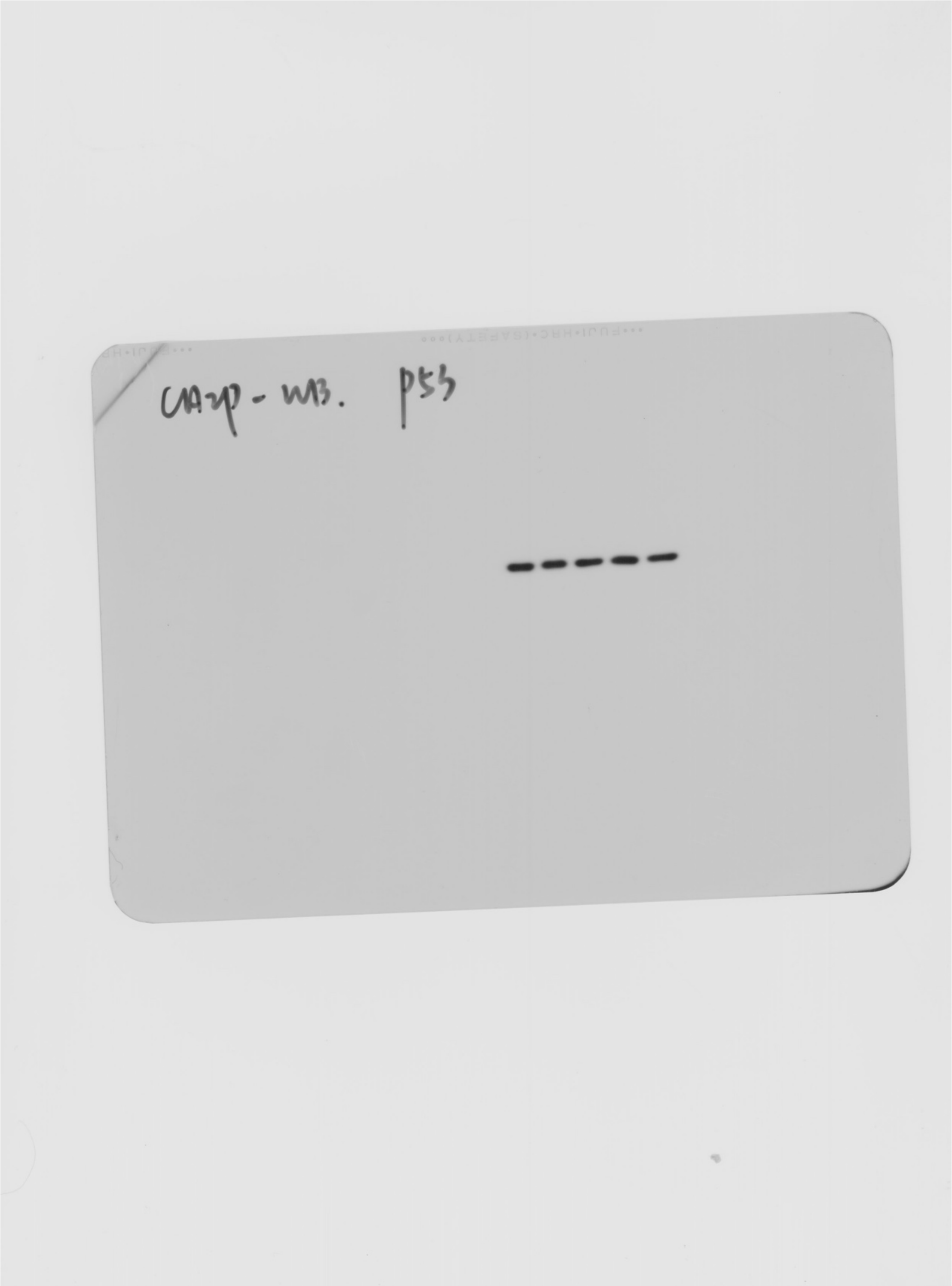

Fig3 F TP53

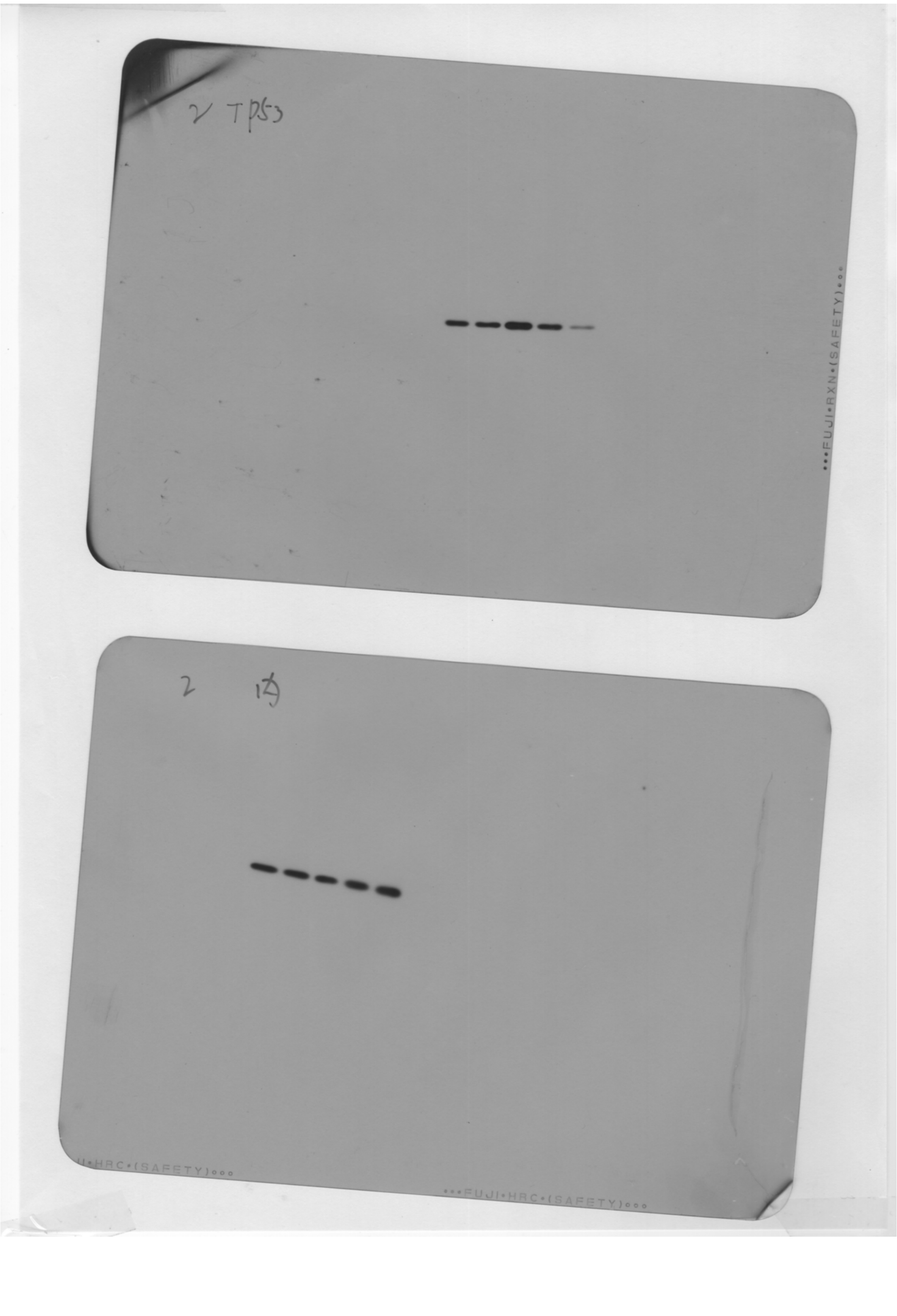

Fig3 F-Nuclear-NRF2

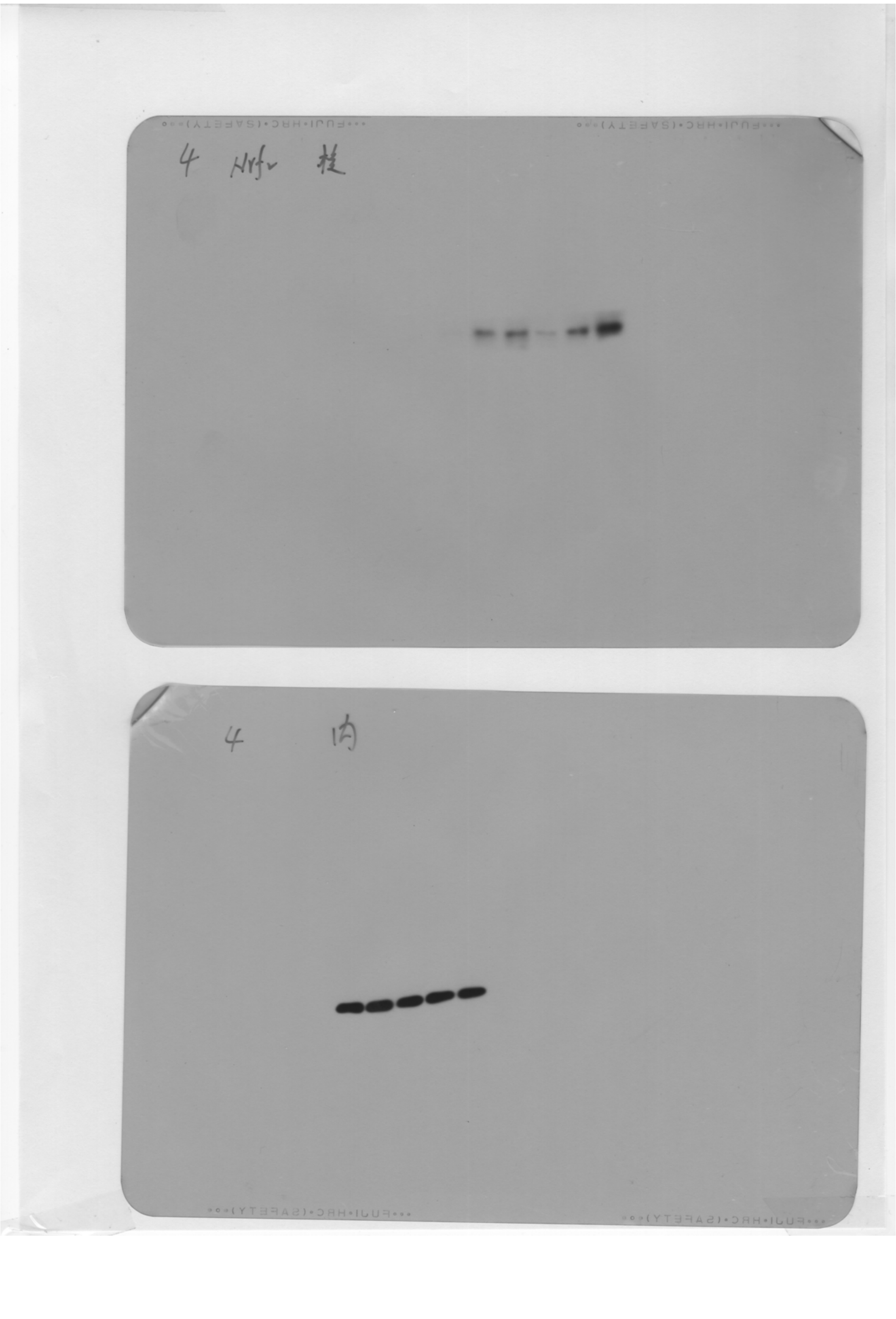

Fig3 F-NRF2 Total

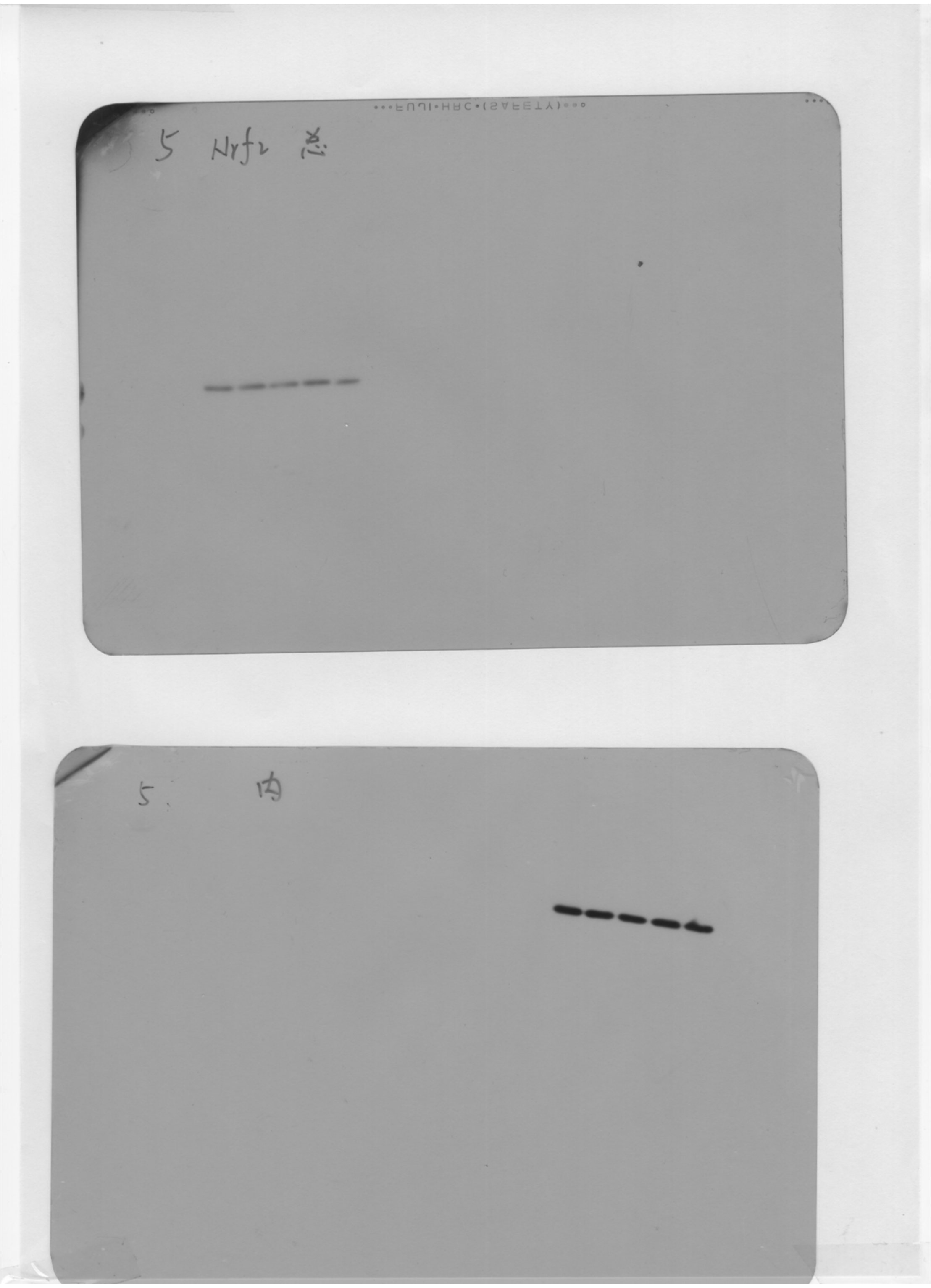

Fig3 N-TP53

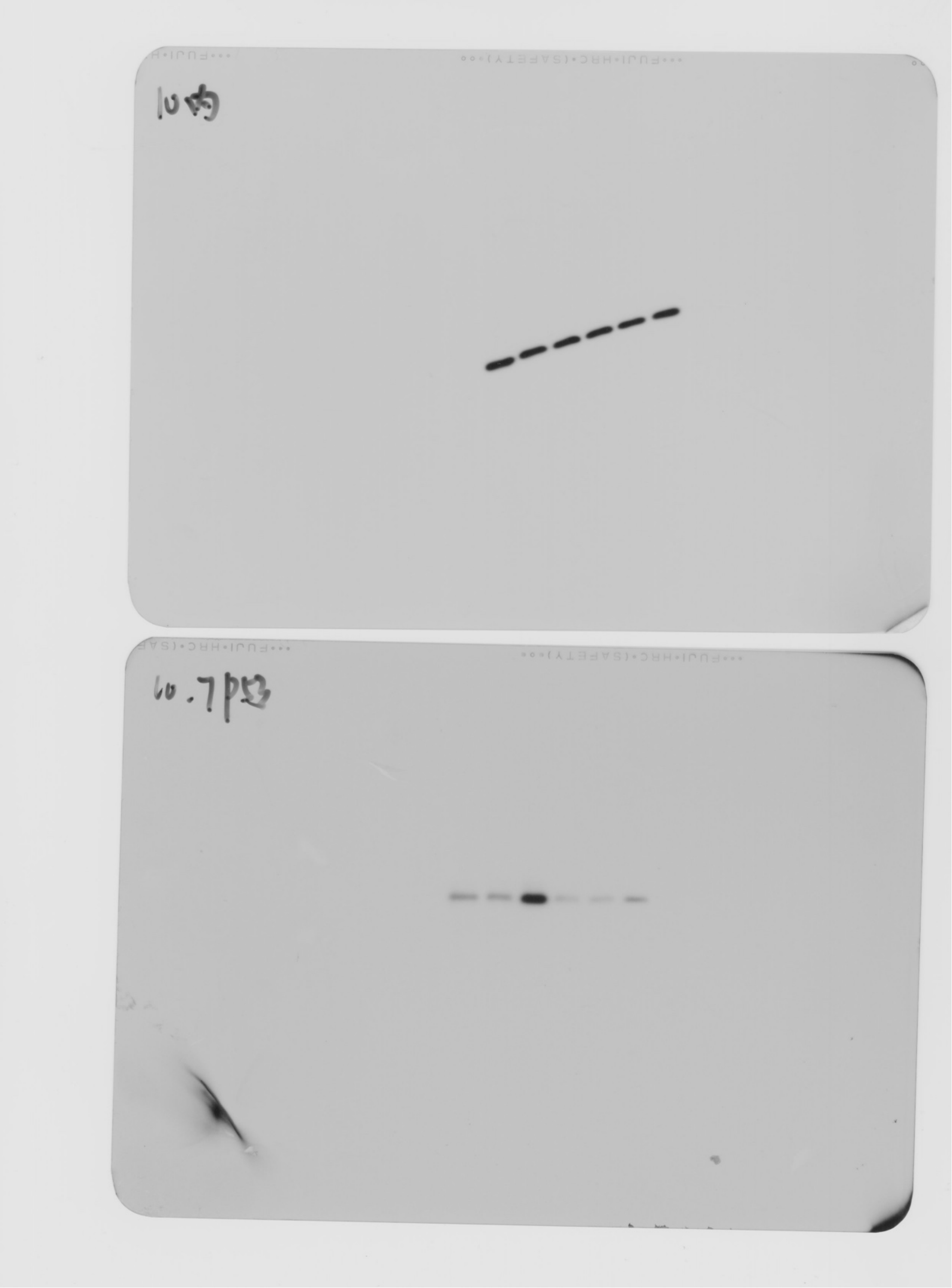

Fig3 N-PHKG2

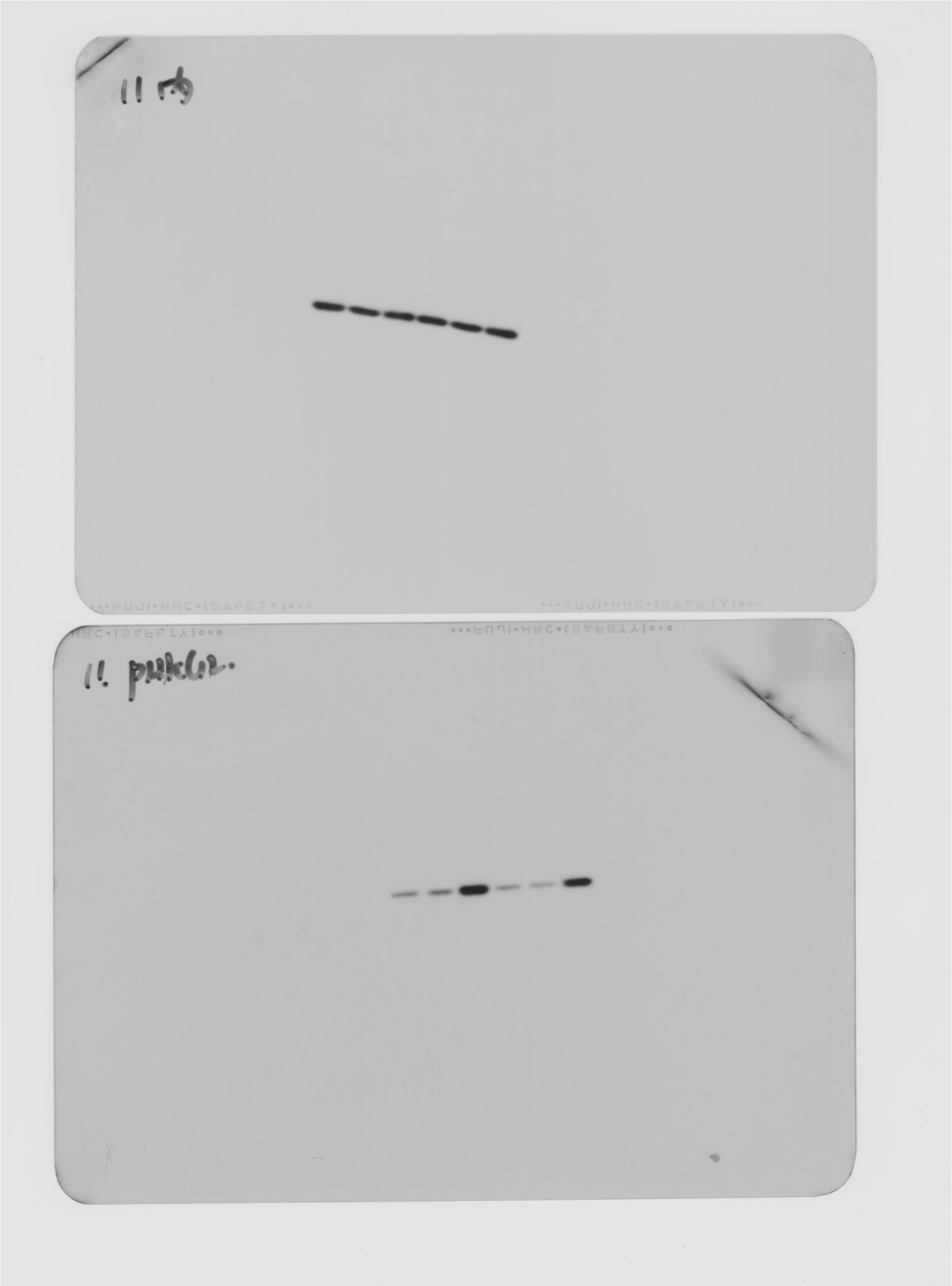

Fig.3-N-nucle NRF2

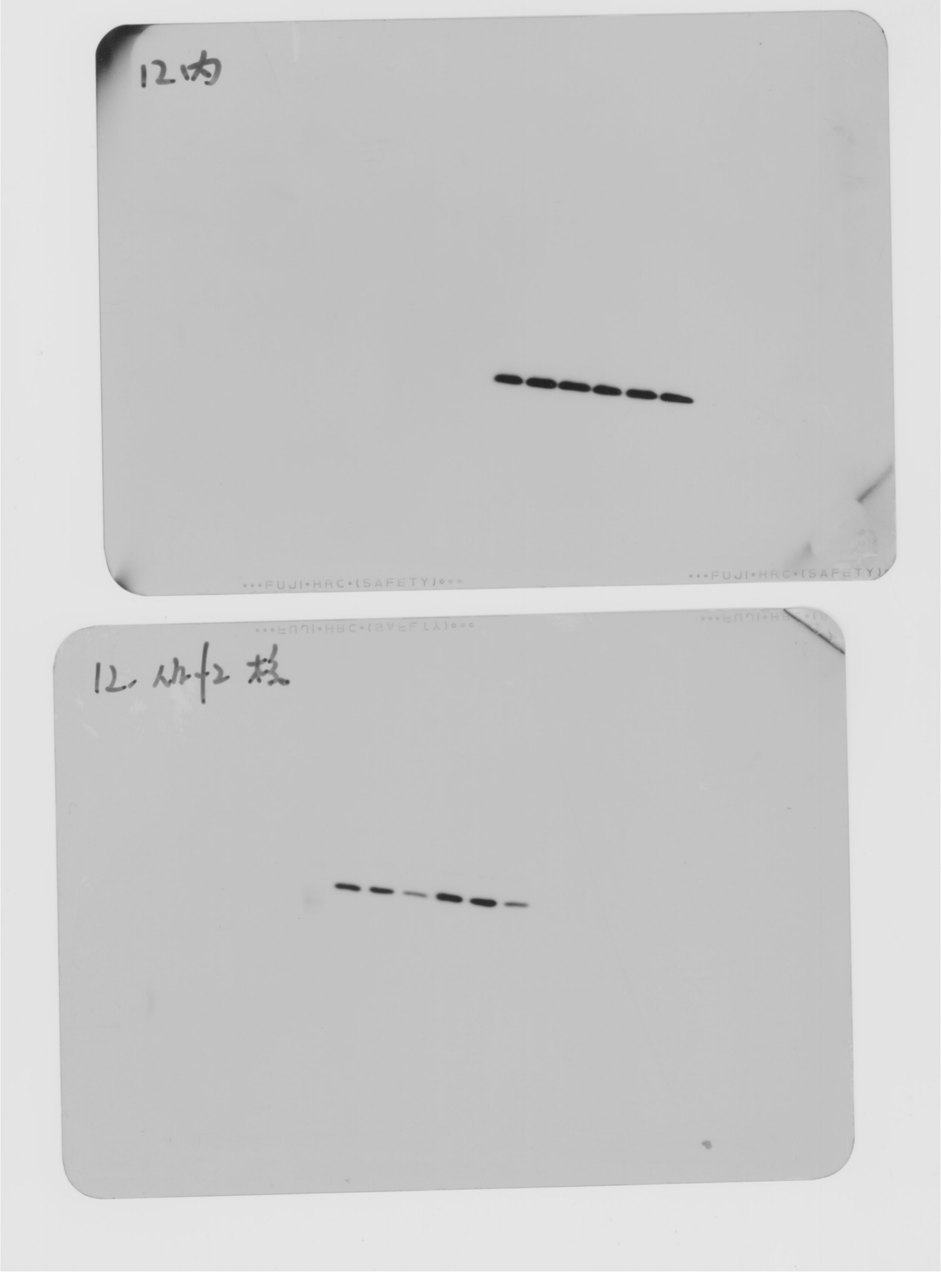

Fig.3-N-total NRF2

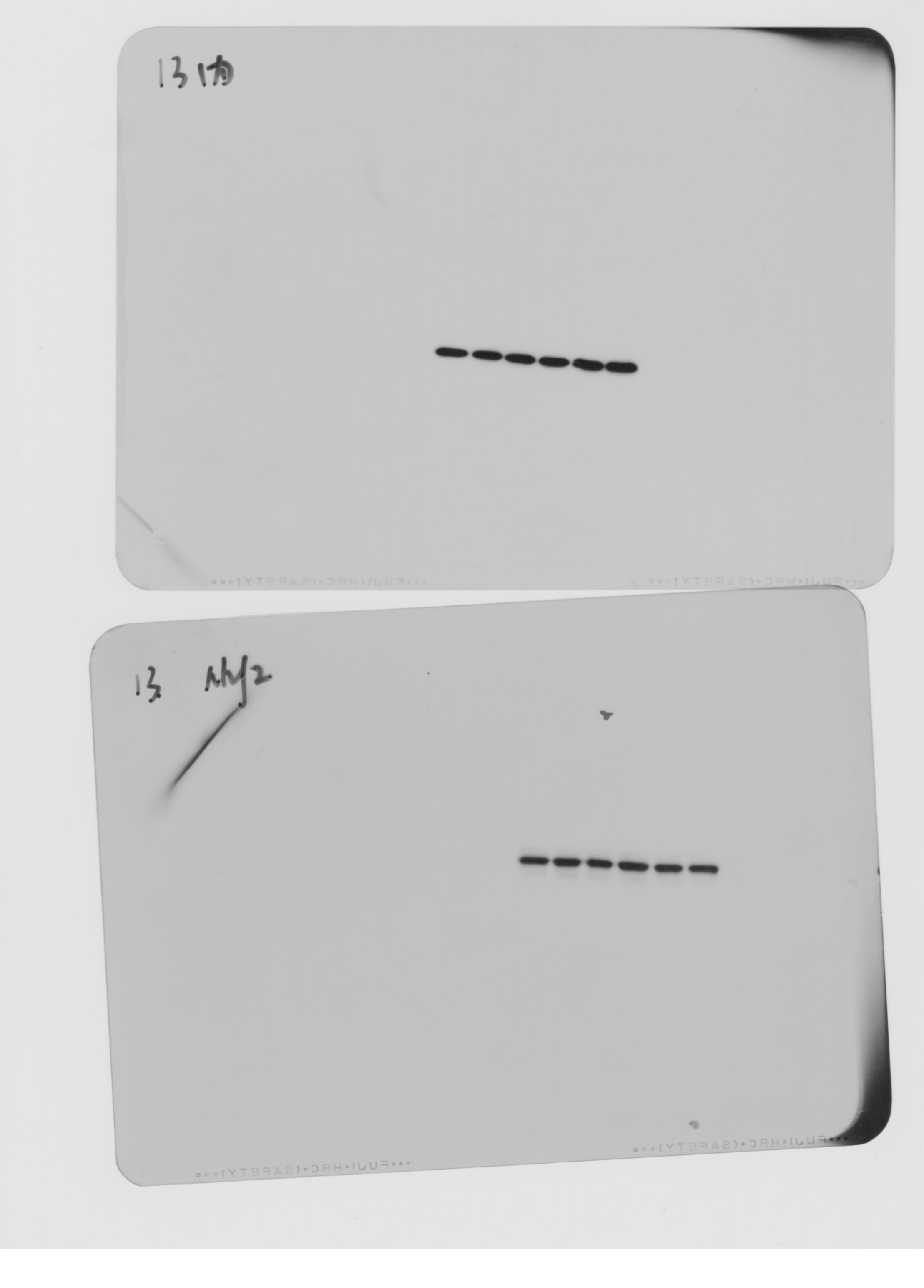

Fig.4 B+C Nuclear NRF2

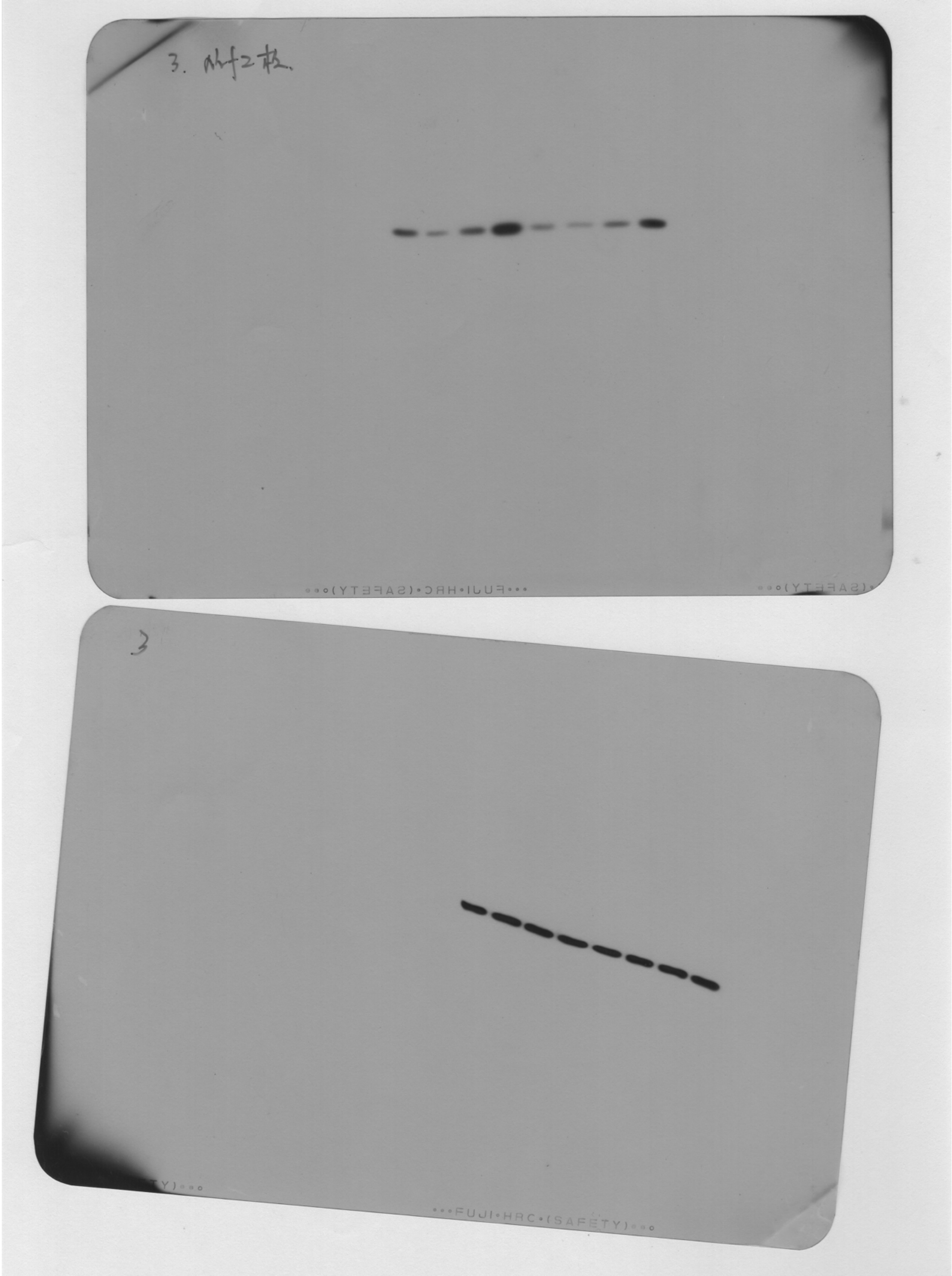

Fig.4 B GPX4 少 C 的 GPX4

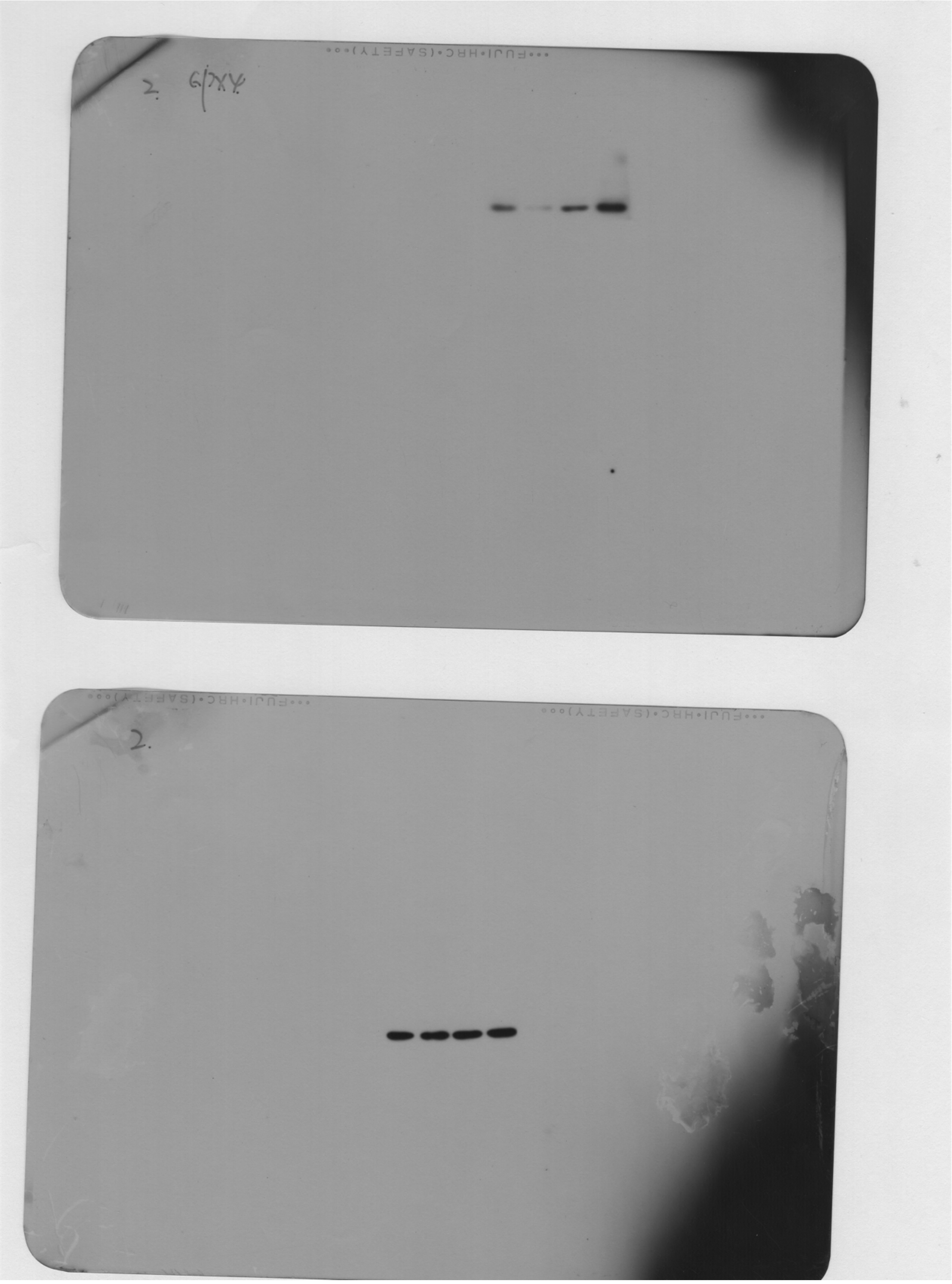

Fig.4 B+C　Total NRF2

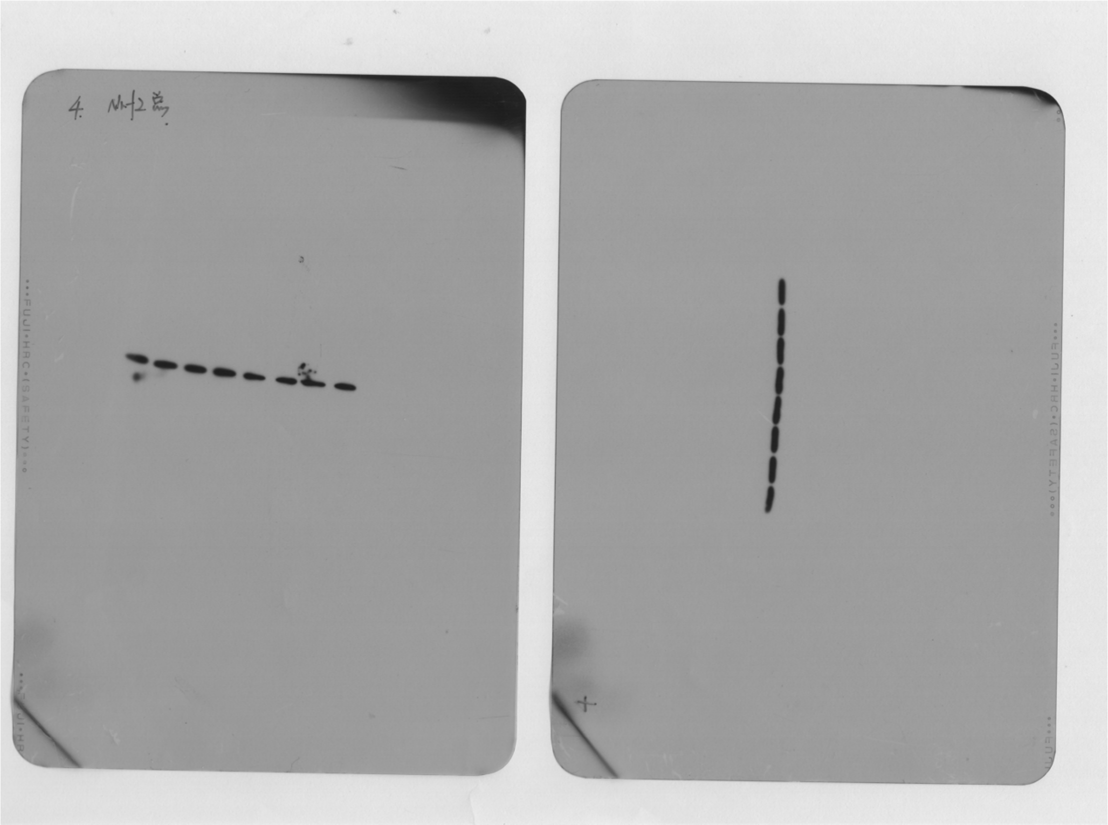

Fig.4 F+G PHKG 2

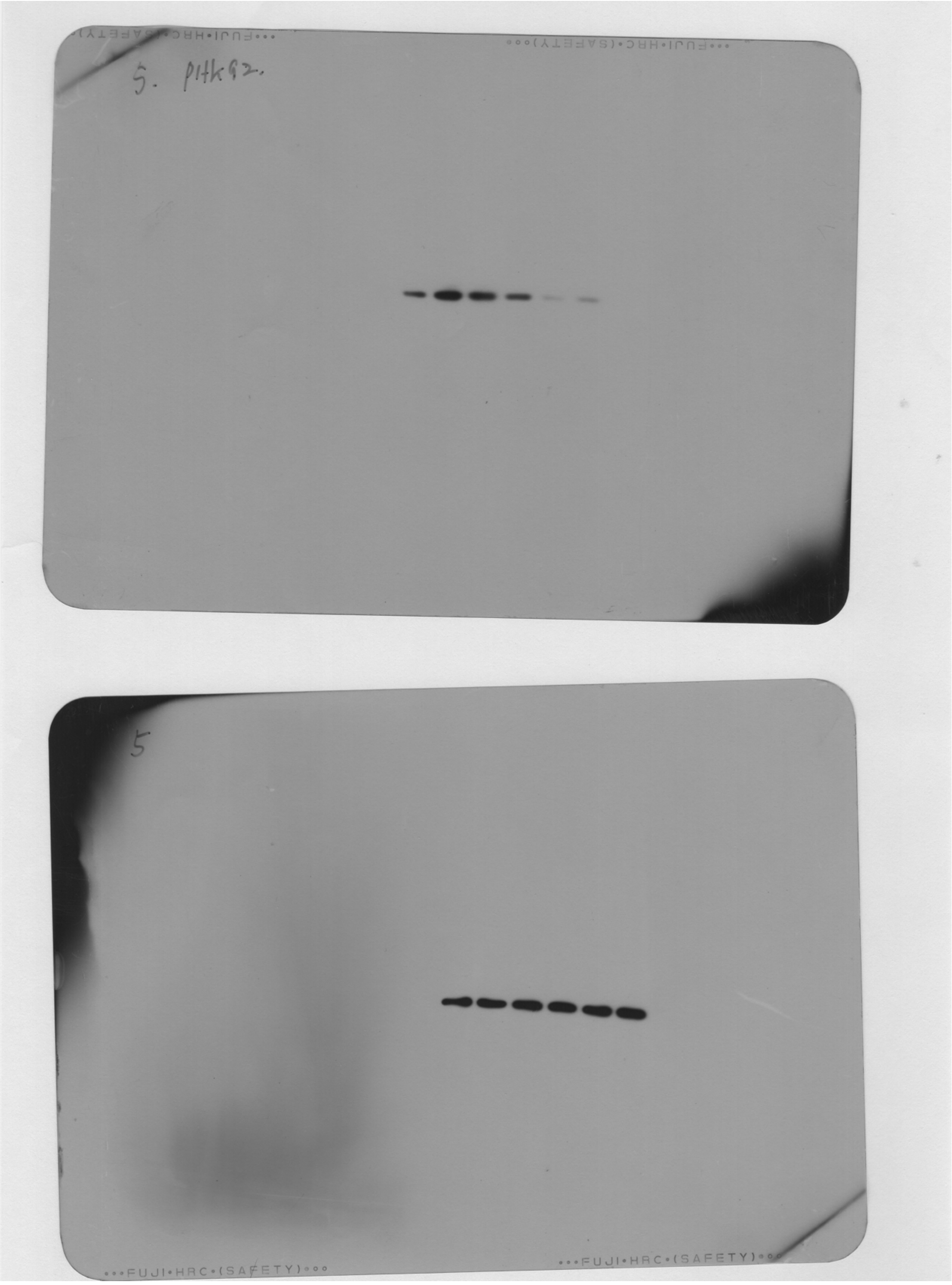

Fig.4 F+G Total NRF2

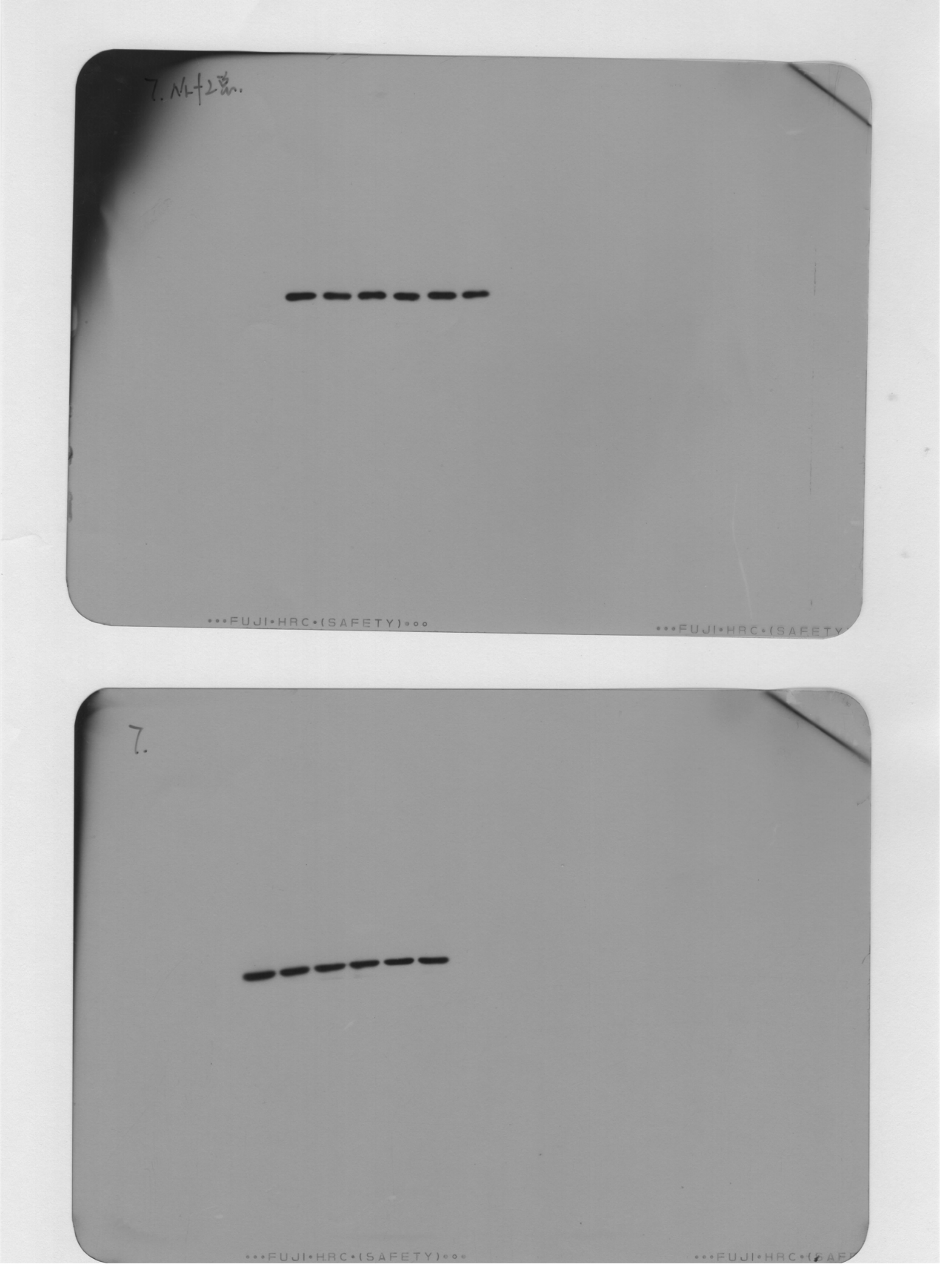

Fig.4 F+G Nuclear NRf2

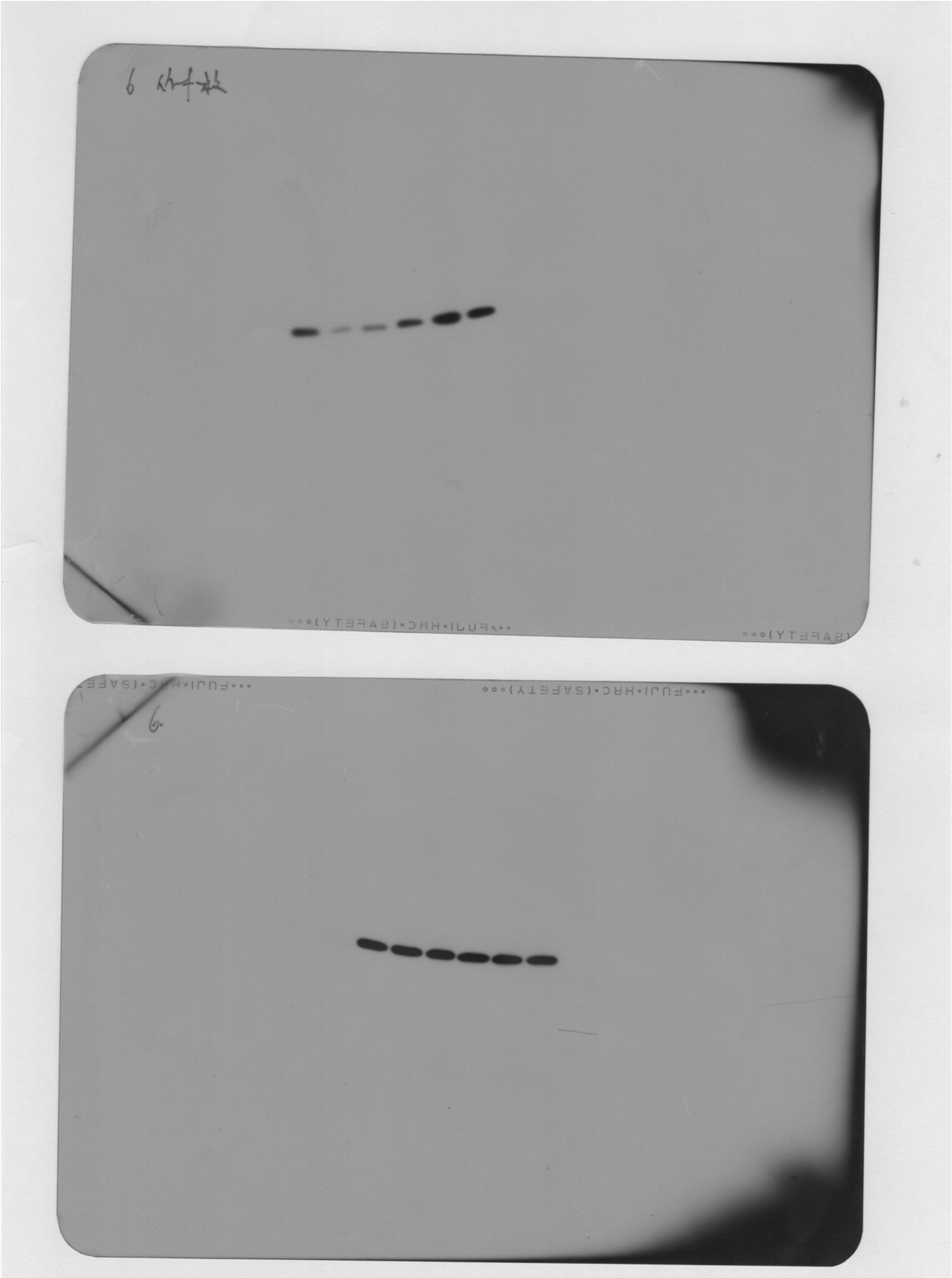

Fig5. A nuclear NRF2

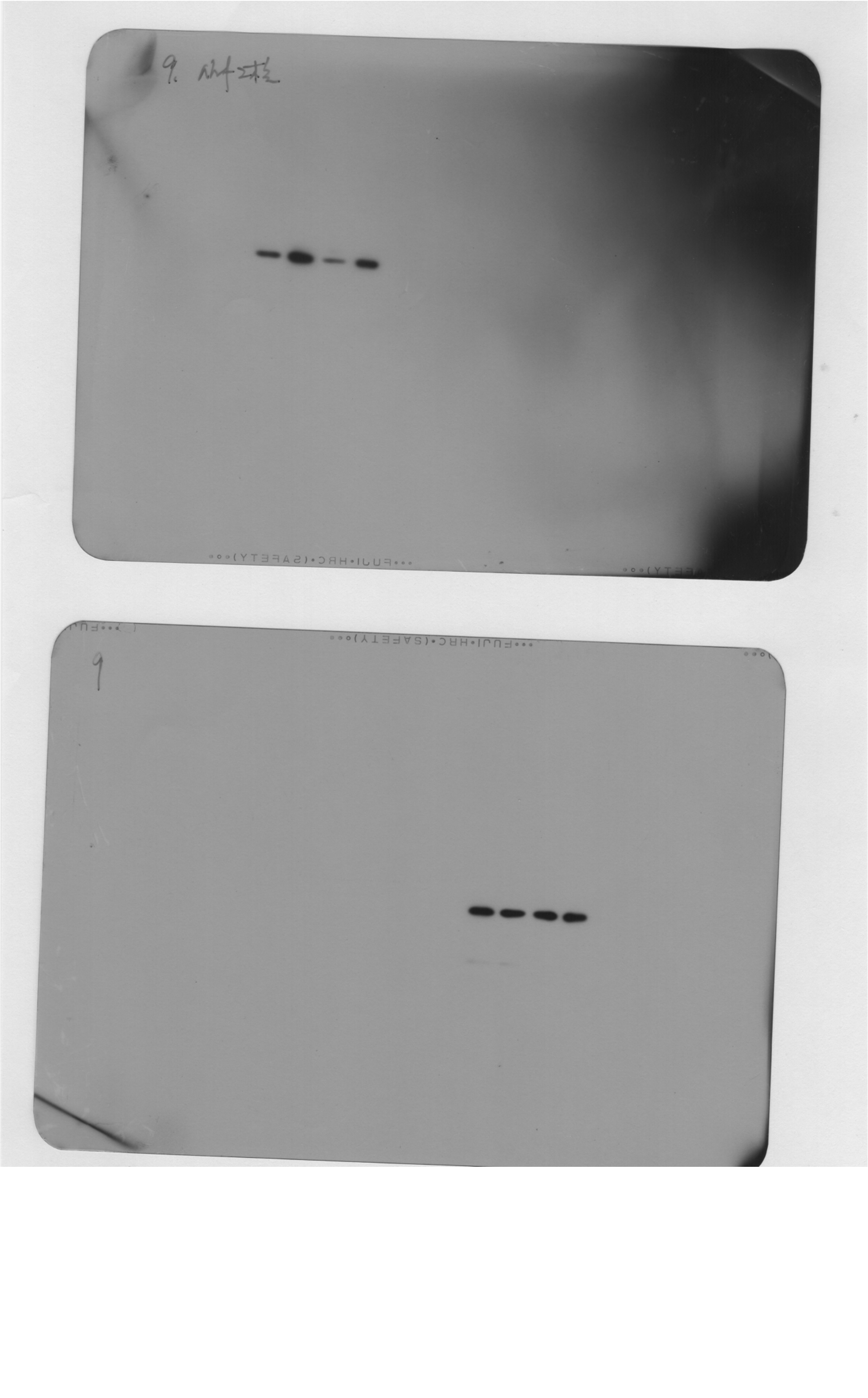

Fig5.A PHKG2

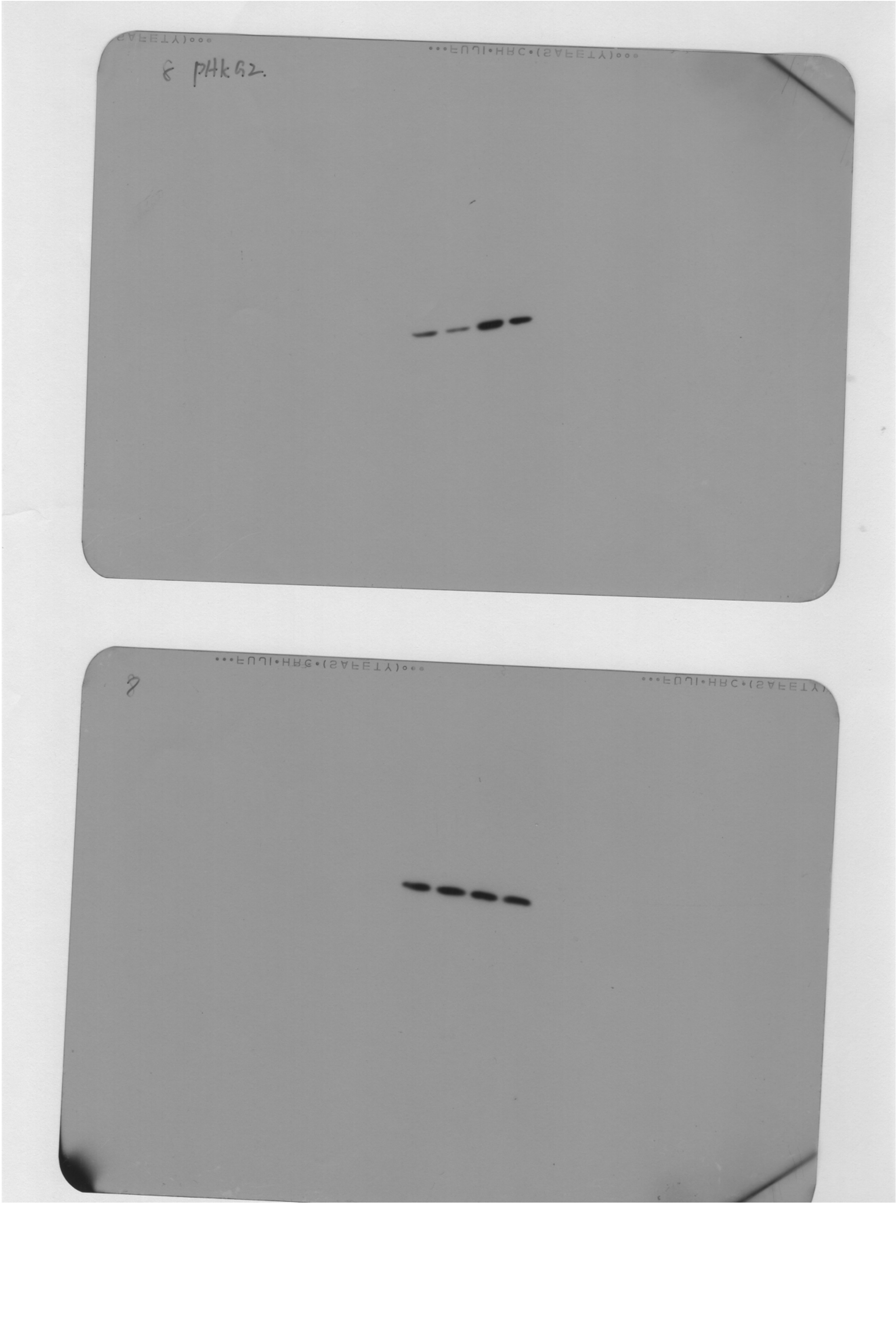

Fig5.A Total-NRF2

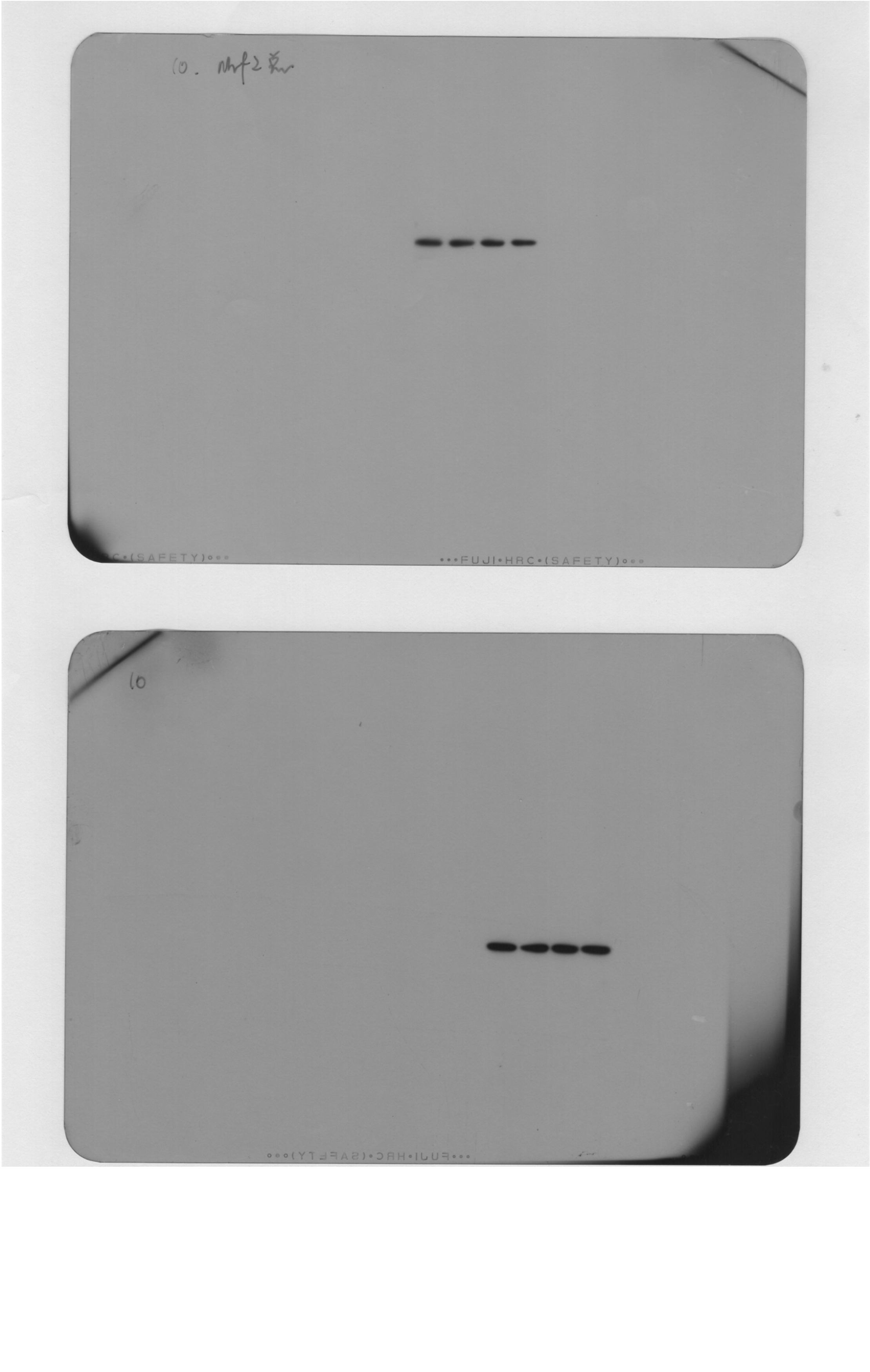

Fig5.A-GPX4

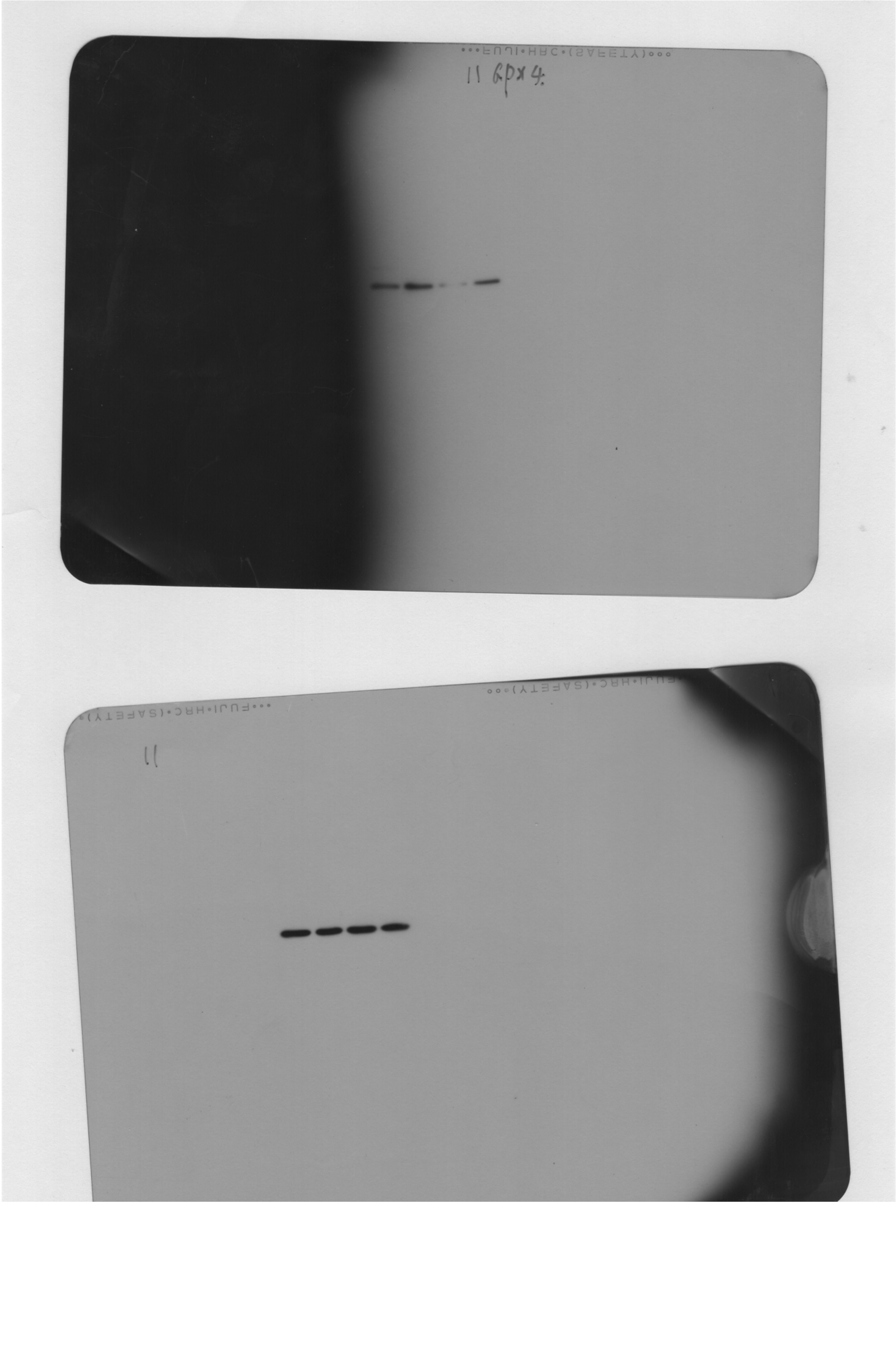

Fig5 I GPX4

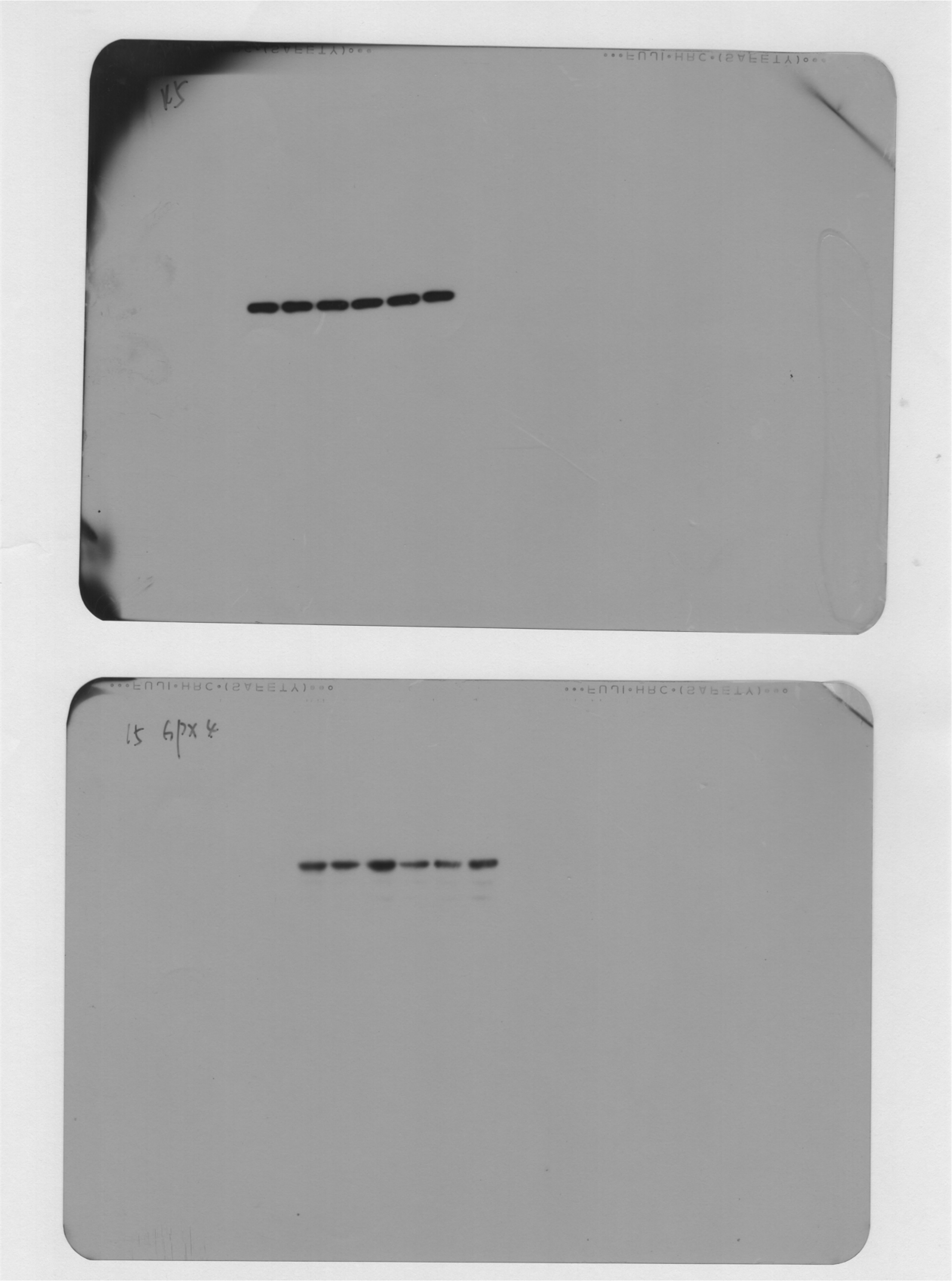

Fig5 I NR F2 nuclear

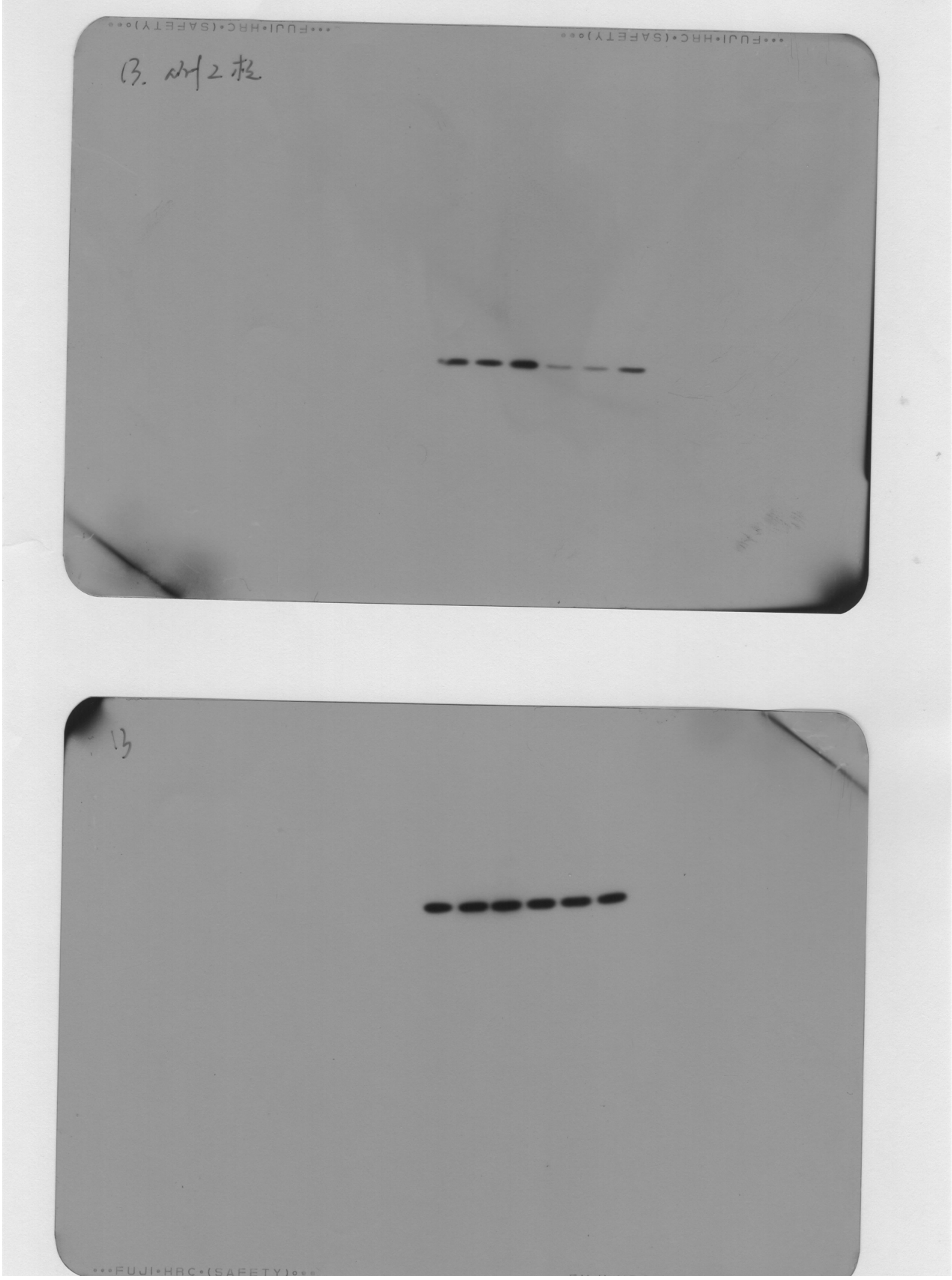

Fig5I NRF2 total

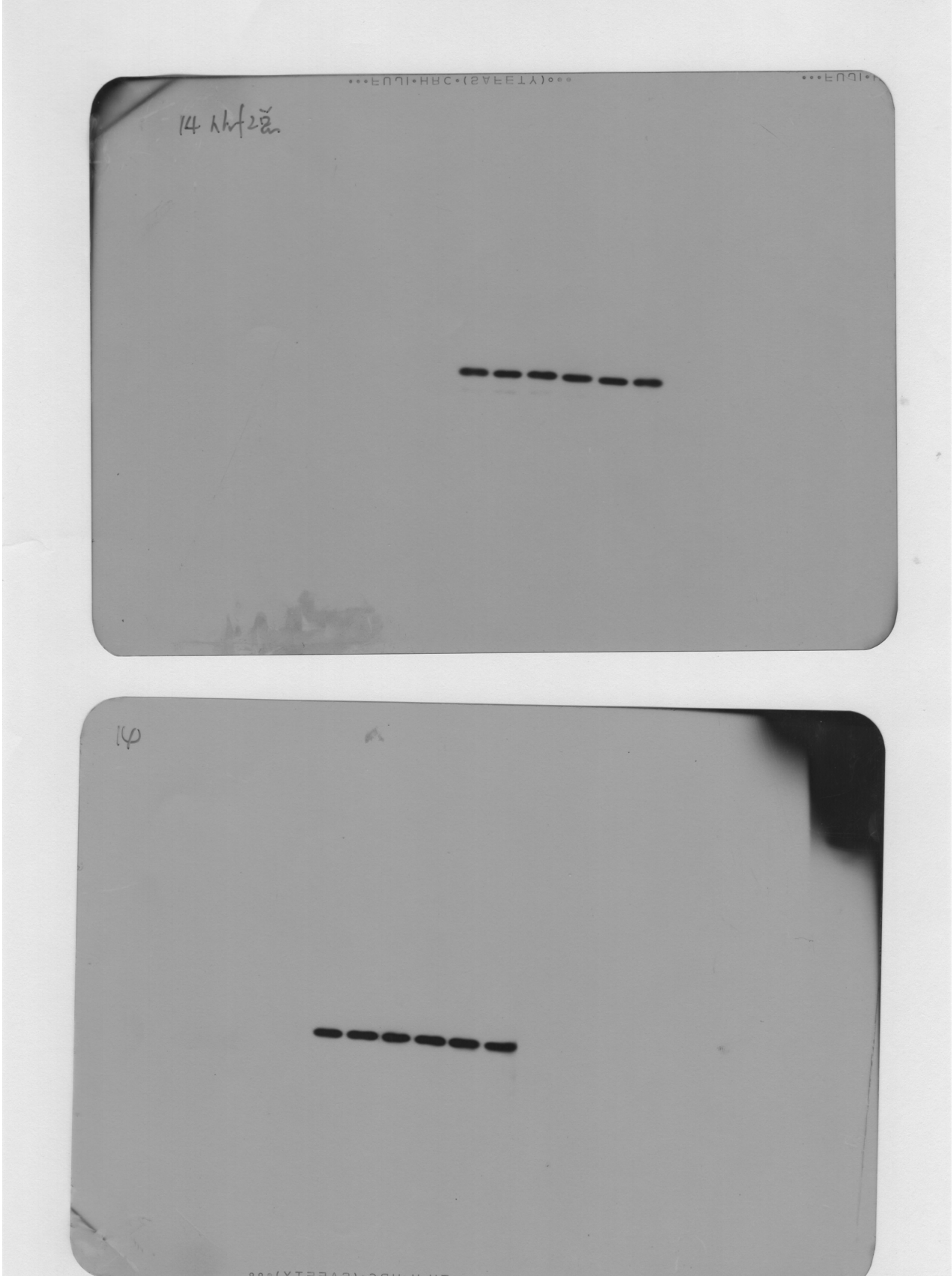

Fig.5.I PHKG2

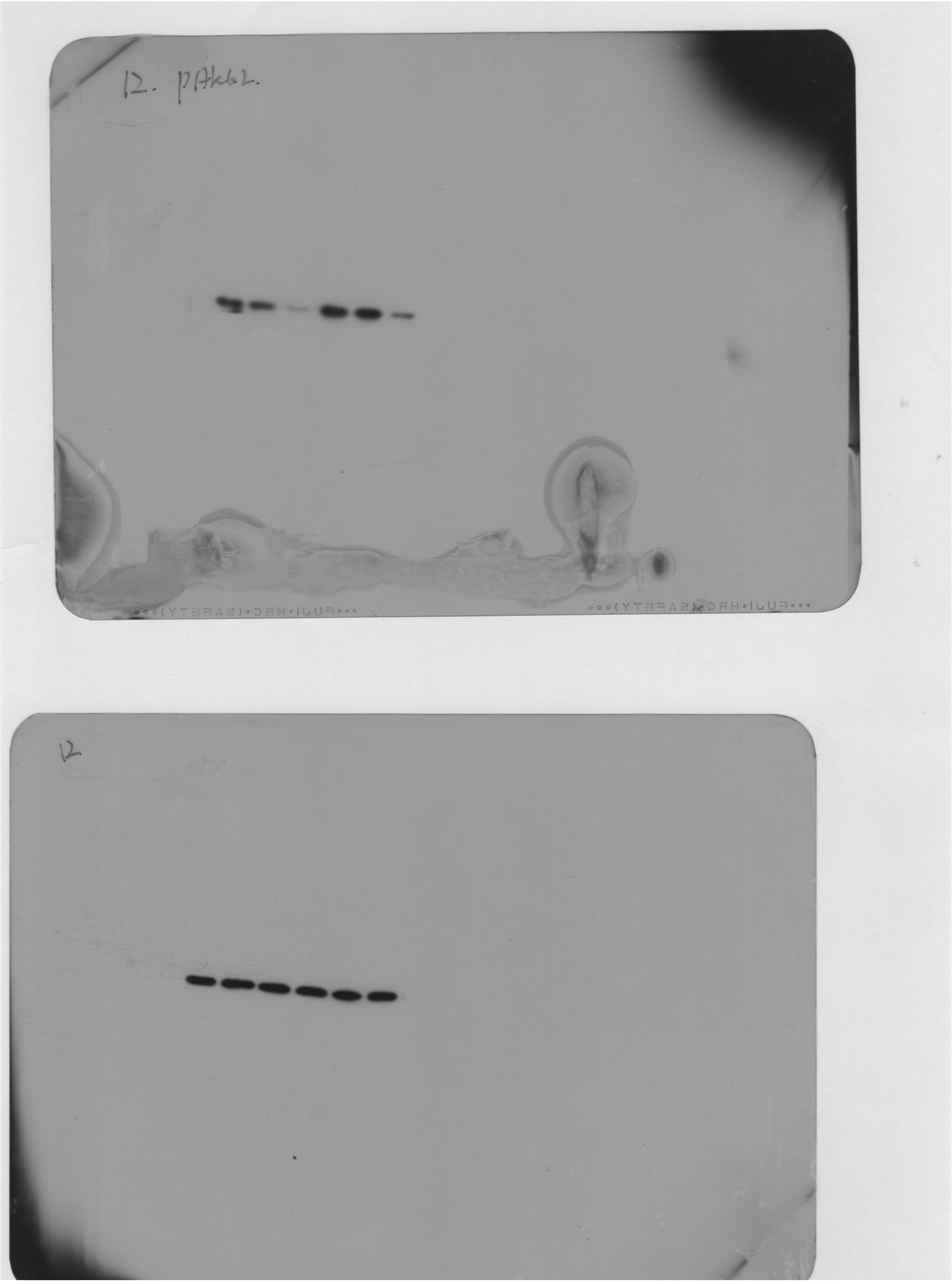

Fig.6.C

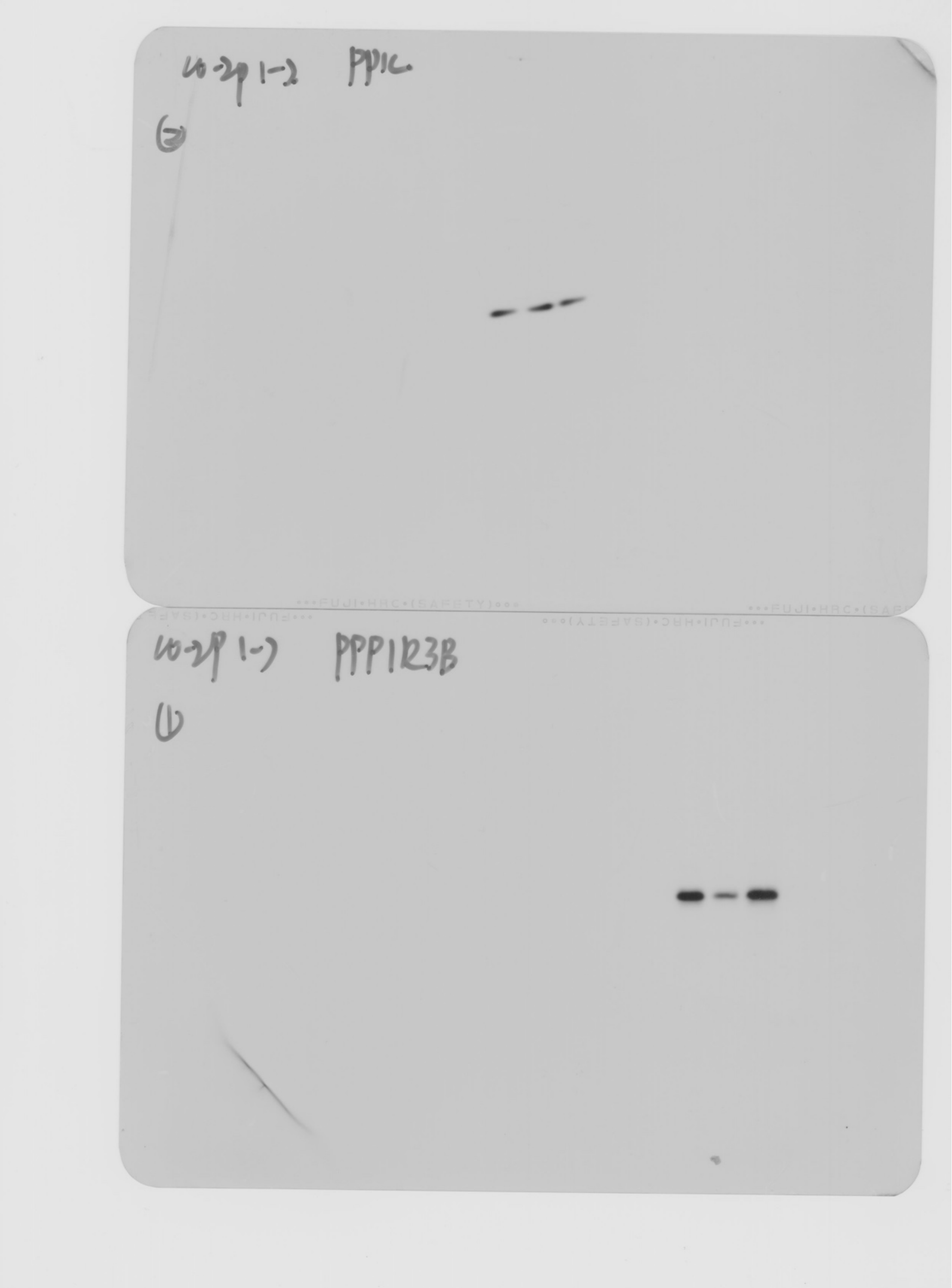

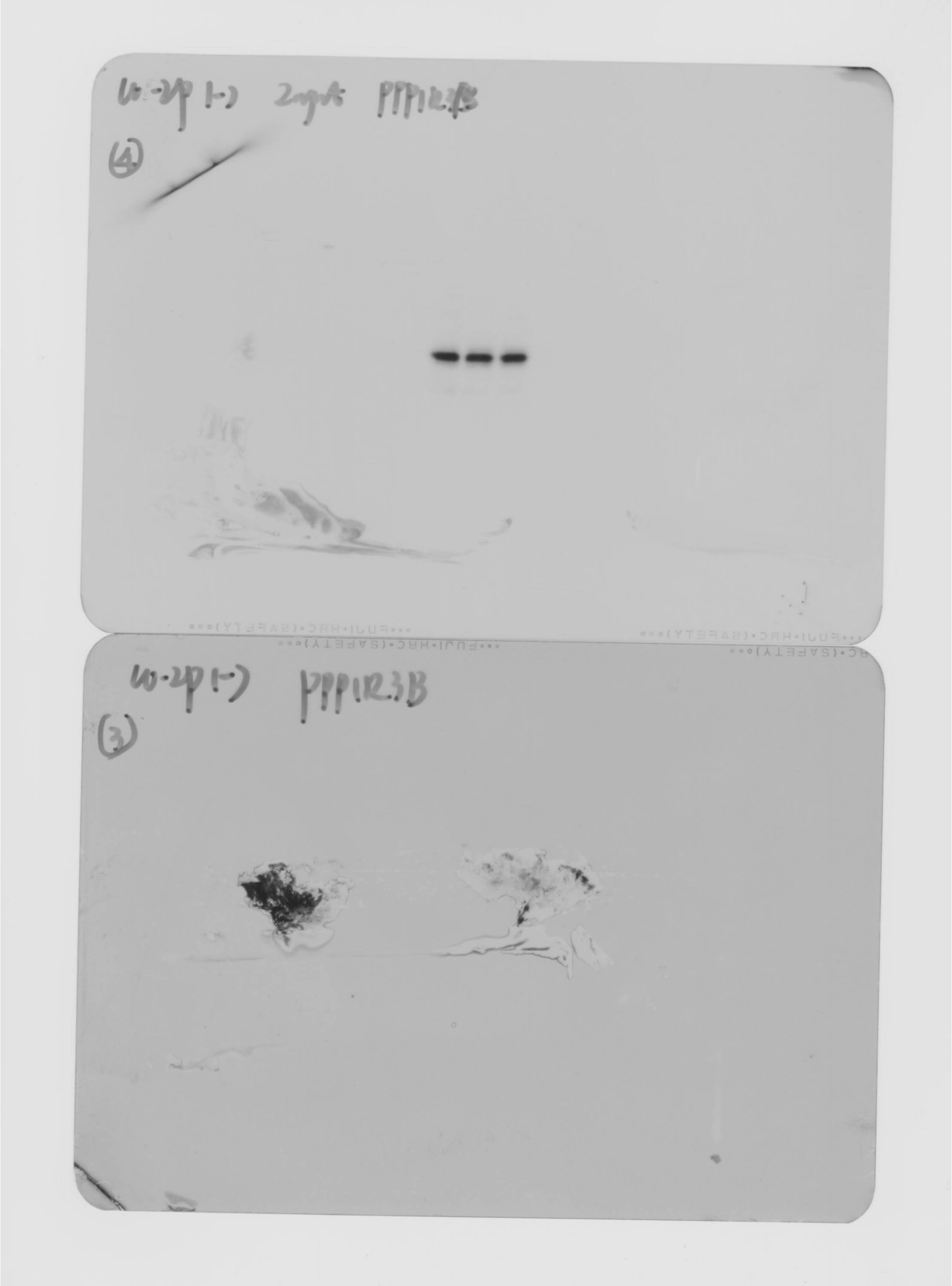
PPP1R3B

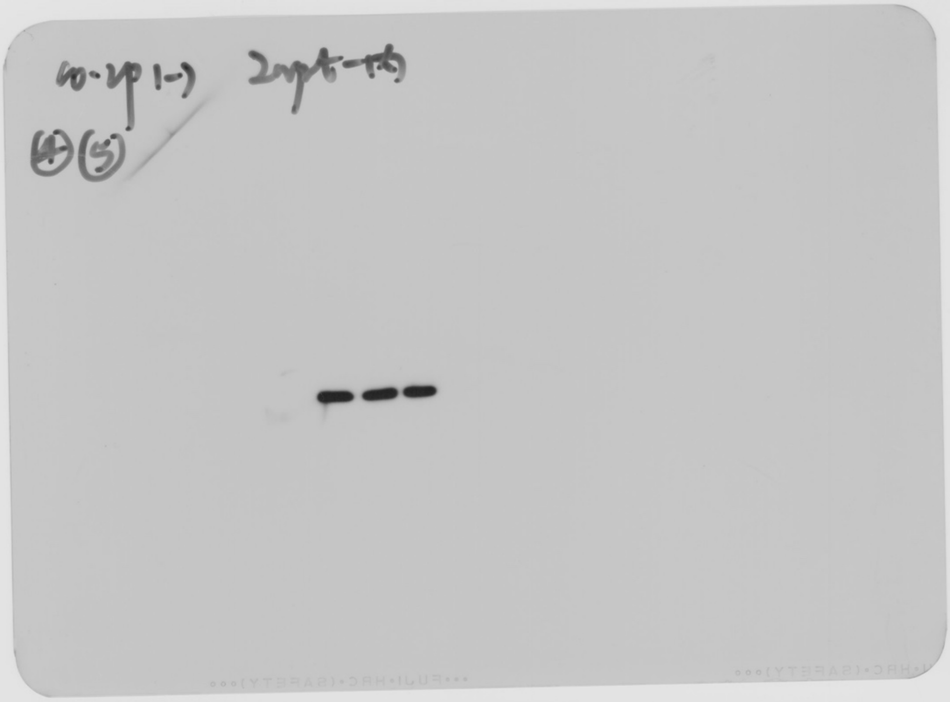

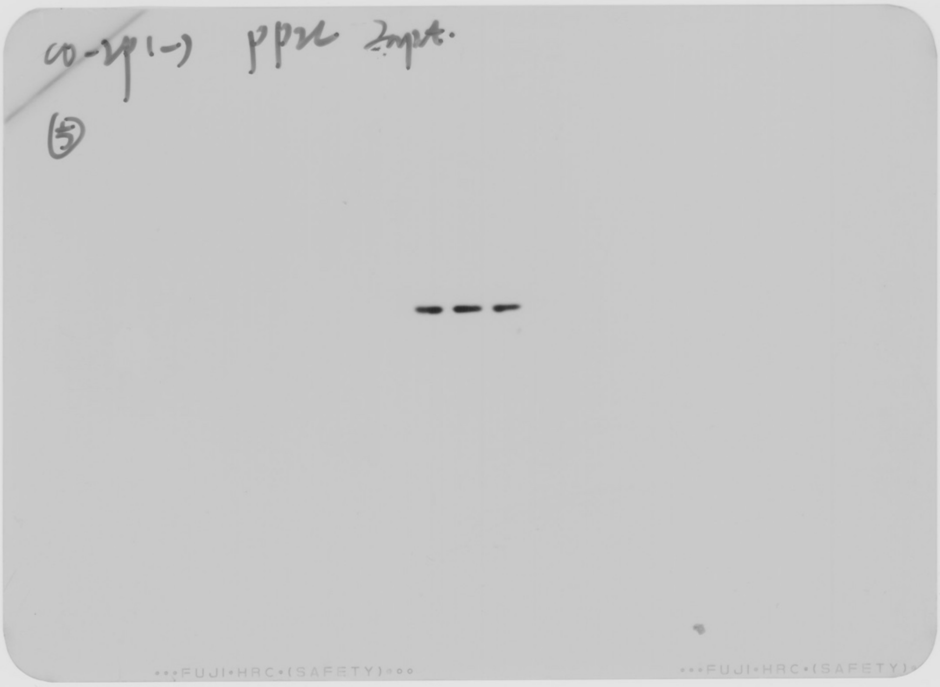
•••FUJI•HRC•(SAFETY)•••
•••FUJI•HRC•(SAFETY)•

Fig.6.D

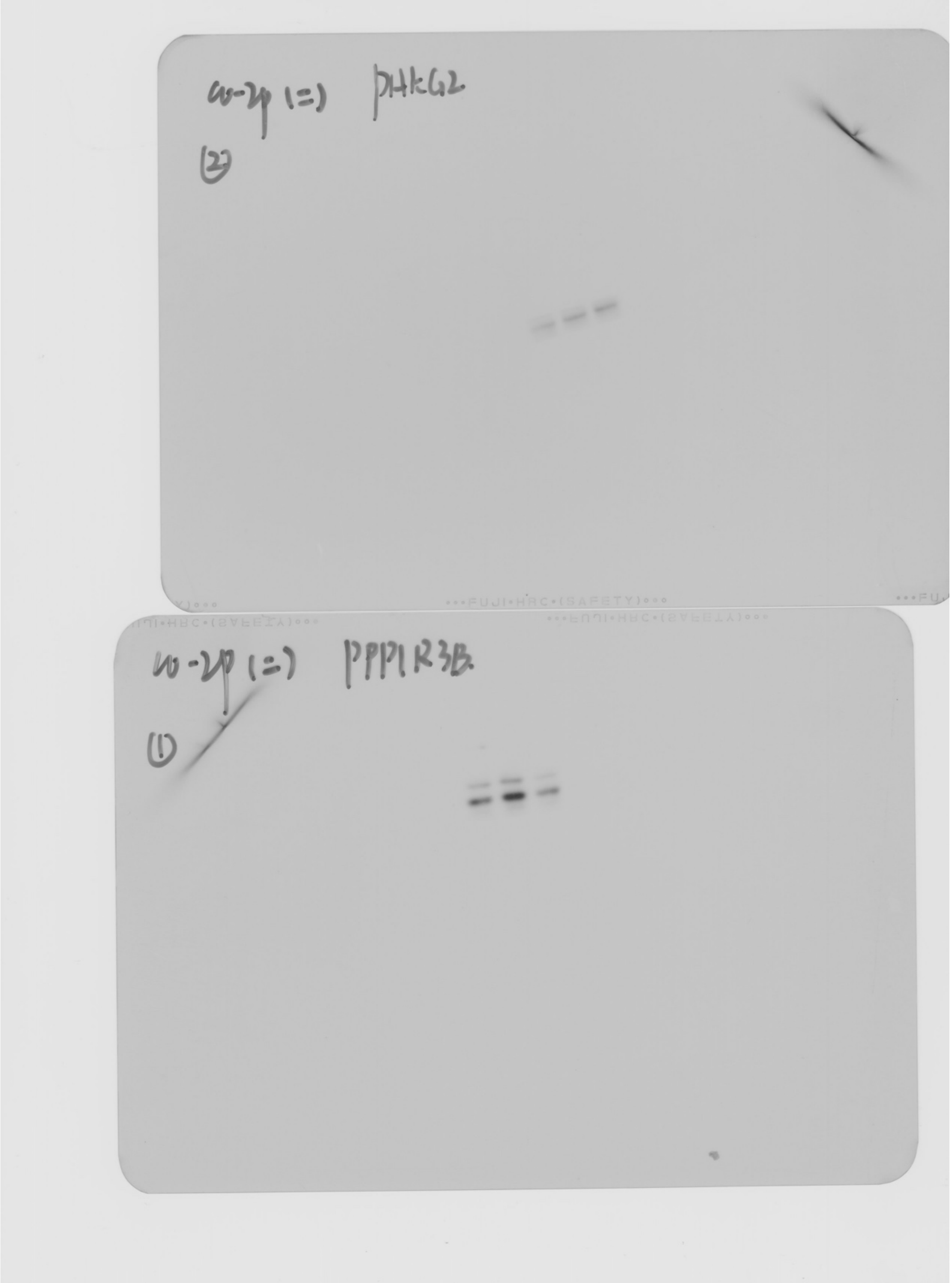

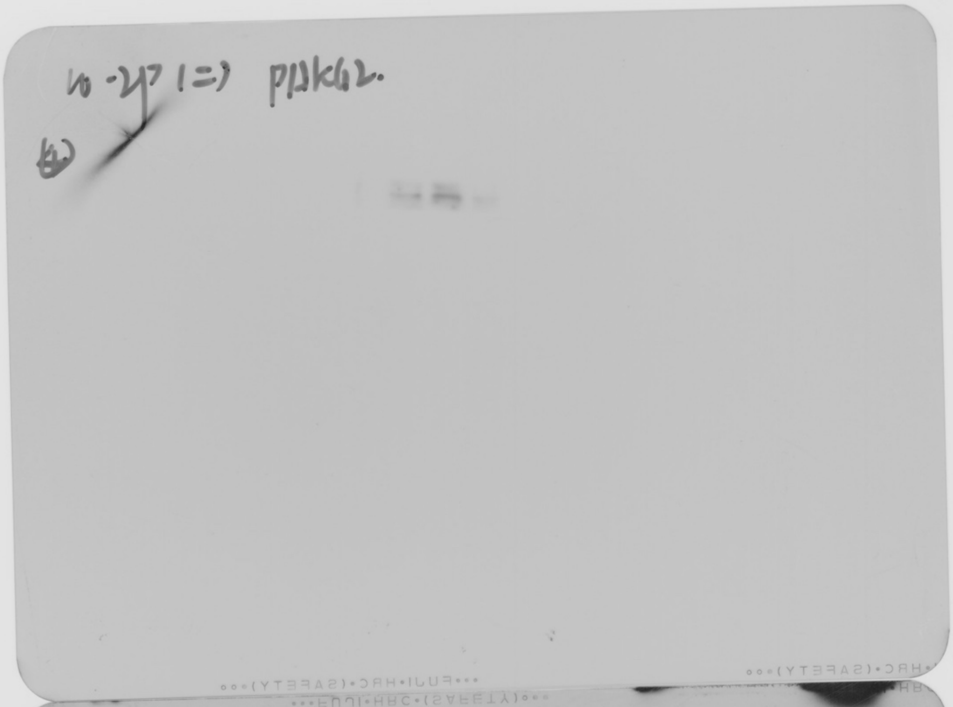

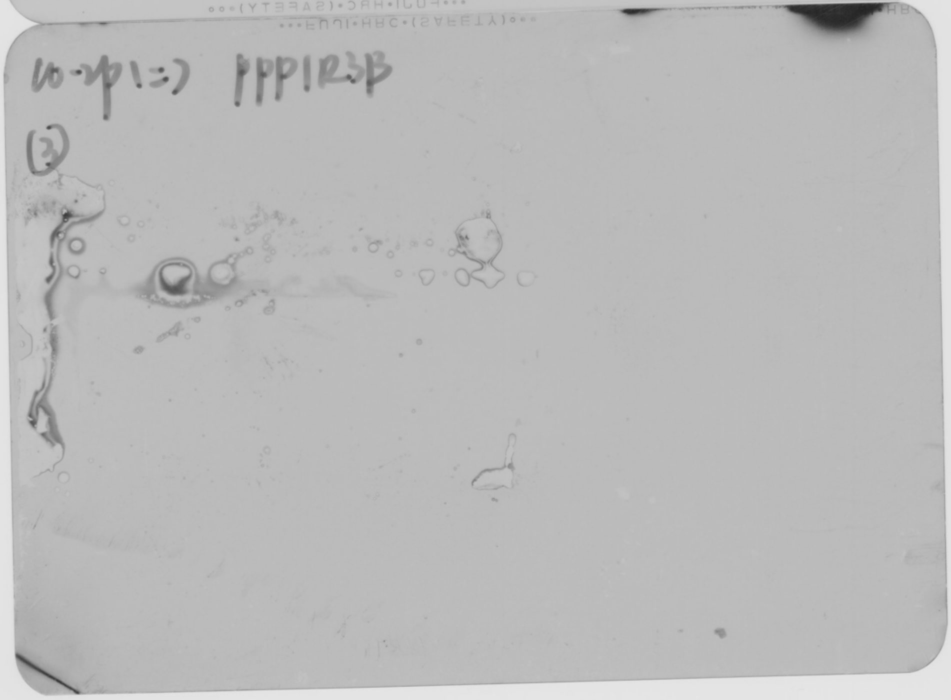
PPP1R3B
(3)

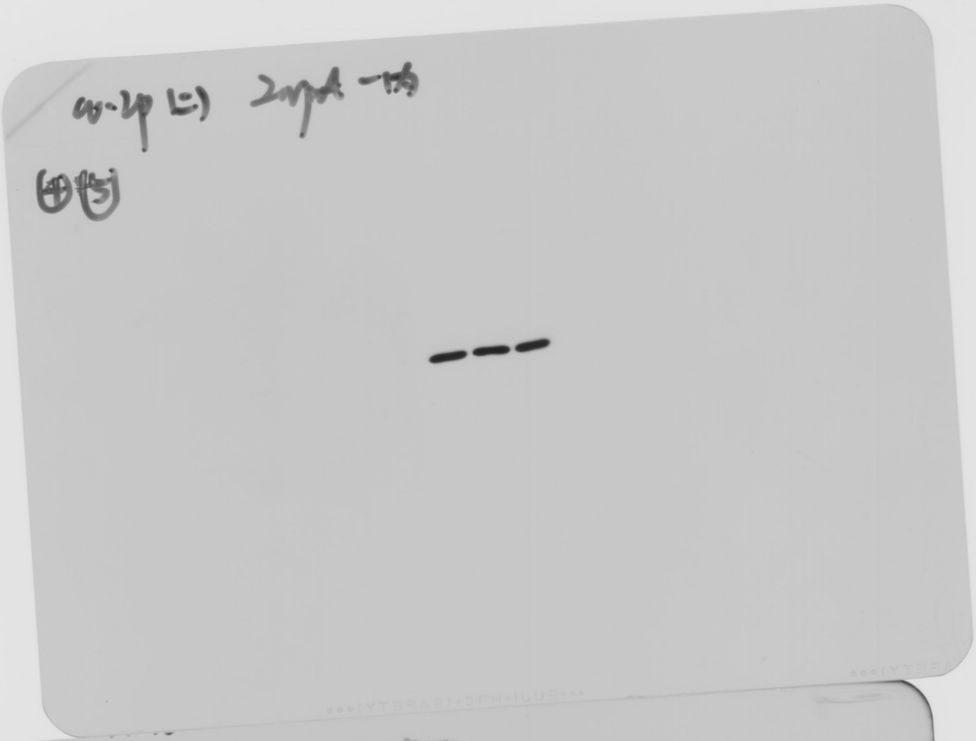

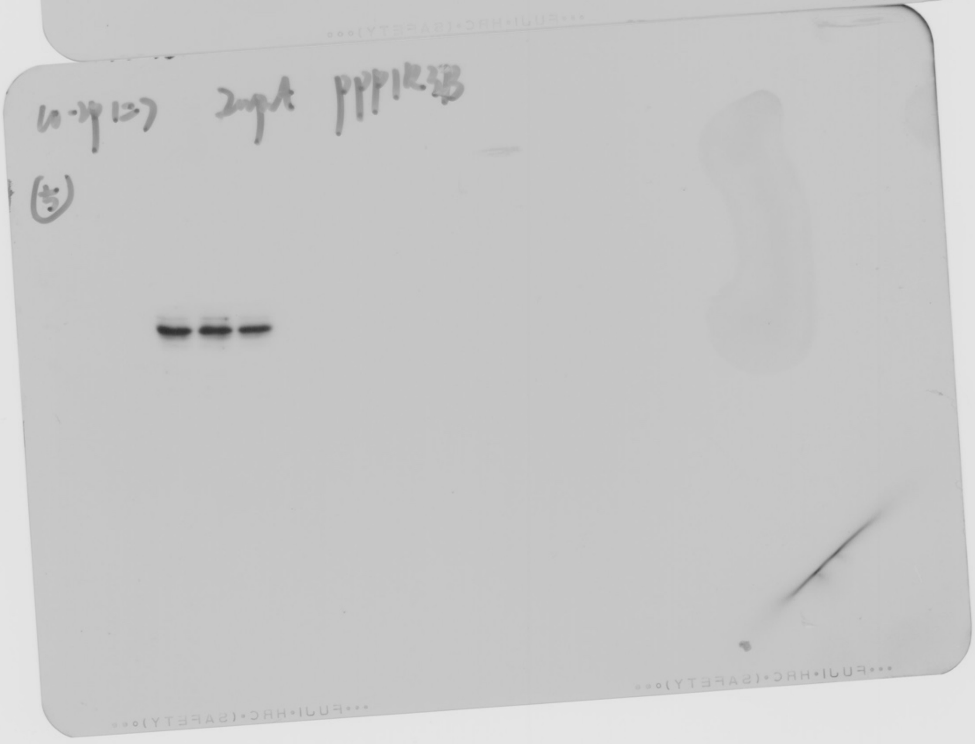
PPP1R3B

Fig.6.E

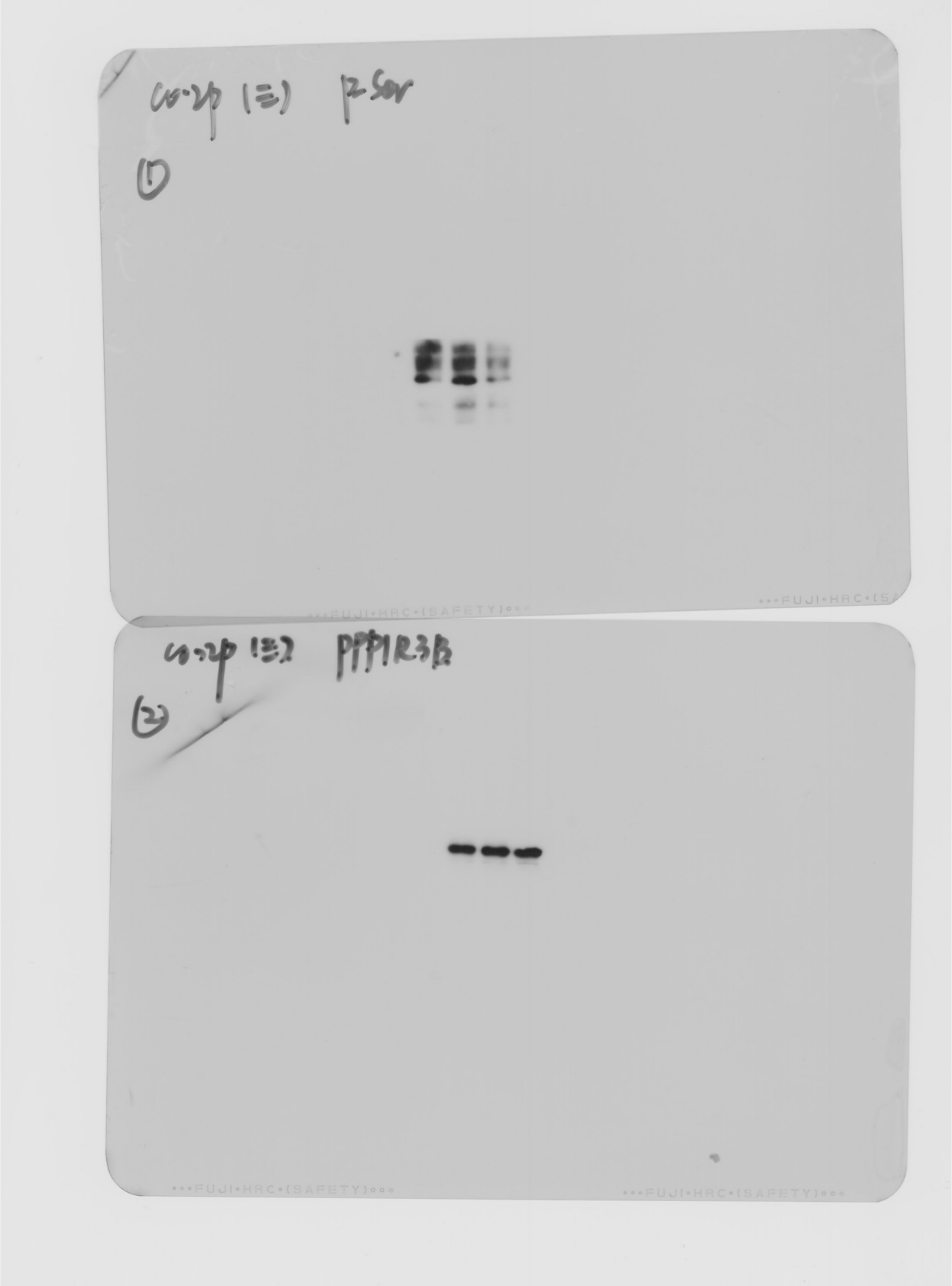

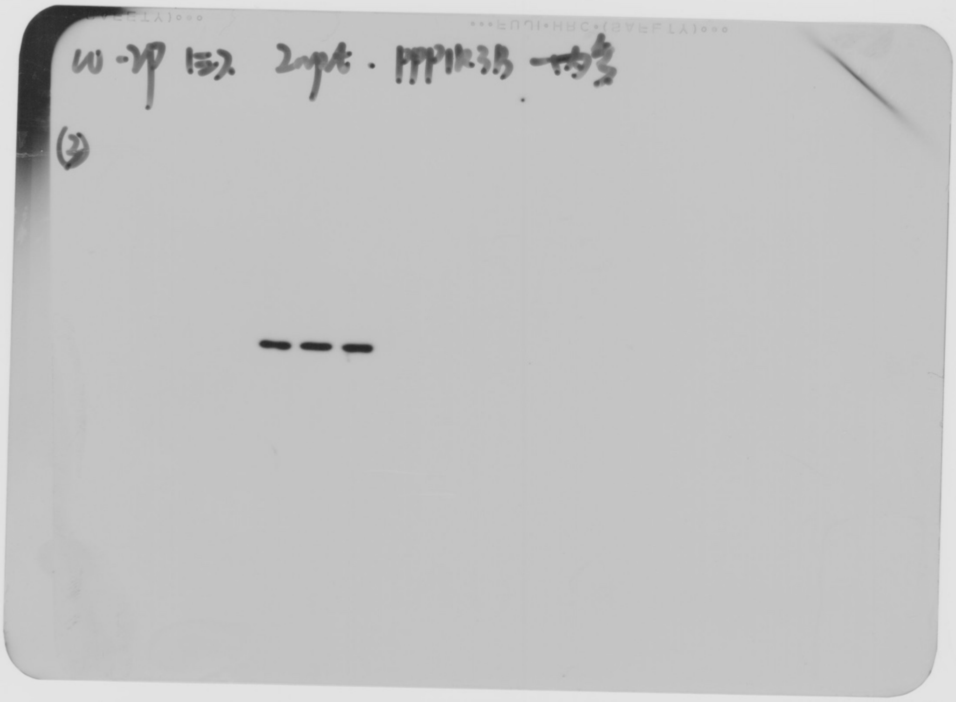
PPP1R3B

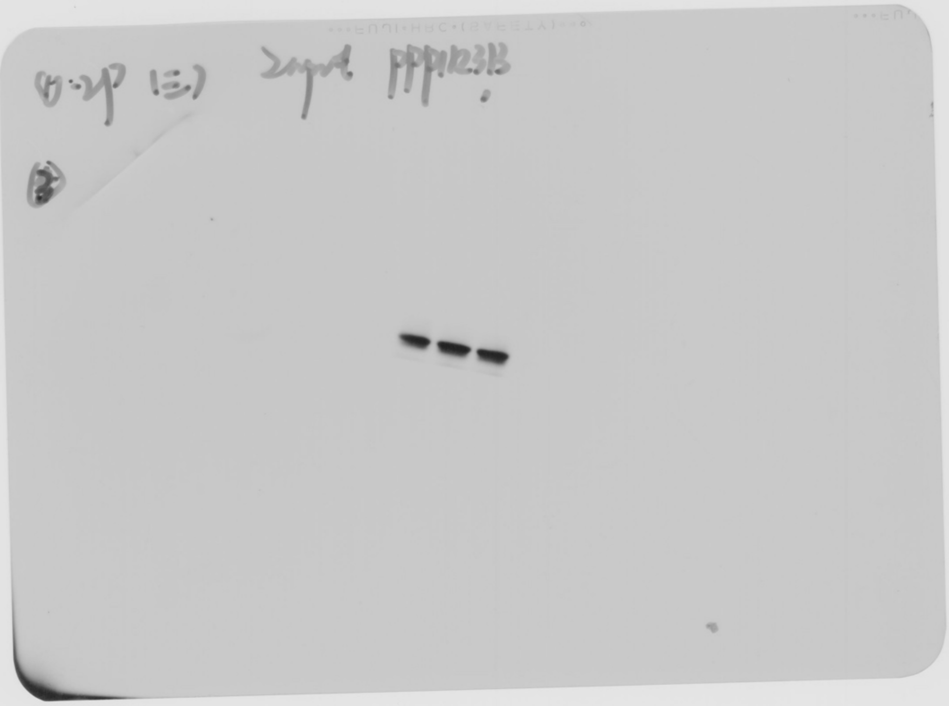
PPP1R3B

Fig.6.B PHKG2

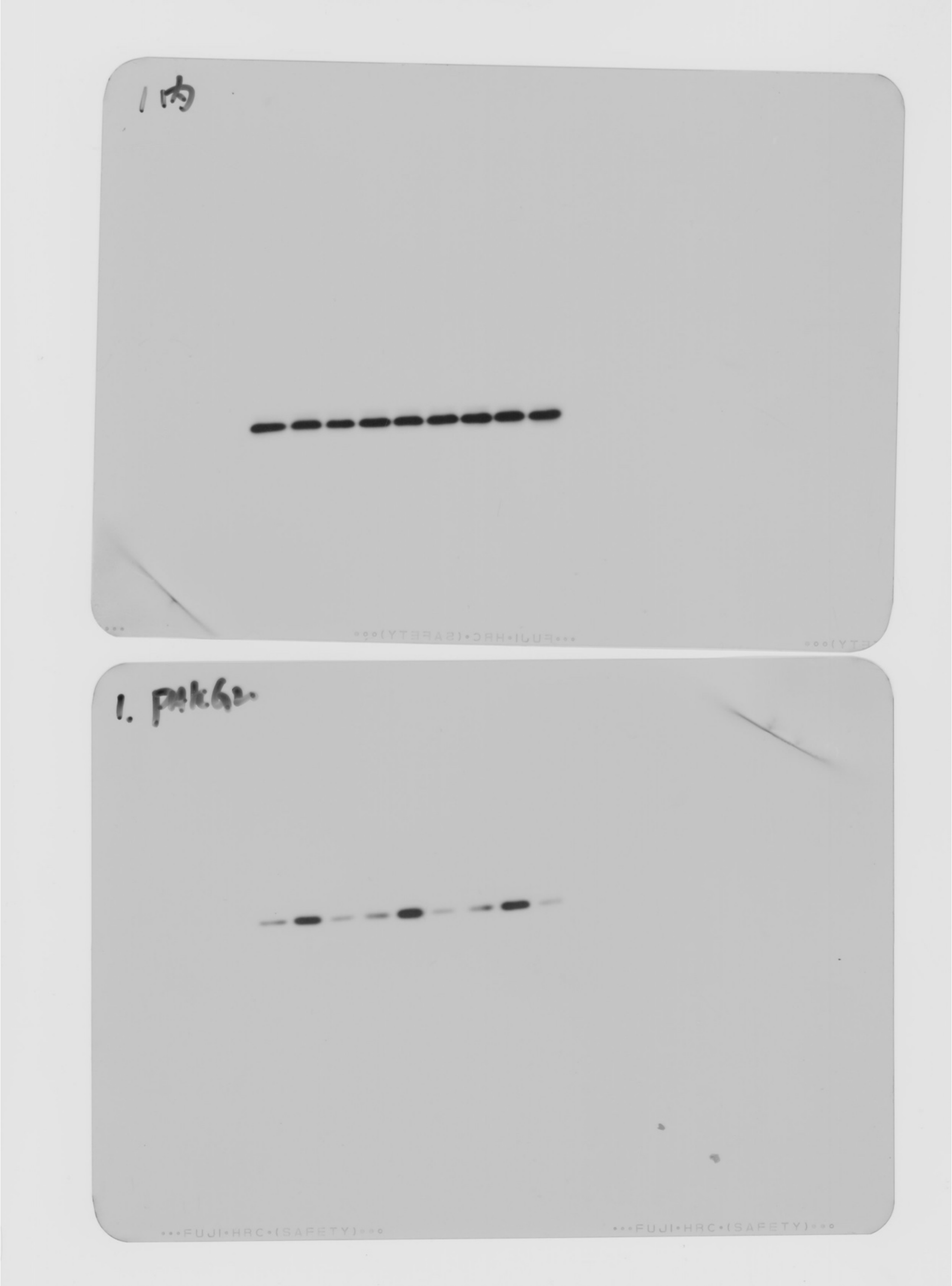

Fig.6.B p-GSK3**b**

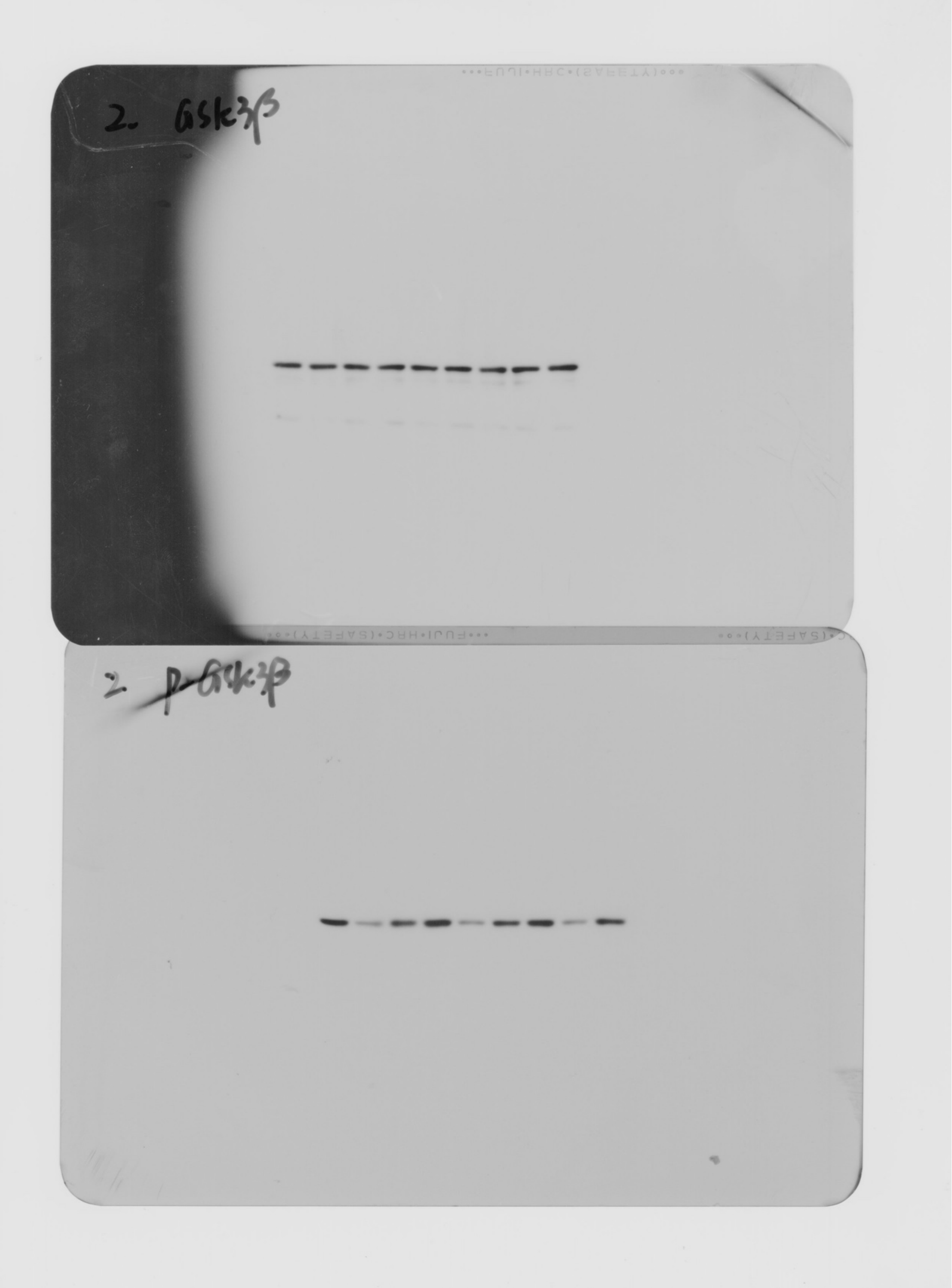

Fig.6.B b-actin

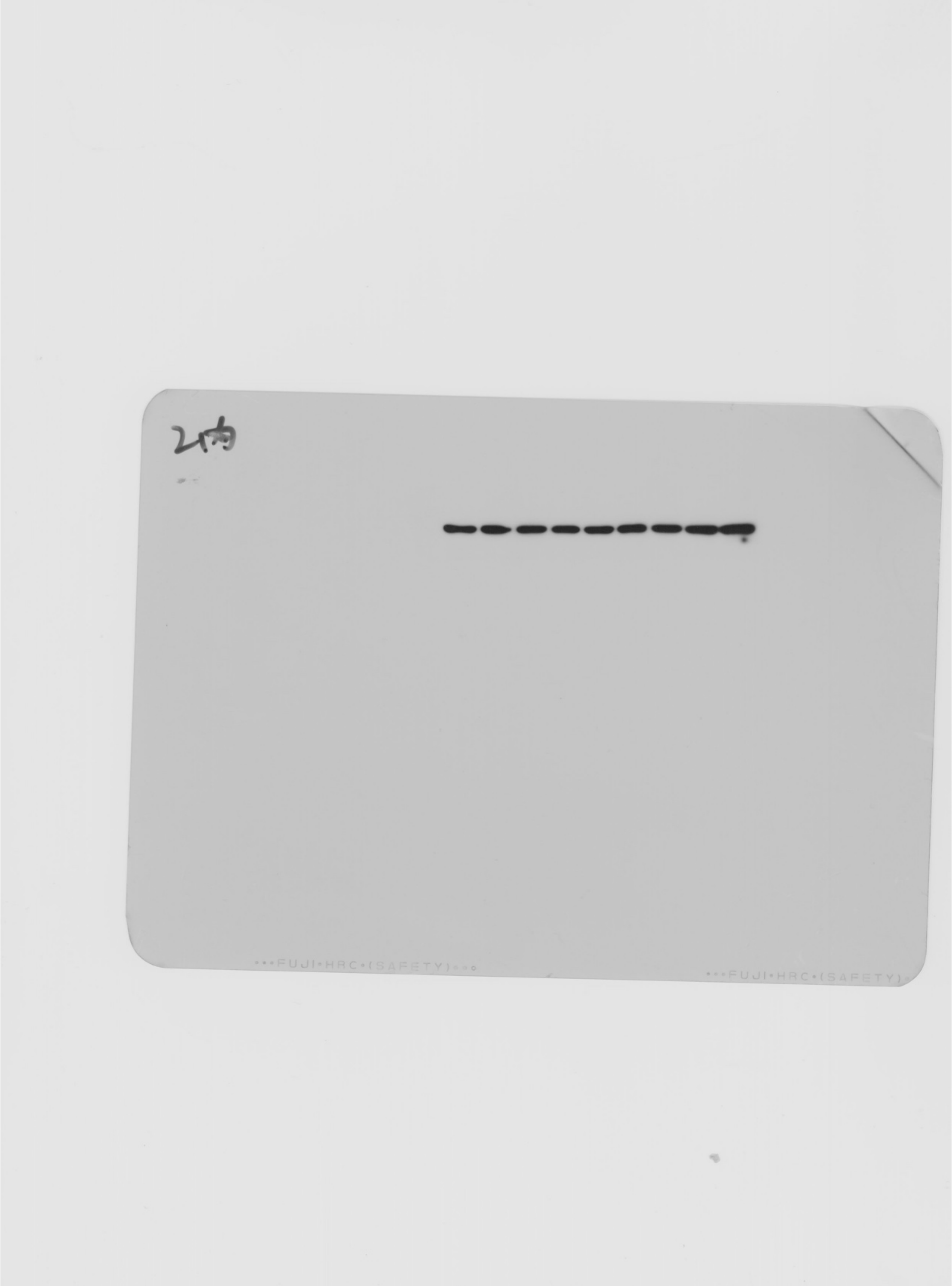

Fig.6.B nucle-NRF2

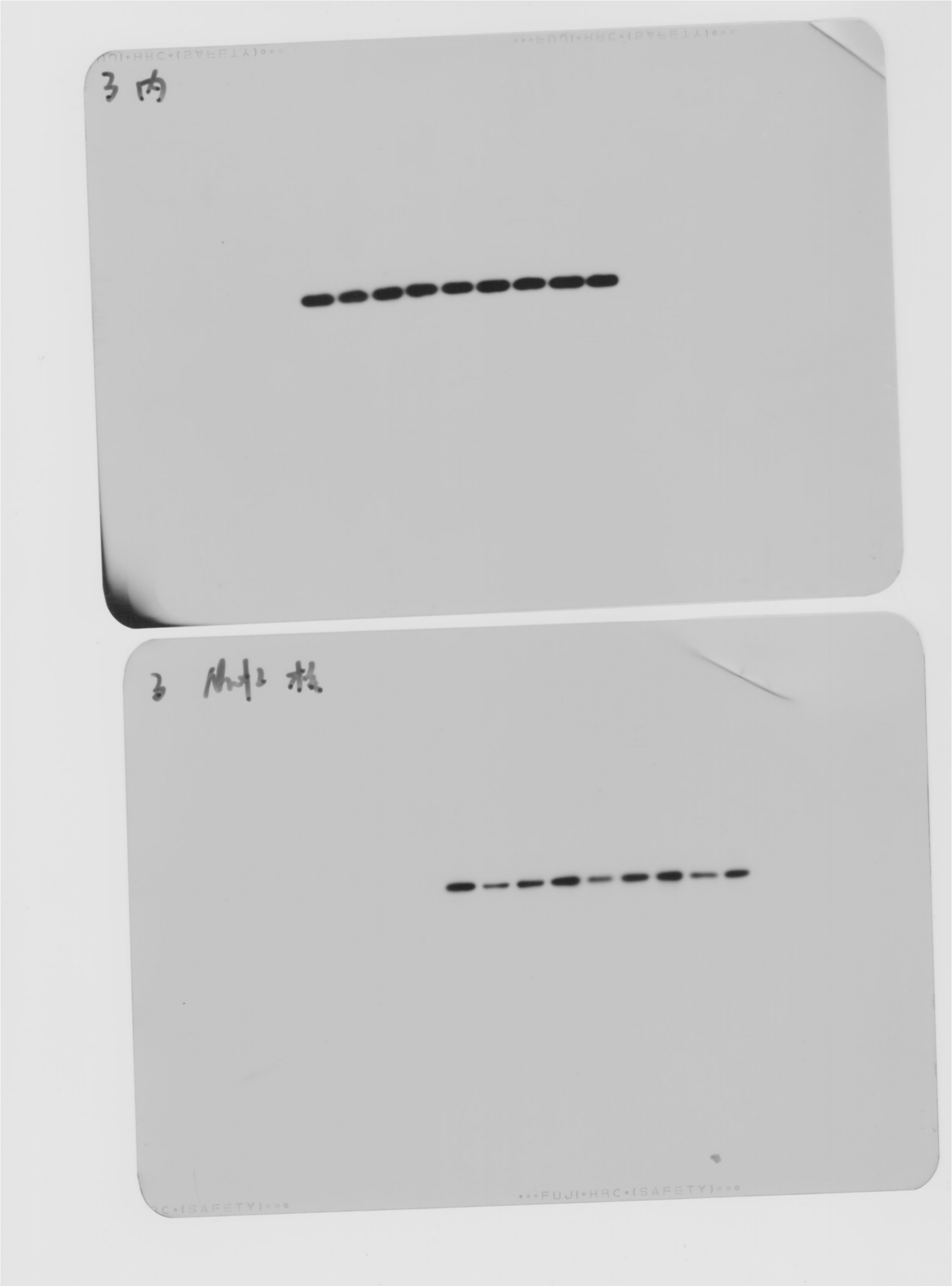

Fig.6.B total -NRF2

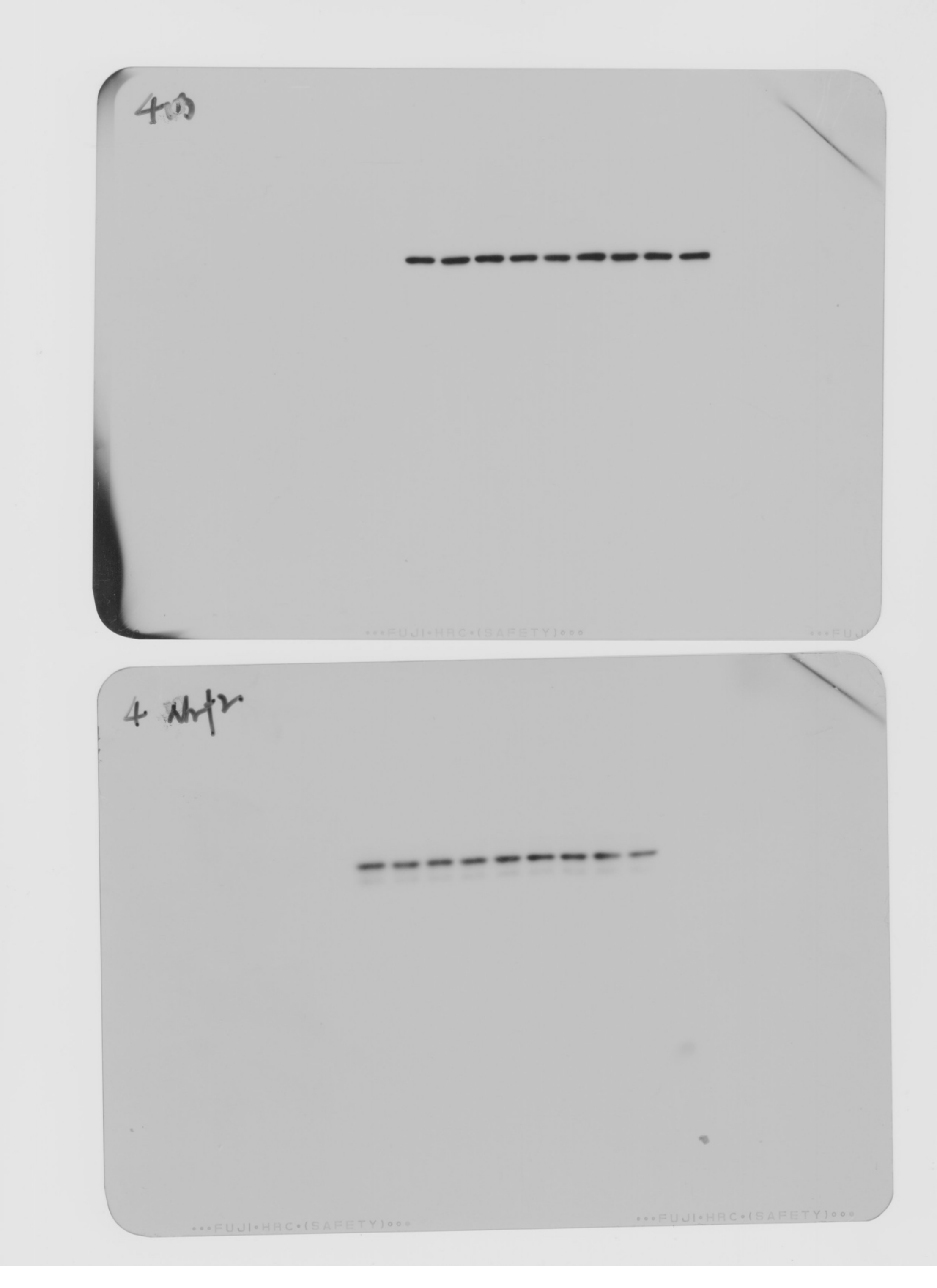

Fig.6.F GSK3b

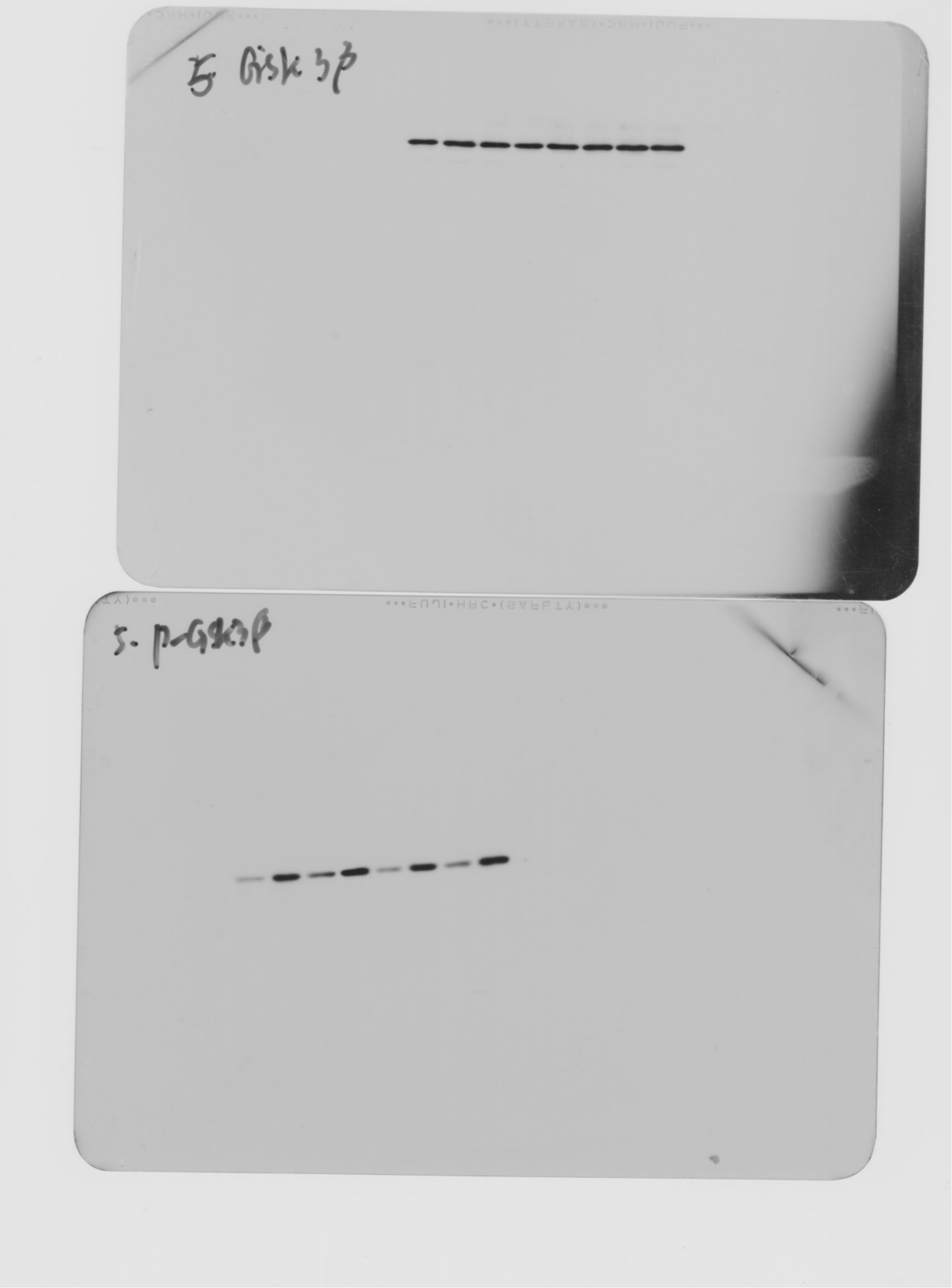

Fig.6.F b-actin

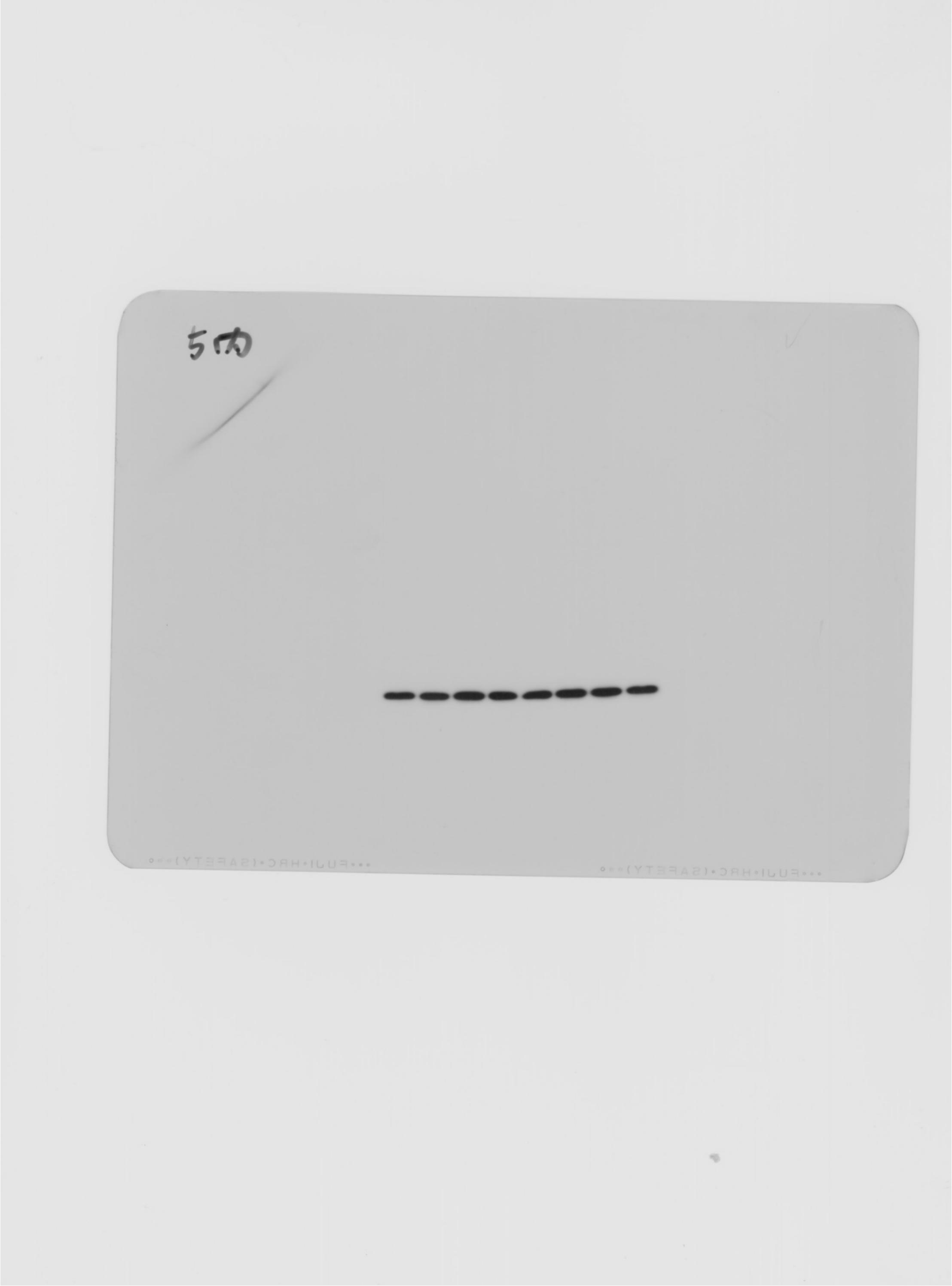

Fig.6.F nucle NRF2

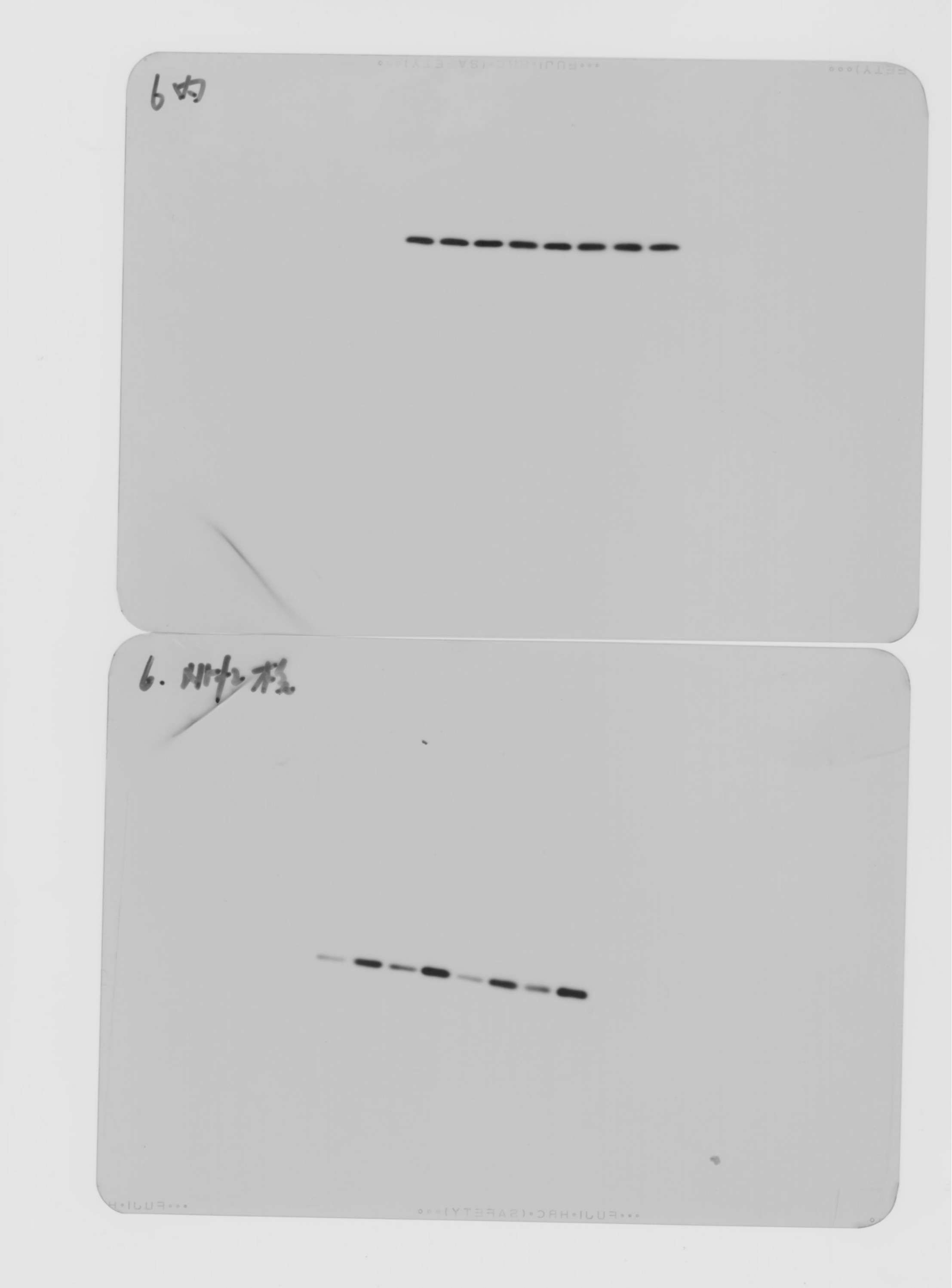

Fig.6.F total NRF2

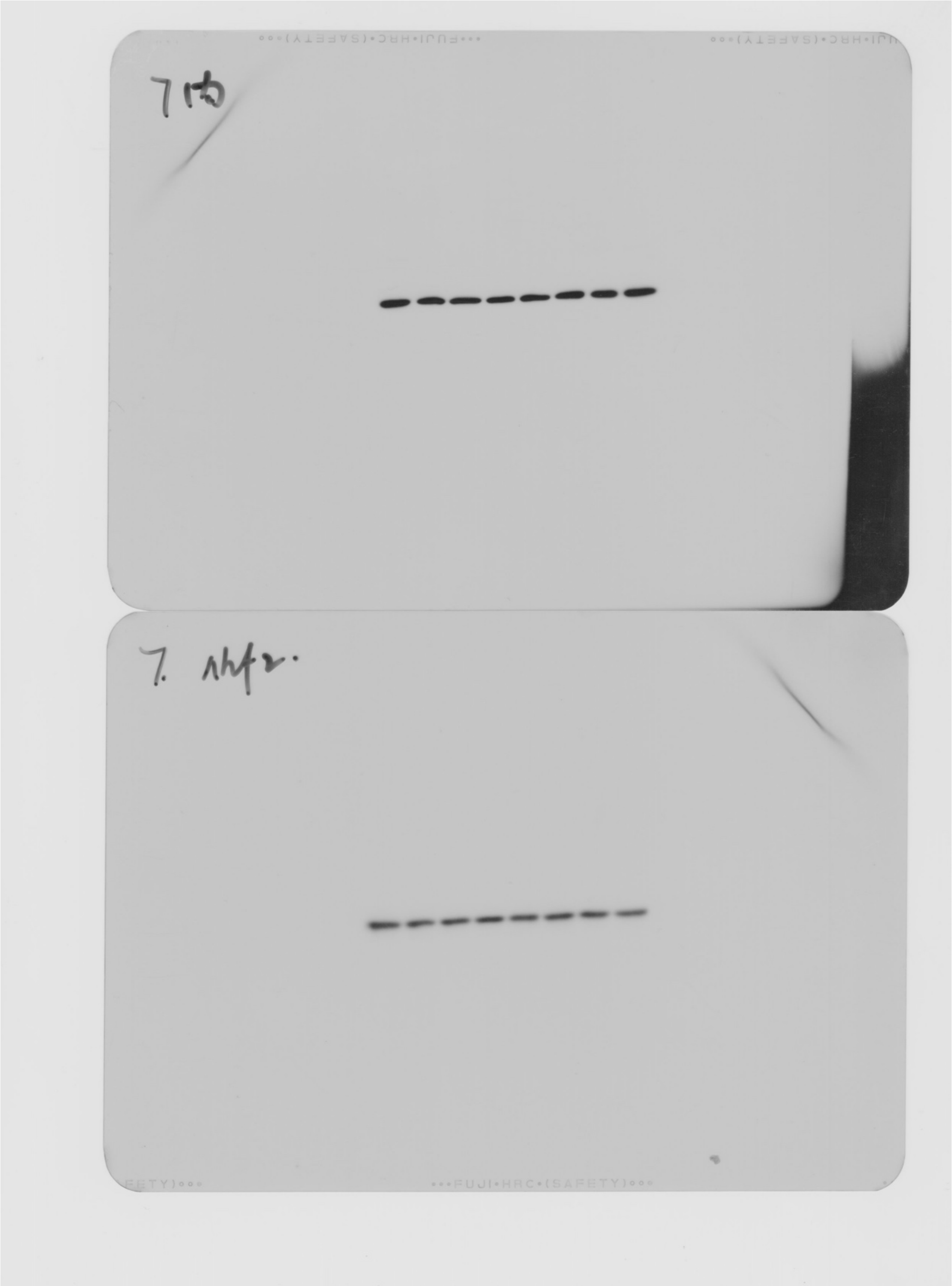

Fig.6.G-nucle NRF2

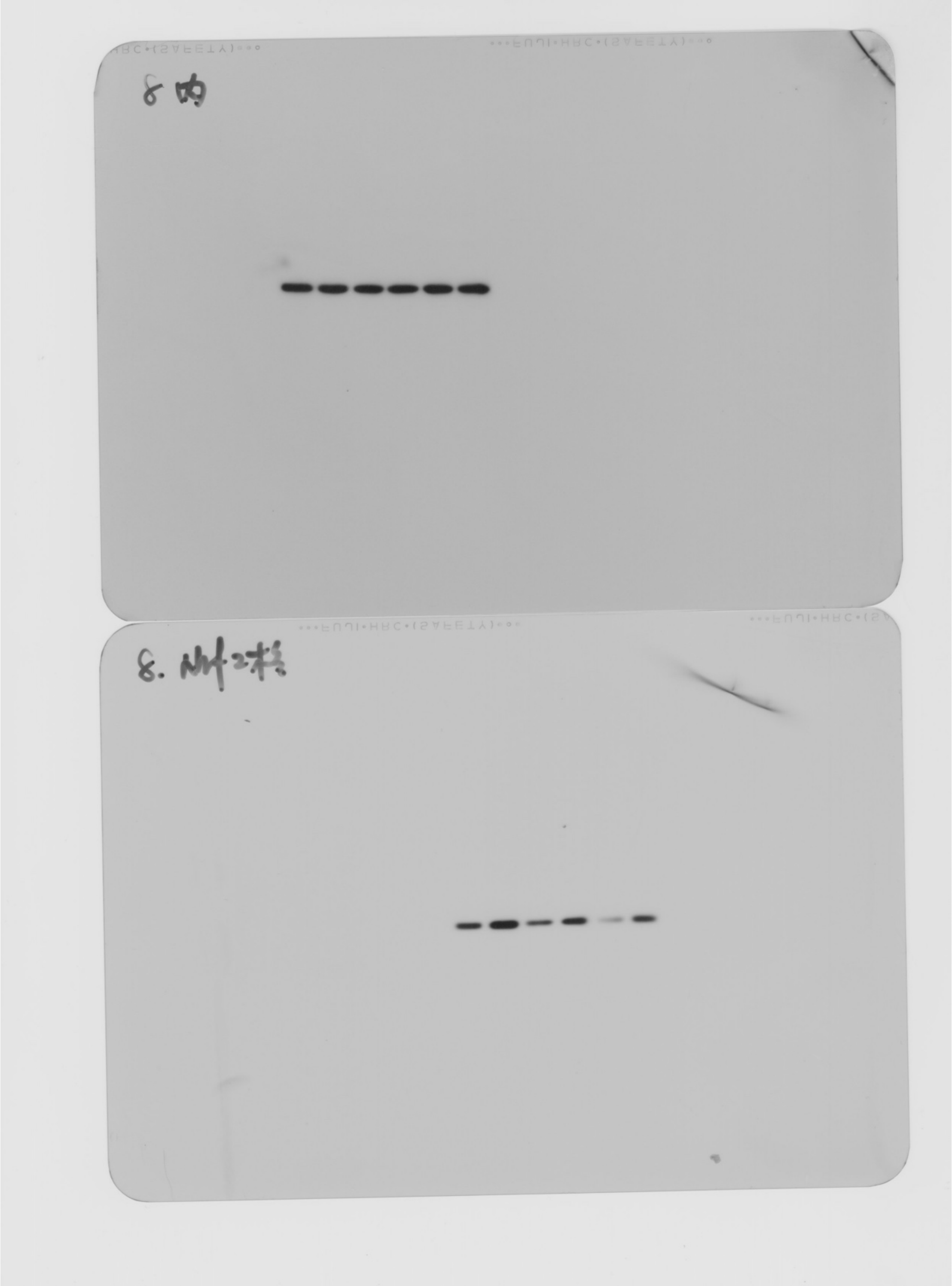

Fig.6.G Total NRF2

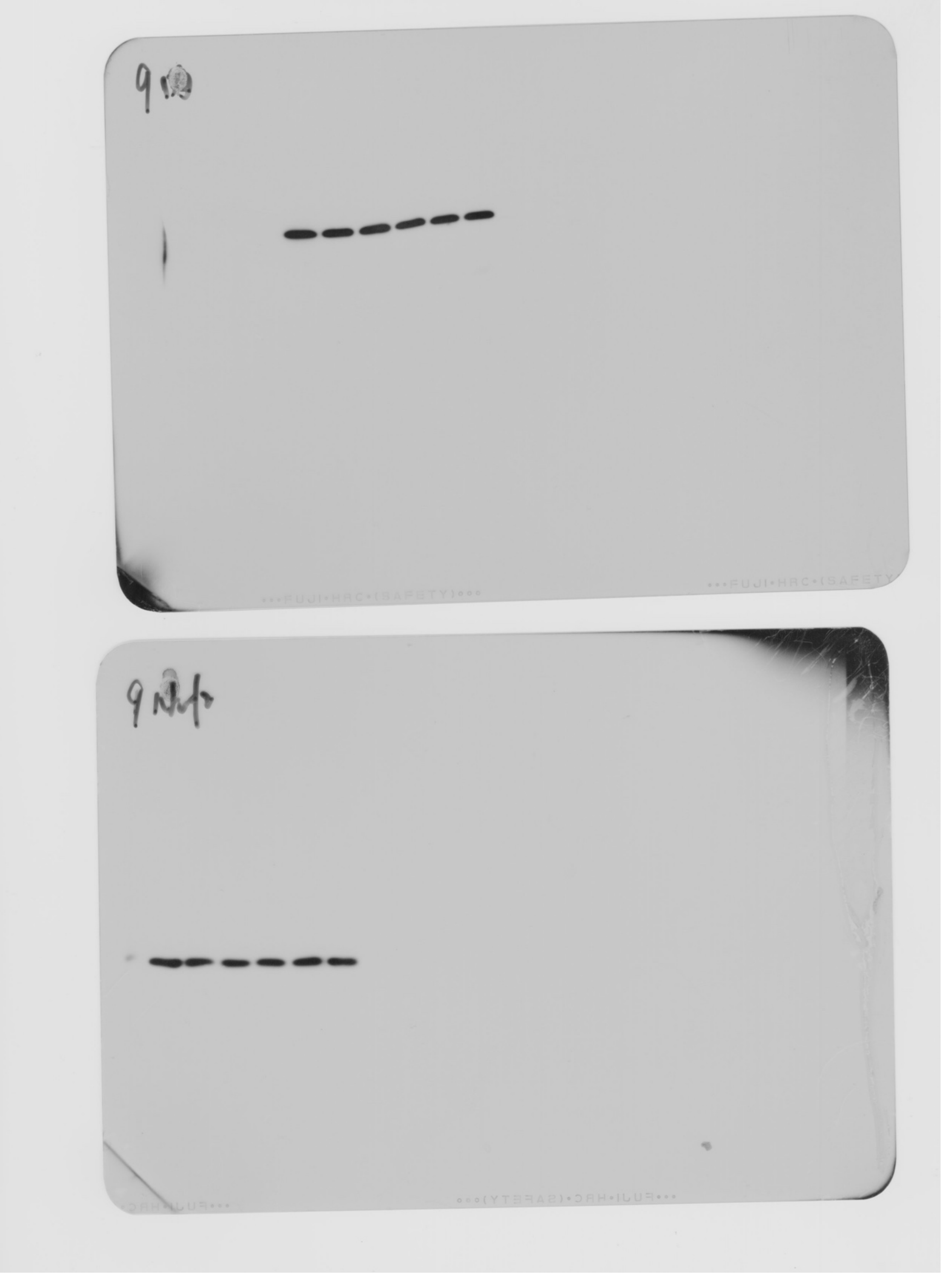

Fig4 F PHKG2

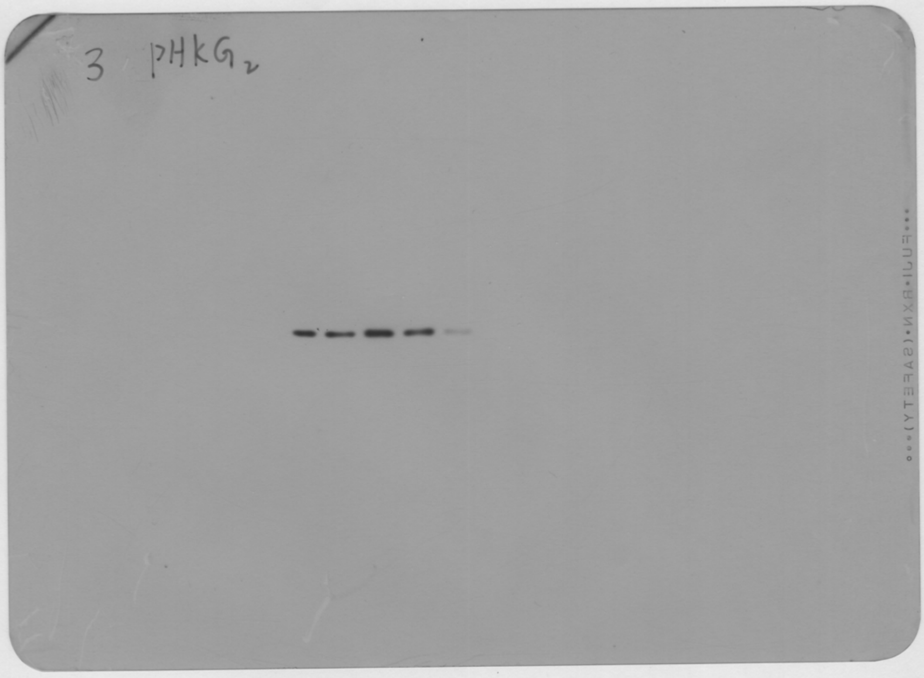
3 pHKG2

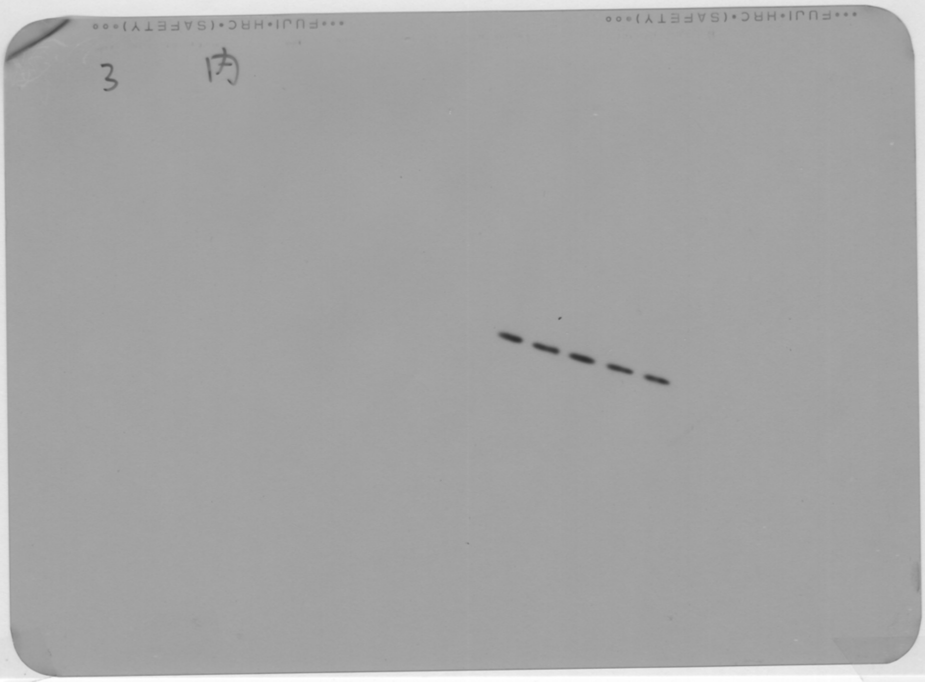
3 内

ChIP

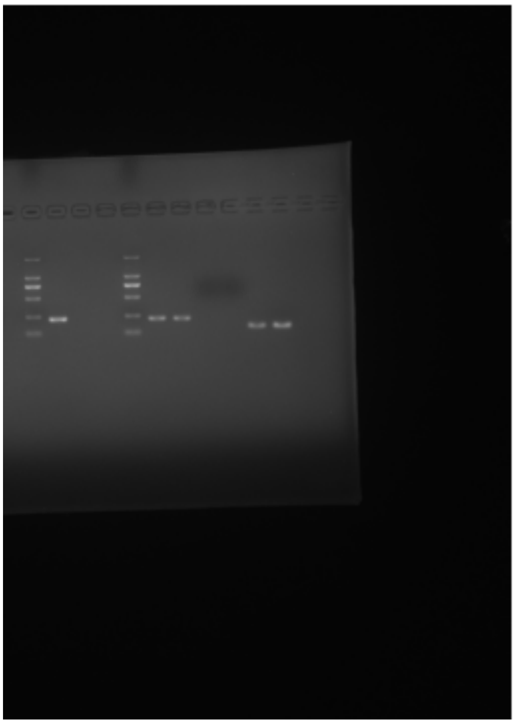

Supplement: Supplementary file 3 — Supplementary Western Blot [file 41419_2025_7985_MOESM3_ESM.pdf]
