# Supplementary figures and images for: Transcriptional activation of PHKG2 by TP53 promotes ferroptosis through nuclear export of NRF2 in head and neck squamous cell carcinoma
Source: Cell Death Dis. 2025 Aug 30;16(1):662. doi: 10.1038/s41419-025-07985-3 (PMC12398534; doi:10.1038/s41419-025-07985-3)

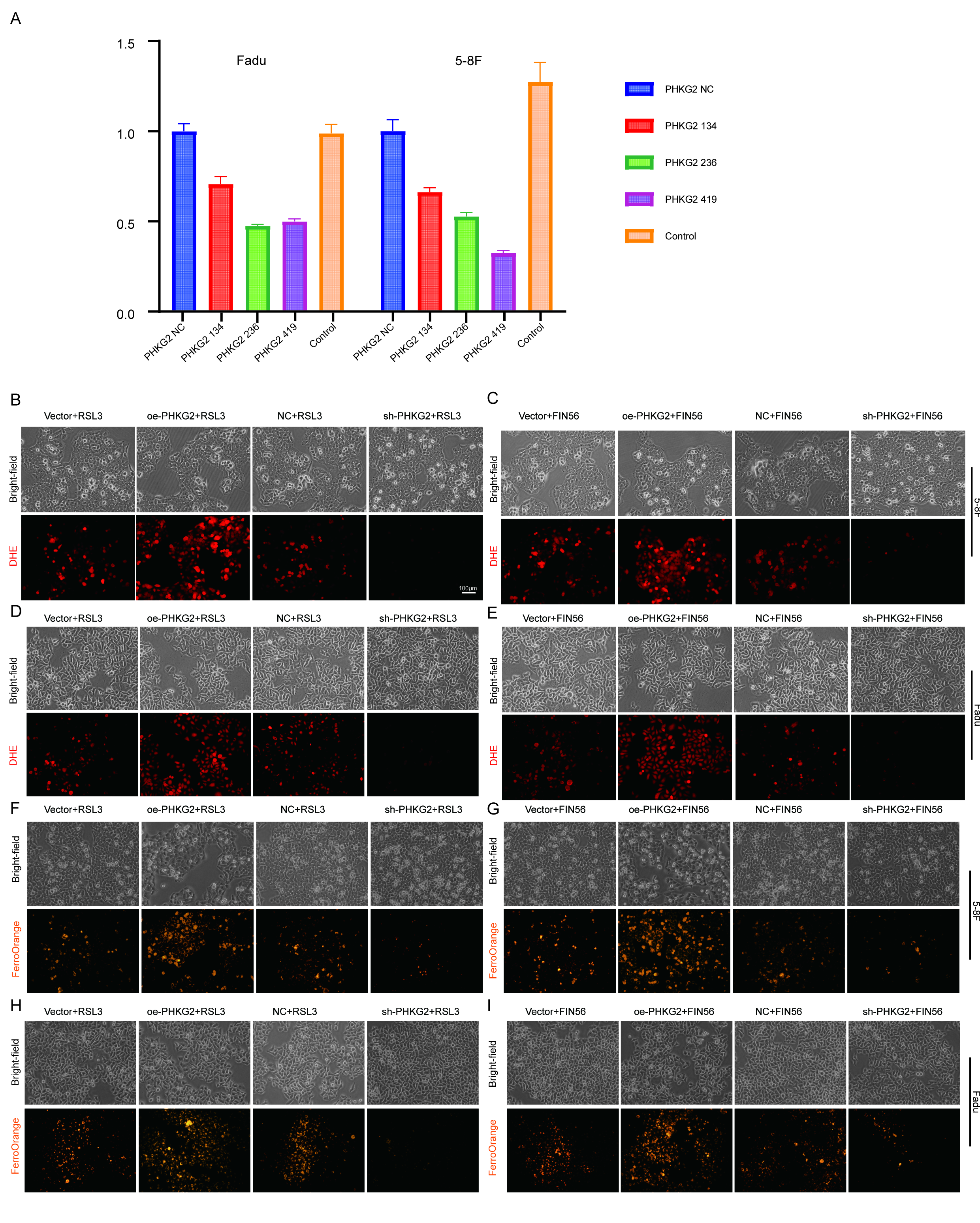

Supplement: Supplementary file 4 — Supplemental Figure 1 [file 41419_2025_7985_MOESM4_ESM.tif]

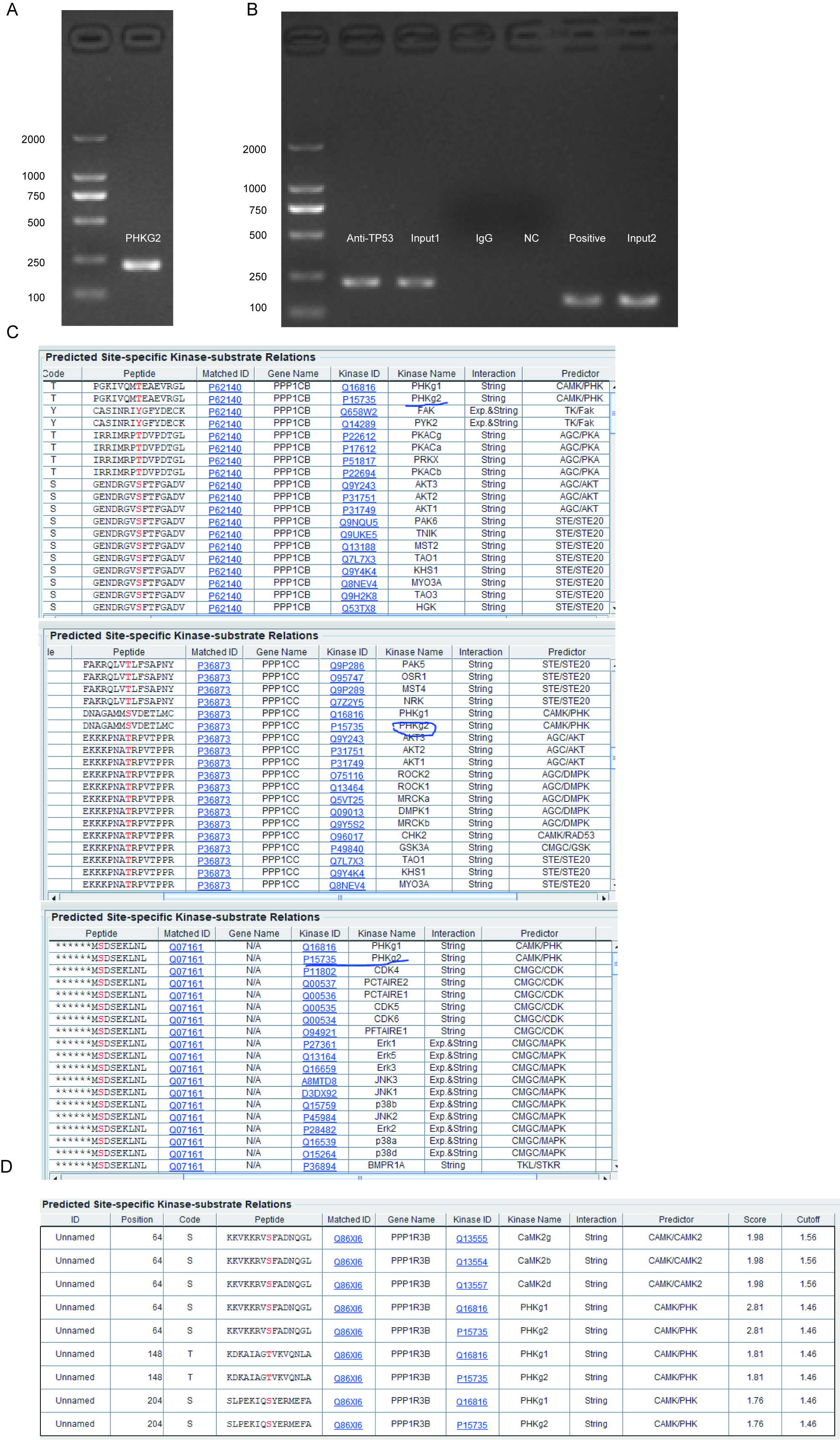

Supplement: Supplementary file 5 — Supplemental Figure 2 [file 41419_2025_7985_MOESM5_ESM.tif]
